# Effect of developmental NMDAR antagonism with CGP 39551 on aspartame-induced hypothalamic and adrenal gene expression

**DOI:** 10.1371/journal.pone.0194416

**Published:** 2018-03-21

**Authors:** Kate S. Collison, Angela Inglis, Sherin Shibin, Soad Saleh, Bernard Andres, Rosario Ubungen, Jennifer Thiam, Princess Mata, Futwan A. Al-Mohanna

**Affiliations:** Department of Cell Biology, King Faisal Specialist Hospital & Research Centre, Riyadh, Saudi Arabia; Universite de Liege, BELGIUM

## Abstract

**Rationale:**

Aspartame (L-aspartyl phenylalanine methyl ester) is a non-nutritive sweetener (NNS) approved for use in more than 6000 dietary products and pharmaceuticals consumed by the general public including adults and children, pregnant and nursing mothers. However a recent prospective study reported a doubling of the risk of being overweight amongst 1-year old children whose mothers consumed NNS-sweetened beverages daily during pregnancy. We have previously shown that chronic aspartame (ASP) exposure commencing *in utero* may detrimentally affect adulthood adiposity status, glucose metabolism and aspects of behavior and spatial cognition, and that this can be modulated by developmental N-methyl-D-aspartate receptor (NMDAR) blockade with the competitive antagonist CGP 39551 (CGP). Since glucose homeostasis and certain aspects of behavior and locomotion are regulated in part by the NMDAR-rich hypothalamus, which is part of the hypothalamic-pituitary-adrenal- (HPA) axis, we have elected to examine changes in hypothalamic and adrenal gene expression in response to ASP exposure in the presence or absence of developmental NMDAR antagonism with CGP, using Affymetrix microarray analysis.

**Results:**

Using 2-factor ANOVA we identified 189 ASP-responsive differentially expressed genes (DEGs) in the adult male hypothalamus and 2188 in the adrenals, and a further 23 hypothalamic and 232 adrenal genes significantly regulated by developmental treatment with CGP alone. ASP exposure robustly elevated the expression of a network of genes involved in hypothalamic neurosteroidogenesis, together with cell stress and inflammatory genes, consistent with previous reports of aspartame-induced CNS stress and oxidative damage. These genes were not differentially expressed in ASP mice with CGP antagonism. In the adrenal glands of ASP-exposed mice, GABA and Glutamate receptor subunit genes were amongst those most highly upregulated. Developmental NMDAR antagonism alone had less effect on adulthood gene expression and affected mainly hypothalamic neurogenesis and adrenal steroid metabolism. Combined ASP + CGP treatment mainly upregulated genes involved in adrenal drug and cholesterol metabolism.

**Conclusion:**

ASP exposure increased the expression of functional networks of genes involved in hypothalamic neurosteroidogenesis and adrenal catecholamine synthesis, patterns of expression which were not present in ASP-exposed mice with developmental NMDAR antagonism.

## Introduction

Aspartame (L-aspartyl phenylalanine methyl ester) is a non-nutritive sweetener (NNS) approved for use in more than 6000 dietary products and pharmaceuticals consumed by the general public including adults and children, pregnant and nursing mothers [1,2]. Despite its widespread use, little is known about the rate of consumption of NNS during pregnancy, or the long-term metabolic and behavioral outcomes for the offspring [3]. However a recent prospective study of more than 3000 mothers and their children reported a doubling of the risk of being overweight amongst 1-year olds whose mothers consumed NNS-sweetened beverages daily during pregnancy [4]. In a rodent study designed to reflect patterns of aspartame consumption in the human population, we have previously shown that aspartame exposure, commencing *in utero* via the mothers diet may adversely affect adulthood glucose homeostasis and spatial cognition [5]. The role of the central nervous system (CNS) in regulating glucose homeostasis has been highlighted by the recent delineation of the gut-brain axis [6], in which N-methyl-D-aspartate receptors (NMDARs) play an important role in maintaining glucose homeostasis in addition to regulating a number of aspects of cognition and behavior [7, 8]. Overactivation of the sympathetic nervous system is an emerging hallmark of diabesity, although the complexity of the mechanisms linking diabetes with heightened sympathoexcitatory activity is an ongoing investigation. Within the CNS, considerable interest has focused on the role of the NMDAR-rich hypothalamus in the control of glucose homeostasis [9–11] behavioral patterns [12–14] and locomotor activity [15]. The hypothalamus links the nervous system to the endocrine system via the hypothalamic pituitary adrenal (HPA) axis, which regulates many systems including energy balance, temperature regulation and the activity of the autonomic nervous system.

NMDARs, one of the fundamental neurotransmitter receptors in the CNS, are glutamate-gated cation channels characterized by their high affinity for glutamate, high calcium permeability and relatively slow activation and deactivation kinetics [16, 17]. Receptor activation requires the binding of glutamate and a co-activator glycine (or serine), together with membrane depolarization; whereas at rest NMDARs are blocked by magnesium ions in a voltage-dependent manner [18], which makes them unique in being both ligand-gated and voltage-sensitive. Additionally, due to their high calcium permeability NMDARs are able to effect lasting modifications in neurochemistry by initiating downstream calcium-dependent effects including long-term potentiation which underpins learning and memory [19]. The expression of NMDA receptor subunits is developmentally regulated, and changes in expression patterns have been shown to correlate with critical periods of CNS development [20] which is vulnerable to fetal programming. Neonatal treatment with the competitive NMDAR antagonist CGP 39551 has been shown to result in long-lasting structural, neurochemical and behavioral alterations in adulthood [21]. It is therefore apparent that NMDARs are critically involved in the regulation of both glucose and energy metabolism as well as many aspects of behavior, learning and memory, which can be modulated by aspartame exposure either during early life or in adulthood [22–24]. Equally of interest is the finding that in addition to their major role in neuronal transmission in the central and autonomic nervous systems, NMDARs are expressed in a variety of non-neuronal tissues, including the pancreas and adrenal glands [25, 26]. A recent study has also demonstrated the presence of NMDARs in fetal pancreatic tissue [27], suggesting the possibility that glucose metabolism may be modulated by NMDARs *in utero*.

Aspartame (ASP), which upon ingestion is rapidly hydrolyzed into its metabolites phenylalanine, aspartate and methanol, has the potential to interact with NMDARs in a number of ways, and has also been shown to increase hippocampal NMDAR subunit expression in rodents chronically exposed to dietary ASP [28]. Firstly, aspartate is a high-affinity NMDAR agonist [29], however some studies have shown that although plasma aspartate levels do rise significantly after ASP consumption [30], the amount of aspartate ingested as ASP is unlikely to promote neurotoxicity [31]. The main effect of ASP consumption in humans is to increase levels of plasma phenylalanine [32]. In rodents, levels of plasma phenylalanine and its metabolite tyrosine also increase following ASP ingestion [33, 34] although to a lesser extent [35]. Phenylalanine competes with tyrosine, the precursor of dopamine for binding sites on the large neutral amino acid transporter (NAAT) at the blood brain barrier (BBB), with the result that brain tyrosine levels are affected and dopamine synthesis may be compromised [36, 37]. Phenylalanine also has the potential to directly interact with NMDARs by competing at the glycine binding site to modulate NMDAR activity [38, 39]. Additionally phenylalanine may modulate glutamatergic signaling by altering CNS regional catecholamine levels, for example dopamine [40,41], which can in turn regulate NMDAR surface expression and activity [42]. Since phenylalanine has the potential to cross both the BBB and the placenta [43] and to preferentially accumulate in the fetus [44], this could affect glutamatergic signaling particularly in mice where the activity of phenylalanine hydroxylase, the enzyme necessary to convert phenylalanine to tyrosine, is not active until the last stages of gestation [45, 46]. High levels of phenylalanine *in utero* have been shown to result in reduced brain and body weight [44] and to incur long-lasting neuronal impairment [47]. Cellular mechanisms which ameliorate neuronal damage include the endogenous production of neurosteroids [48], which in turn are able to allosterically modulate NMDAR activity [49].

In view of the above, and because metabolic and behavioral phenotypes are influenced by environmental variables during perinatal development, we hypothesized that NMDARs may play a role in the ASP-induced perturbations in glucose homeostasis and behavior previously reported by us [5] and others [22–24, 50, 51]. Accordingly we found that early-life treatment with the NMDAR antagonist CGP 39551 [52] normalized the ASP-induced insulin intolerance seen in adult mice exposed to ASP from conception onwards, whilst not altering glucose homeostasis in control diet mice to any significant degree [53]. A second finding from our study was that the behavior of ASP-fed mice with early-life NMDAR antagonism differed from control-diet mice receiving NMDAR antagonism under identical experimental conditions, suggesting a drug/diet interaction occurring early in life with long term consequences. Specifically, whereas control diet mice with NMDAR antagonism exhibited hyperlocomotor behavior consistent with previous reports [21, 54], ASP-diet mice with NMDAR antagonism were hypolocomotive, more immobile, and showed less exploratory behavior than aspartame-diet mice without NMDAR modulation [53]. Markers of anxiety-based behavior were also differentially affected by diet and drug treatment. Spatial cognition, as assessed using the Morris Water Maze (MWM) paradigm was equally affected by ASP diet either in the absence or presence of NMDAR antagonism; and to a lesser extent by the NMDAR antagonist in control diet mice, again consistent with previous studies [55]. Interestingly, Aya-Ramos *et al* [56] recently reported that early maternal separation stress augments ASP-induced hyperglycemia and hyperactive behavior in rats, and further suggested that stress may negatively impact glycemic control and behavior through modulation of the HPA axis.

The aims of the present study were to further elucidate the mechanism(s) behind NMDAR modulation of ASP effects on physiology and behavior. Accordingly, global changes in adulthood hypothalamic and adrenal gene expression, together with serum markers of the HPA axis were examined in response to ASP exposure and/or developmental NMDAR antagonism with CGP 39551.

## Materials and methods

### Animals and treatments

**Fig 1:**
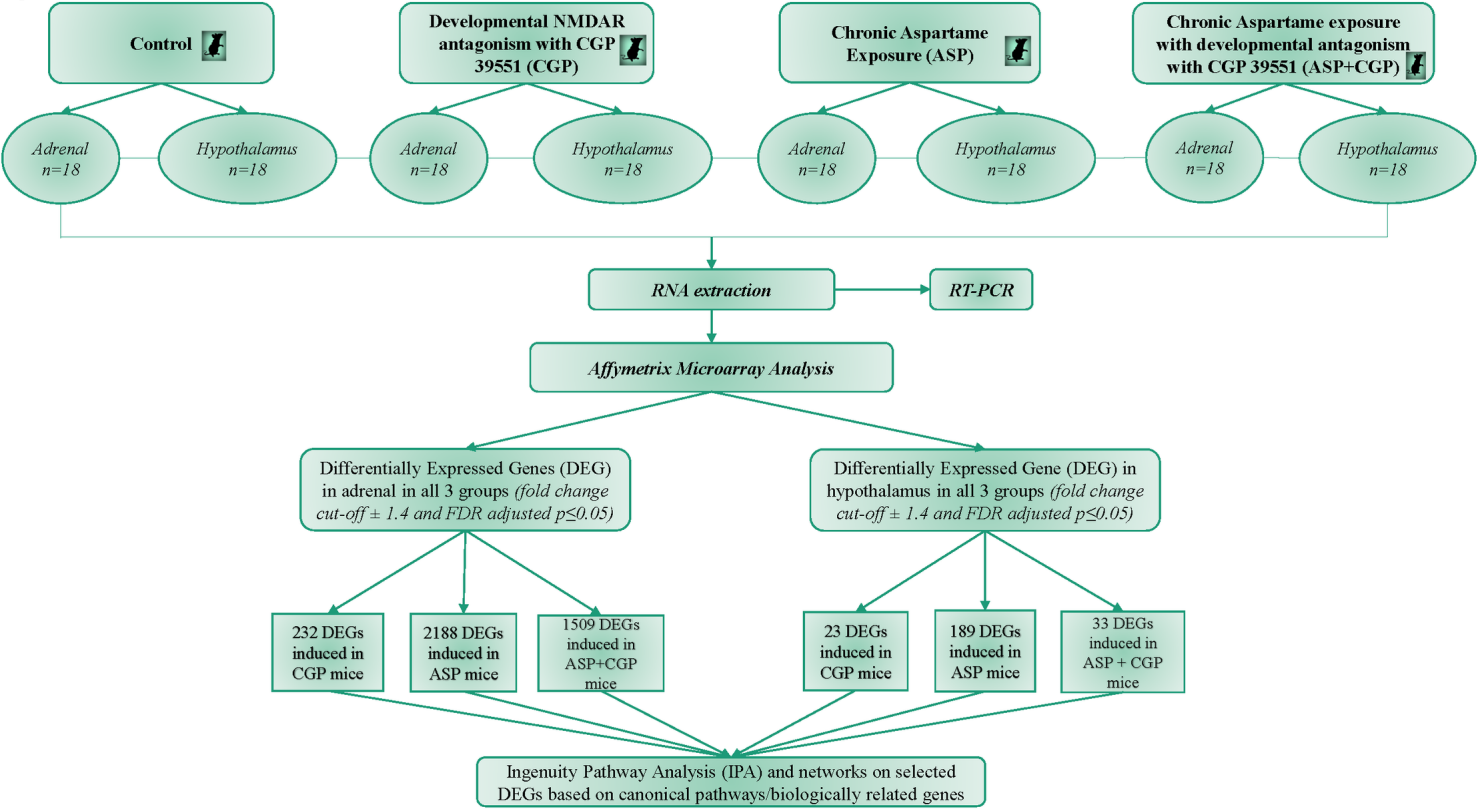
Schematic of experimental design and analysis.

C57BL/6J mice of both sexes were obtained from the Jackson Laboratory (Maine, USA) and housed 3/cage in a controlled environment (pathogen-free conditions of 12 h light/dark cycle, 22 ± 2°C) with standard chow and water available *ad libitum*. The breeding and care of the animals were in accordance with the protocols approved by the Animal Care and Use Committee of the King Faisal Specialist Hospital & Research Centre as previously described [5]. In order to obtain F1 study offspring following developmental NMDAR antagonism with CGP 39551, female breeders were randomly divided into four groups: Control diet (CON), Control diet + developmental NMDAR antagonism with CGP 39551 (CGP), chronic aspartame exposure (ASP), and chronic aspartame exposure + developmental NMDAR antagonism (ASP + CGP). Aspartame (0.25g/L, Sigma Aldrich, MO, USA) was administered in the drinking water as previously described [5]. The dose of the water-soluble anticonvulsant compound CGP 39551 (CGP: Tocris Bioscience, MI, USA) was selected on the basis of previous experiments demonstrating potent biological activity following oral administration [57]. CGP was reconstituted to a stock solution of 8.43 mM by the addition of sterile distilled H_2_O. Tolerance to the separate and combined ASP and CGP treatments were ascertained in pilot studies which monitored the general health, body weight, food and fluid consumption before and during 3 weeks of treatment. Daily fluid intake was estimated in pre-weighed mice by subtracting the initial weight of the filled water bottle from the weight after 4 days of consumption. This value was then divided by the number of days of consumption and the number of mice per cage. Daily food intake was also calculated by the subtraction method. For administration of CGP, 9 week-old females were weighed, and the volume of CGP stock solution required to achieve 5 mg/Kg bw was determined based on the average body weight of the dams per cage, and calculated on the basis of a daily fluid intake of 5 mls/mouse. Breeder females were housed 2 to a cage with an experimentally-naïve male in order to mate. Dams were subsequently monitored and approximately 11 days prior to delivery sires were removed, dams reweighed and the day of parturition was defined as postnatal day 0 (P0). Dams continued to receive the treatment interventions until weaning was completed at P28, whereupon male F1 offspring (no more than 3 animals per litter) in the CON and CGP group were given ad lib plain drinking water and standard chow, and the offspring in the ASP and the ASP+CGP groups were given ad lib drinking water containing ASP (0.25g/L) together with standard chow for the duration of the study. Thus, developmental NMDAR antagonism of offspring mice with CGP 39551 encompassed the period commencing from conception and continued only until weaning, whereas chronic exposure to ASP commenced *in utero* via the mother’s diet and continued throughout the length of the study. Food and fluid intake in the male offspring were measured at 7 weeks of age as previously described [5]. Between weeks 14 and 18 of age, experimental offspring mice took part in behavioral testing, the results of which have been presented in a prior publication [53]. The behavioral testing commenced at 14 weeks of age and comprised of Open Field, Object recognition, Light-Dark transition, and the Morris Water Maze for spatial cognition. A schematic indicating an overview of the experimental design and interventions is presented in Fig 1. At the conclusion of the study (21 weeks of age), overnight fasted animals were humanely euthanized with a mixture of xylazine and ketamine (10 mg/kg and 100 mg/kg respectively) and blood was collected from the inferior vena cava for further analysis of serum components. Male offspring brains were rapidly removed, weighed, rinsed in PBS and positioned to obtain a ventral view. Using the optic tracts and mammillary bodies as visual landmarks, two cuts were made anteriorly at the level of the optic chiasm and posteriorly at the level of the mammillary bodies. The hypothalamus was dissected out as a rectangle to a depth of approximately 2 mm [58], and immediately snap frozen for RNA extraction. Both adrenal glands, located above the kidneys, were excised and snap frozen.

### Evaluation of selective serum components

ELISAs were used to assess the effect of the treatments on changes in overnight fasting serum ACTH, growth hormone (Milliplex Mouse Pituitary Magnetic Bead panel, Millipore USA), corticosterone (Enzo Life Sciences cat# ADI-900-097) and adrenaline (Adrenaline ELISA; Ref: BA E-5100, LDN, Nordhorn, Germany), according to manufacturers’ instructions. For ACTH and growth hormone analysis, samples and standards were processed using the Luminex 100/200 system with related xPONENT software (version 3.1; Luminex Corporation, Austin, TX USSA). A five-parameter logistic regression model with weighting was used to create standard curves (pg/ml) and to calculate the mean of sample concentration from each triplicate. For adrenaline detection, serum adrenaline was first extracted using a cis-diol–specific affinity gel, acetylated to N-acyladrenaline, and then derivatized enzymatically to N-acylmetanephrine. Acylated adrenaline from the standards and samples then competed for a fixed number of antiserum binding sites, later detected by ultrasensitive competitive ELISA. For both corticosterone and adrenaline quantitation, a Varioscan Flash Spectral Scanning multimode microplate reader was used to detect changes in absorbance at 450nm.

### RNA isolation

Total RNA was prepared from snap-frozen hypothalamic and adrenal tissue using Qiagen RNeasy Lipid Tissue Mini Kit Cat # 74804 (Qiagen, CA, USA) according to the manufacturer’s instructions, and stored at -80 ^o^ C. RNA integrity was measured using a 2100 Bioanalyzer instrument and an RNA 6000 Nano LabChip assay (Agilent Technologies, CA, USA). RNA concentrations were determined by absorption at 260-nm wavelength with an ND-1000 spectrometer (Nanodrop Technologies, DE, USA).

### Microarray gene expression analysis

Gene expression was analyzed using 12 GeneChip (R) Mouse Gene 1.0 ST arrays representing 28,853 genes. To minimize the differences of individual variability and increase the statistical power for the identification of potential biomarkers, microarray analysis was performed using equal amounts of purified RNA pooled from all of the study subjects (N = 18 per treatment group), and applied in triplicate. Targets were prepared from male adrenal and hypothalamic tissues and microarrays were processed as described in the Affymetrix GeneChip Whole Transcript Expression Analysis manual using the Ambion WT expression kit and Affymetrix WT Terminal Labeling Kit as per manufacturers' instructions. Briefly, approximately 100ng adrenal and 500ng hypothalamus total RNA was used to synthesize double-stranded DNA with random hexamers tagged with a T7 promoter sequence. The cDNA was used as a template for *in vitro* transcription. In the second cycle cDNA synthesis, random primers were used in reverse transcription to convert the cRNA into single-stranded DNA, which was fragmented, labeled, and hybridized to the arrays in triplicate for 17 hours, then washed and stained using the Fluidics 450 station (Affymetrix, Santa Clara, CA). Arrays were scanned using the Affymetrix 3000 7G scanner and GeneChip Operating Software version 1.4 to produce.CEL intensity files. This software also provided summary reports by which array QA metrics were evaluated including average background, average signal, and 3′/5′ expression ratios for spike-in controls, β-actin, and GAPDH. Microarray data was deposited at the MIAME compliant NCBI gene expression hybridization array data repository (GEO: http://ncbi.nlm.nih.gov/geo) under accession # GSE100325 and GSE100324 (expression data from hypothalamus and adrenal tissue respectively).

### Taqman real-time PCR

Confirmation of microarray results was performed by TaqMan real-time PCR using 96-well microtiter plate in the CFX96 real-time detection system (BioRad Laboratories, Hercules, CA). DEGs that differed significantly (P<0.05) in their regulation between the diet groups' microarray analysis were selected, based on their biological relevance, and validated with the same pooled RNA (n = 18) for qPCR analysis. Each run of TaqMan real-time PCR contained three replicates. 2μg of total hypothalamic and adrenal RNA was reverse transcribed using SuperScript II First strand cDNA synthesis kit with random primers (Life Technologies, Rockville, MD) according to manufacturer’s instructions. The TaqMan primers and probes were synthesized and labeled by Applied Biosystems, USA. S1 Table contains a list of the TaqMan assay ID# and Affymetrix Probeset ID#s for each of the genes. Each TaqMan probe was labeled with 6-carboxy-fluorescein reporter dye at the 5’ end and 6-carboxytetramethylrhodamine fluorescent quencher at the 3’ end to prevent extension by AmlipTaq Gold DNA polymerase. Internal control probe was labeled with a 5’ reporter VIC dye. Primers were designed to span an exon junction, where possible. TaqMan reactions for genes that were assayed consisted of 1X Universal Master Mix, 0.8 μmol/l forward and reverse primers, 0.2 μmol/l probe and 2 μl diluted cDNA in a final reaction volume of 10 μl. The real-time PCR thermal profile consisted of 50 ^o^C for 2 min, followed by 95 ^o^C for 10 min and 40 cycles of 95 ^o^C for 15 min and 60 ^o^C for 1 min. Fluorescence was measured once per cycle at the end of the 60 ^0^c segment. The Bio-Rad CFX96 Manager software (version 1.6) was used to collect and analyze amplification and melting data from the CFX96 Real Time PCR detection system. The relative amount of each transcript was determined on the basis of the threshold cycle (Ct value). Threshold cycle (Ct) data for all target genes and mouse housekeeping gene were used to calculate ^Δ^Ct values [^Δ^Ct = Ct (target gene) − Ct (GAPDH)]. The ^ΔΔ^Ct values were calculated by subtracting the GAPDH from the ^Δ^Ct values of each target. Expression changes between the diet comparisons were calculated for both data. Pearson correlation analysis between the qPCR and microarray data were then displayed as a scatter plot. Statistical significance was assessed using a two-tailed t test assuming unequal variance of the biological replicates.

### Data analysis

Statistical analyses were performed using IBM SPSS statistics software version 20 (SPSS Inc., Chicago, IL). Differential hypothalamic and adrenal gene expression analysis was performed using the Partek Genomic suite software version 6.15 (Partek Incorporated, USA) as previously described [59, 60]. Quality controls data were examined for outliers with respect to other highly related samples by means of Hybridization (Spike Controls) and internal control genes (housekeeping). A histogram was generated using quality controls probe set and Pearson´s correlation helped determine the relationship between arrays. The data were categorized and grouped by means of Principal Component Analysis (PCA). Probe set data were summarized and Robust Multi-Array Average (RMA) algorithm was used for background correction [61] as implemented in the microarray analysis software (MAS). The standard RMA algorithm used the log 2 transformed perfect match (PM) values followed by quantile normalization. The transformed PM values were then summarized by median polish method. The RMA model performs a background correction by fitting a two component model to the PM intensities. The normal-exponential linear model assumes that M = *α*+*β*, where *α* has a normal distribution with parameters *μ* (the mean) and *σ* (the standard deviation); β has an exponential distribution with parameter *λ*(the reciprocal of the mean) [62]. The parameters *α*, *μ* and *σ* are then estimated and the expected value of the signal is estimated, given the observed value of the intensity using the following equation: *E [ε I PM] = PM - μ - λσ*^*2*^ *+ σ{1/√2π exp(-1/2(PM/σ)*^*2*^*/φ(PM/σ)}*. An advantage of this method over the other normalization methods available is that normalization occurs at the probe level (rather than at the probeset level) across all of the selected hybridizations (rather than for each array individually). Quantile Normalization, a non-linear transformation method was used to normalize data which uses the formula *x*_*norm*_ *= F*_*i*_^*-1*^*(F*_*ref*_*(x))*, where *F*_*i*_ is the distribution function of chip *i*, and *F*_*ref*_ is the distribution function of the reference chip. This improves the linearity, normality & homoscedasticity of the data by eliminating noise and extreme expression values. Probesets without unique Entrez gene identifiers were removed from further analysis and values below log 6 were filtered out. For identification of ASP and CGP-induced differentially expressed genes (DEGs) we considered the comparison of four conditions (ASP versus CON; CGP versus CON; ASP+CGP versus CON, and ASP versus ASP+CGP). Significance value of p<0.05 indicated that the gene was differentially regulated by drug or diet. Genes that were regulated in response to the treatments were identified using False Discovery Rate (FDR) method [63] in which p-values were adjusted simultaneously across multiple subgroup comparisons. Contrasts were included in the model based on the comparison of interest. The significant and differentially expressed genes were selected by means of cut-off fold change (>±1.4) and FDR-adjusted ANOVA p-value.

Ingenuity Pathway Analysis (IPA) software (Ingenuity Systems, Redwood City, CA) was performed on genes identified by the microarray analysis as being differentially expressed. Genes with known gene symbols (HUGO) and their corresponding expression values were uploaded into the IPA software, where gene symbols were mapped to their corresponding gene object in the Ingenuity Pathways Knowledge Base (IPKB). The significance of the association between the data set and the canonical pathway was assessed by (1) a ratio of the DEG that mapped to the pathway divided by the total number of genes that mapped to the canonical pathway; and (2) applying an FDR ≤ 0.05 to calculate a *P*-value. Networks of potentially interacting DEGs were identified and placed into node-edge diagrams comprised of focus molecules (DEGs identified by the microarray analysis) and other interacting molecules. Partek was also used to create a gene heatmap of DEGs common to both hypothalamic and adrenal tissues.

### Results

Fig 1 indicates treatments and experimental design. Mean aspartame intake was estimated as 45.44 ± 1.16 mg/Kg bw and 46.59 ± 0.68 mg/Kg bw in the ASP diet mice and the ASP diet mice with developmental NMDAR antagonism group (ASP+CGP) respectively. Food and fluid intake in the offspring were not significantly affected by either diet or drug (Table 1). Detailed analysis of the effects of chronic ASP exposure and NMDAR antagonism on the physiological and behavioral characteristics of adult mice were reported in a previous publication [53], which indicated that the effects of ASP may impact on components of the Hypothalamic Pituitary Adrenal (HPA) axis. Table 1 summarizes the effect of chronic ASP exposure and/or CGP 39551 on overnight fasting serum adrenocorticotropic hormone (ACTH), growth hormone (GH), corticosterone and epinephrine. Levels of ACTH and epinephrine were not significantly affected by the treatments, whereas serum GH, a stress hormone secreted by the anterior pituitary was elevated by 45% in aspartame-exposed mice. Developmental NMDAR antagonism of ASP-exposed mice also lowered levels of corticosterone by 32% but was unaffected by either treatment separately (Table 1, p≤0.05).

**Table 1:** Food and fluid intake data along with serum markers.

	CON			CGP			ASP			ASP + CGP			Sig.
Adrenocorticotropic hormone (pg/ml)	143.78	±	14.63	134.88	±	13.90	153.43	±	18.23	172.68	±	19.29	0.45
Growth hormone (ng/ml)	5.56	±	0.67	5.04	±	0.63	8.14*	±	1.32	4.39	±	0.80	0.02
Corticosterone (ng/ml)	156.18	±	10.48	158.34	±	7.41	149.05**	±	7.40	105.78	±	5.98	0.00
Epinephrine (nM)	5.41	±	0.35	5.67	±	0.48	4.55	±	0.43	4.79	±	0.37	0.20
Food intake (gm/25gm BW)	4.33	±	0.16	5.12	±	0.35	4.84	±	0.28	5.38	±	0.36	0.09
Fluid intake (ml/25gm BW)	4.73	±	0.07	4.70	±	0.18	4.63	±	0.09	4.81	±	0.11	0.78

Data are presented as Mean ± SEM (n = 18). A significance of P-value <0.05 and <0.01 based on t-tests are indicated by * and ** for drug effect within each diet group.

BW: body weight; CON: Control; CGP: developmental NMDAR antagonism with CGP 39551; ASP: chronic aspartame exposure; ASP+CGP: developmental NMDAR antagonism and aspartame exposure.

### Microarray analysis: Hypothalamic gene expression

**Fig 2:**
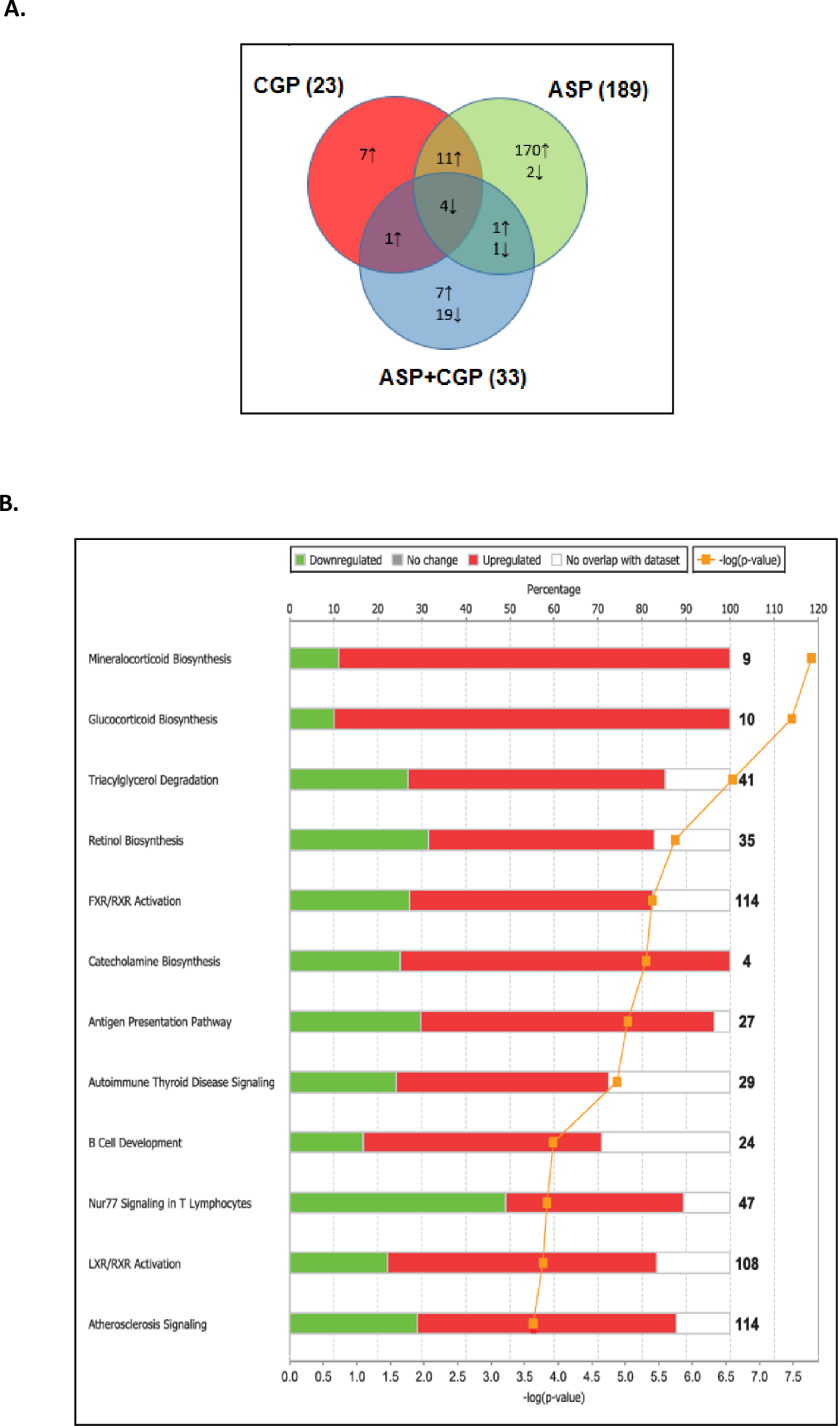
**(A)** Venn diagram representation of hypothalamic DEGs in ASP, CGP and ASP+CGP groups relative to Control. The numbers marked in the diagram indicate the number of genes significantly up regulated (upward arrows) and down regulated (downward arrows). **(B)** Top 12 canonical pathways derived from Ingenuity Pathway Analysis in hypothalamus of ASP-exposed mice. The stacked bar chart displays the percentage of genes that were upregulated (red), downregulated (green) and genes not overlapping in dataset (white) in each canonical pathway. Significance level is scored as–log(p-value) from Fischers exact test. The numerical value at the ends of each bar represents the total number of genes in the canonical pathway. CGP denotes developmental NMDAR antagonism with CGP 39551; ASP denotes chronic exposure to aspartame; ASP+CGP denotes chronic aspartame exposure and developmental NMDAR antagonism with CGP 39551.

To determine the mechanism of action of each treatment, Affymetrix Mouse Gene 1.0 ST expression arrays were used with a False Discovery Rate and a significance level set at 0.05 to identifying 29,149 hypothalamic and 26,533 adrenal gene probesets for further analysis. To identify the impact of chronic ASP consumption and developmental NMDAR antagonism with CGP 39551 (CGP) on adrenal and hypothalamic gene expression, we used ANOVA to compare differences in adulthood gene expression between the 2 tissues. Using a stringency of ± 1.4-fold cut-off in differential expression, we identified a subset of 189 ASP-responsive differentially expressed genes (DEGs) in the adult hypothalamus, and 23 genes which were significantly regulated by CGP (Fig 2A, P≤0.05). Developmental NMDAR antagonism combined with ASP exposure resulted in a further 33 hypothalamic DEGs, 4 of which were common to all treatments. Chronic ASP exposure resulted in the highest number of upregulated hypothalamic DEGs (182 compared to 7 downregulated); whereas antagonism with CGP upregulated 19 genes compared to only 4 which were downregulated, and combined treatment (ASP+CGP) resulted in 24 downregulated and 9 upregulated hypothalamic DEGs (Fig 2A). When we analyzed differential hypothalamic gene expression between groups using a cut-off of ±1.4-fold, we observed that the expression of 225 genes was significantly different between the ASP+CGP and ASP groups (S2 Table).

**Fig 3:**
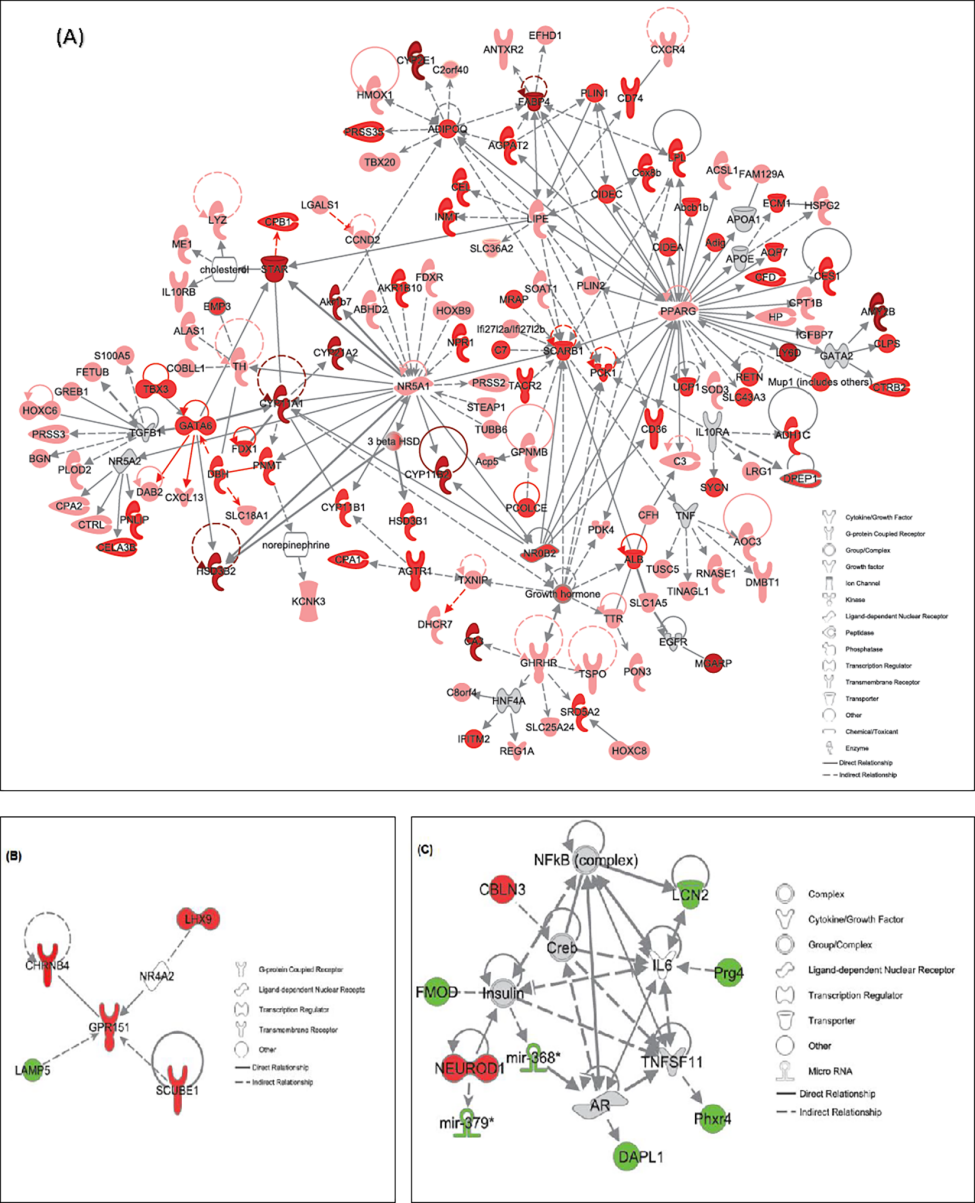
Functional relationship gene networks representing hypothalamic DEGs in response to **(A)** ASP exposure **(B)** developmental antagonism with CGP and **(C)** ASP + CGP. Red-colored and green-colored gene(s) are up-regulated and down-regulated in the dataset. The intensity of red color reflects the extent of differential expression. Only DEGs with ≥±1.4-fold change relative to CON are depicted, P≤0.05).

In order to understand the nature of the hypothalamic transcriptional response to the treatments, Ingenuity Pathway Analysis (IPA) was used to identify the most significant biological processes differentially regulated by chronic aspartame exposure. Fig 2B indicates the 12 most significantly enriched canonical pathways which were significantly overrepresented in the aspartame-treated mice following Fisher’s exact test in IPA. Interestingly, hypothalamic genes associated with mineralocorticoid and glucocorticoid synthesis pathways were upregulated, together with catecholamine synthesis and triacylglycerol degradation. Next, IPA software was used to construct a functional interaction network of DEGs induced by chronic ASP exposure based on fold change relative to Control (Fig 3A). Amongst the DEGs with the highest fold increase in expression in the ASP group only, neurosteroidogenic *Cyp11a1*, *Cyp11b1* (11 β-hydroxylase), *3βHSD*, *Srd5a2* and *StAR* were robustly increased by 29, 66, 31, 9 and 21-fold respectively. Neurosteroidogenesis is required for the physiological response to cellular stress [48, 64]; which is known to be affected by chronic ASP exposure [65]. Of interest, several genes involved in catecholamine synthesis were upregulated such as dopamine beta hydroxylase which converts L-DOPA into norepinephrine (*Dbh*, 6.6-fold), and phenylethanolamine-N-methyltransferase which converts norepinephrine to epinephrine, amongst other reactions (*Pnmt*, 6.8-fold; Fig 3). In the CNS, norepinephrine acts as the main adrenergic neurotransmitter in the sympathetic nervous system [66], and it was interesting to note that expression of these neurosteroidogenic genes were not upregulated in aspartame-exposed mice with developmental NMDAR antagonism (S2 Table). Top stress-associated hypothalamic DEGs included upregulation of resistin (*Rst* 7-fold), *Cyp2e1* (14-fold), *Tspo* (1.7-fold) and *Ly6D* (10-fold, Fig 3A, P≤0.05). Neuroinflammatory DEGs upregulated by ASP exposure included adiponectin (*Adipoq*, 5-fold), *Fabp4* (10-fold), *Scarb1* (5-fold) and *Cd36* (7-fold). Additionally, expression of alcohol dehydrogenase 1, the enzyme which converts methanol to formic acid and formaldehyde was also increased in ASP-exposed mice (Fig 3A). Importantly, all of the aforementioned genes forming the functional network in Fig 3A were not deregulated in ASP-exposed mice with developmental NMDAR antagonism.

We next created a network of hypothalamic genes which were differentially modulated in response to CGP 39551 (Fig 3B). These included a number of upregulated genes involved in neurogenesis including *Gpr151*, *Chrnb4*, *Scube1 and Lhx9*, whereas expression of glycine transporter *Slc6a5*, also known as GlyT2, was reduced by 4-fold relative to control. Combined NMDAR antagonism and chronic ASP exposure upregulated several neuronal genes including cerebellin 3 precursor protein (*Cbln3*) and *Neurod1*. Key down-regulated neuronal DEGs included Lipocalin2 (*Lcn2*), proteoglycan4 (*Prg4*), Fibromodulin (*Fmod*), Per -hexamer repeat gene 4 (*Phrx4*) and death associated protein like 1 (*Dapl1*: Fig 3C, P≤0.05).

Partek software was also used to create a sublist of 13 genes which were deregulated in the hypothalamus of aspartame-exposed mice, and also in ASP-exposed mice with developmental NMDAR antagonism (Table 2, P≤0.05). Of these, 1 gene was upregulated in both groups, 5 genes were downregulated, and 7 genes were upregulated by ASP exposure and down regulated by a combination of ASP+CGP. One of the most important DEG in this category from a biological point of view was hypothalamic proopiomelanocortin (*Pomc*), which is known to be important in the control of energy balance and glucose homeostasis.

**Table 2:** Hypothalamic DEGs induced in the ASP group and also in the ASP+CGP group, relative to control.

Entrez Gene Name	Gene Symbol	ASP	ASP+CGP	p-value
Solute carrier family 6 member 5	*Slc6a5*	-2.70	-3.76	***8*.*44E-08***
BPI fold containing family A member 1	*Bpifa1*	-2.06	-1.97	***5*.*29E-06***
Lysosomal associated membrane protein family member 5	*Lamp5*	-1.46	-1.44	***3*.*28E-05***
microRNA 376c	*mir376c*	-1.42	-1.53	***0*.*047***
Arginine vasopressin	*Avp*	-1.41	-1.46	***1*.*02E-06***
Cytochrome P450 family 2 subfamily F member 1	*Cyp2f1*	1.54	-1.62	***5*.*06E-08***
Transthyretin	*Ttr*	1.67	1.42	***4*.*55E-05***
Growth Hormone	*Gh*	1.68	-4.96	***5*.*62E-14***
Follicle stimulating hormone beta subunit	*Fshb*	1.82	-1.77	***1*.*79E-08***
Proopiomelanocortin	*Pomc*	1.88	-3.18	***3*.*71E-12***
Glycoprotein hormones, alpha polypeptide	*Cga*	2.41	-4.04	***1*.*43E-08***
Luteinizing hormone beta polypeptide	*Lhb*	2.96	-1.84	***2*.*55E-07***
Prolactin	*Prl*	3.20	-9.33	***1*.*39E-08***

DEG: Differentially Expressed Gene; ASP: chronic aspartame exposure; ASP+CGP: chronic aspartame exposure and developmental NMDAR antagonism with CGP39551

In summary, adulthood hypothalamic gene expression was primarily affected by chronic aspartame exposure and to a lesser extent by developmental NMDAR antagonism. ASP exposure robustly elevated the expression of a network of genes involved in hypothalamic neurosteroidogenesis, together with cell stress and inflammatory genes consistent with previous reports of ASP-induced CNS stress and oxidative damage [67].

### Microarray analysis: Adrenal gene expression

**Fig 4:**
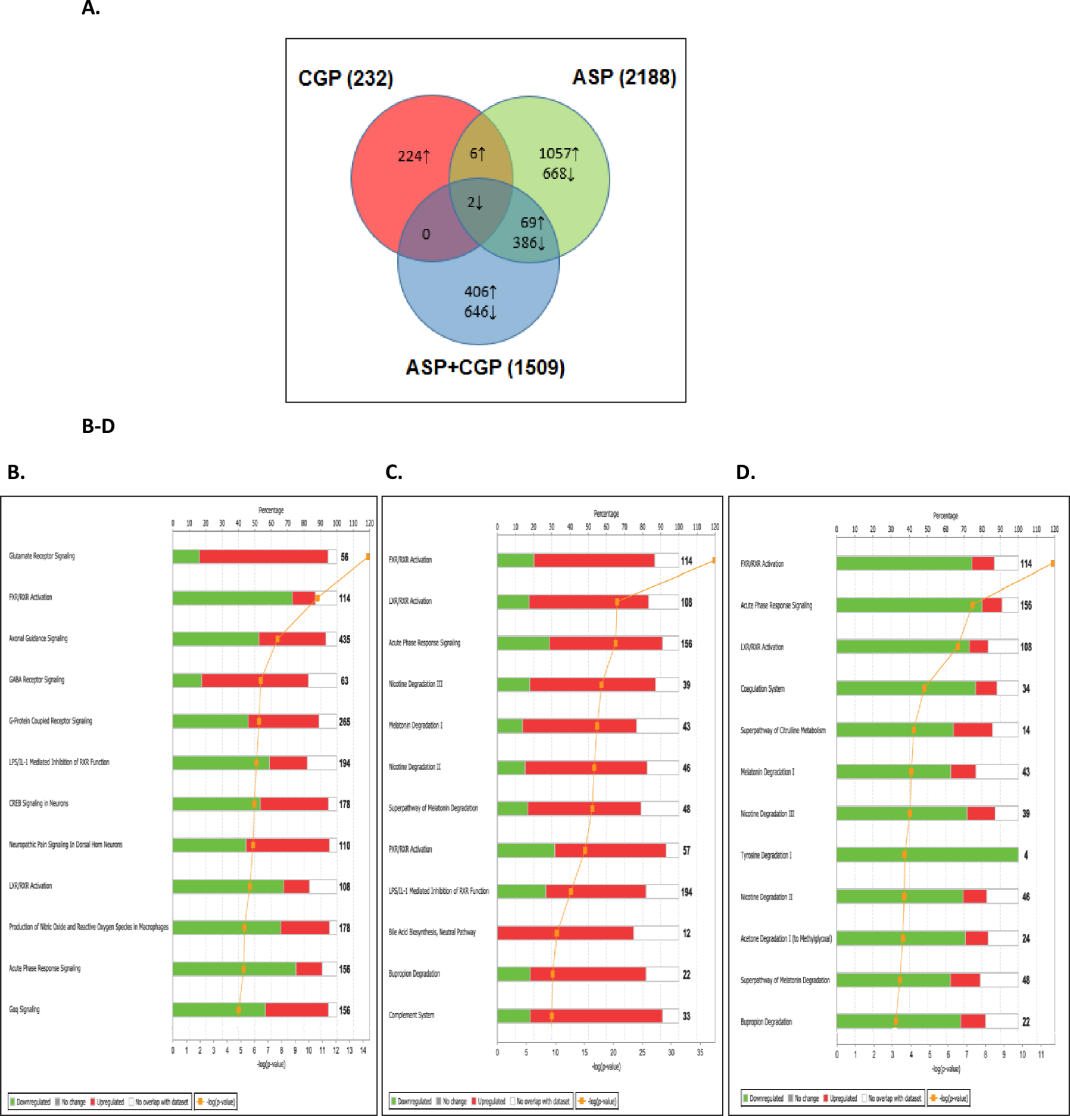
**(A)** Venn diagram representation of adrenal DEGs in ASP, CGP and ASP+CGP groups relative to Control. The numbers marked in the diagram indicate the number of genes significantly up regulated (upward arrows) and down regulated (downward arrows). CGP denotes developmental NMDAR antagonism with CGP 39551; ASP denotes chronic exposure to aspartame; ASP+CGP denotes chronic aspartame exposure and developmental NMDAR antagonism with CGP 39551. Top 12 canonical pathways derived from Ingenuity Pathway Analysis in adrenal glands when exposed to **(B)** Aspartame **(C)** developmental antagonism with CGP and **(D)** ASP + CGP. The stacked bar chart displays the percentage of genes that were upregulated (red), downregulated (green) and genes not overlapping in dataset (white) in each canonical pathway. Significance level is scored as–log(p-value) from Fischers exact test. The numerical value at the ends of each bar represents the total number of genes in the canonical pathway.

The adrenal tissue from these adult mice showed a far greater degree of responsiveness to the treatments (Fig 4A, P≤0.05); resulting in 2188 DEGs after chronic ASP exposure, and 232 adrenal DEGs in mice with developmental NMDAR antagonism with CGP, most of which were upregulated. Combined ASP+CGP treatment resulted in the regulation of 1509 genes. Unlike the hypothalamus, roughly equal numbers of adrenal genes were upregulated and downregulated by ASP exposure (1132 and 1056 DEGs respectively). Developmental NMDAR antagonism with CGP upregulated a subset of 230 DEGs and interestingly, combined ASP+CGP treatment resulted in the up- and downregulation of >1500 adrenal DEGs which were not common to either treatment alone (Fig 4A). Analysis of differential adrenal gene expression between groups using a cut-off of ±1.8-fold, revealed a subset of 1098 genes that were significantly different between the ASP+CGP and ASP groups (S3 Table). Top ASP-induced canonical pathways generated in IPA revealed a highly significant upregulation of adrenal genes involved in glutamate and GABAergic signaling, together with a downregulation in FXR/RXR pathway genes (Fig 4B). The analysis also showed that of the 435 DEGs involved in axonal guidance, approximately 45% were significantly upregulated in response to ASP exposure, whereas equal numbers of the 265 DEGs relating to G protein receptor signaling were up- and down-regulated. Developmental antagonism with CGP 39551 revealed a different pattern of canonical pathways which centered upon genes involved in FXR/RXR and LXR/RXR activation, the acute phase response and nicotine degradation III pathways (Fig 4C); whilst the combination of ASP+CGP promoted the activation of DEGs involved in FXR/RXR and LXR/RXR activation, the acute phase response, coagulation systems and the superpathway of citrulline metabolism (Fig 4D, P≤0.05).

**Fig 5:**
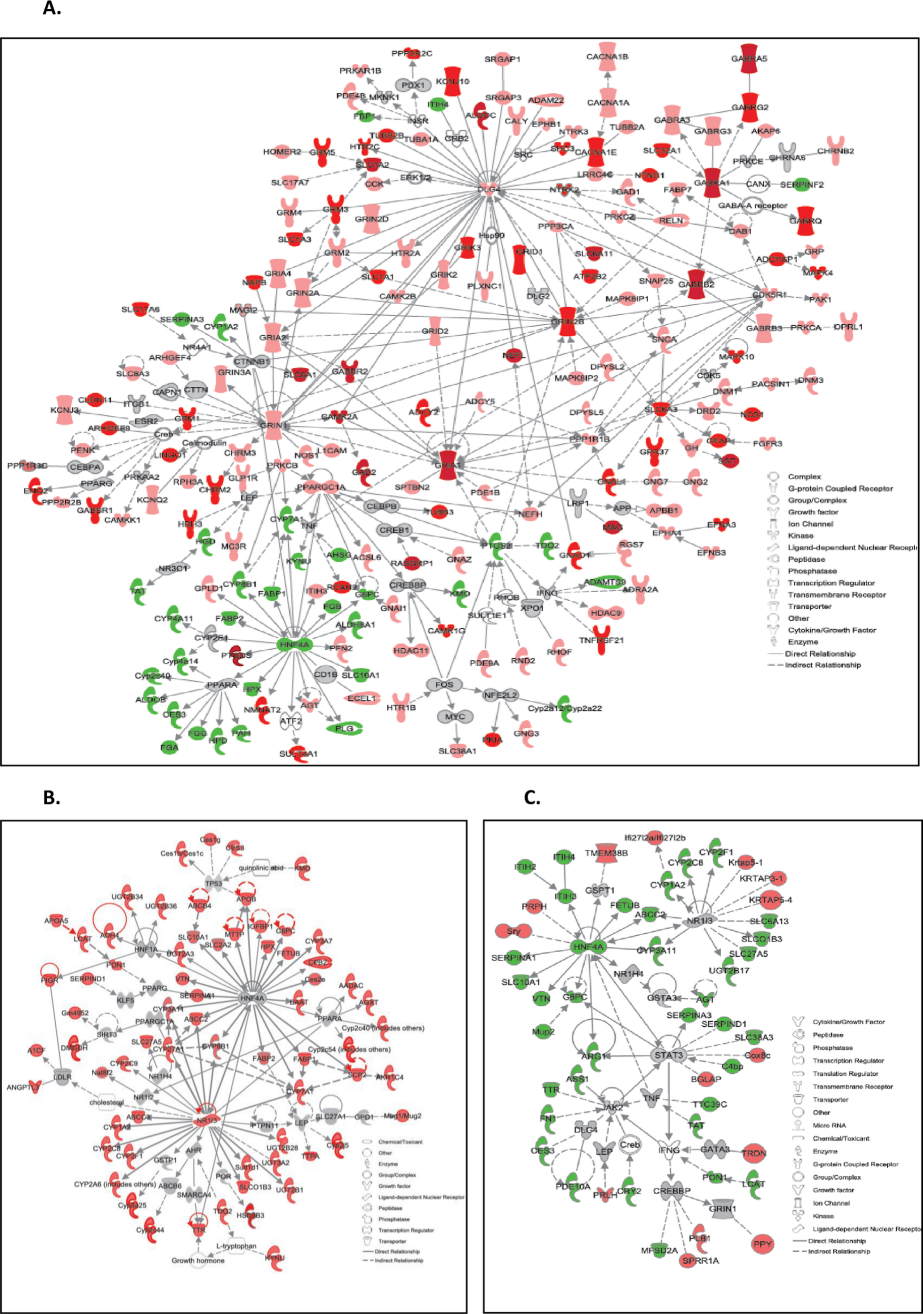
Functional relationship gene networks representing adrenal DEGs in response to **(A)** ASP exposure **(B)** developmental antagonism with CGP and **(C)** ASP + CGP. Red and green color indicates upregulation and downregulation of the genes, respectively and intensity of the color reflects the extent of its differential expression. Only DEGs with ≥±1.8-fold change relative to CON are depicted, P≤0.05).

We next created subsets of adrenal DEGs for network analysis based on fold change relative to Control and using a cut-off of ±≥ 1.8-fold in expression. Fig 5A shows a network created in IPA involving glutamatergic and GABAergic DEGs which were both highly upregulated in response to aspartame exposure. In adrenal chromaffin cells, GABA functions as a paracrine and autocrine modulator of catecholamine synthesis via GABA_A_ receptors [68]; and ASP treatment upregulated the expression of *Gabra1*, *Gabra3*, *Gabrb2*, *Gabrg2* and *Gabra5* by 47, 1.9, 13.5, 6 and 12-fold respectively (P≤0.05). GABA exhibits dual actions in adrenal cells, enhancing catecholamine secretion at low levels whilst attenuating the release of catecholamines elicited by hyperstimulation of nerve impulses. GABA is produced in endocrine cells by 2 isoforms of glutamic acid decarboxylase (GAD): GAD65 (also known as *Gad1*) and GAD67 (*Gad2*), both of which were upregulated in the adrenals of aspartame-exposed mice by 3 and 14-fold compared to Control. GABA transporters *Slc6a1* and *Slc6a11* were also upregulated 36 and 45-fold together with increases in *Slc1a2* (high-affinity glutamate transporter) and *Slc6a3* (dopamine transporter) of 12 and 7-fold respectively. Various ionotropic glutamate receptor subunits including *Grin1* (NR1), *Grin2a* (NR2A) and *Grin2b* (NR2B), and a number of metabotropic glutamate receptors (*Grm2*, *Grm3 and Grm5*) were upregulated in ASP-exposed mice, but not in ASP mice with developmental NMDAR antagonism. Interestingly, ASP exposure upregulated prostaglandin D2 synthase (*PTGDS*) expression, whilst reducing the expression of genes related to the acute phase response and inflammation including *Adamts9*, fibrinogen α,β and γ chains (*Fga*, *Fgb* and *Fgg*), plasminogen (*Plg*), hemopexin (*Hpx*), *Itih4*, *Ptgs2/cox2*, tryptophan 2,3-dioxygenase (*Tdo2*) and Kynureninase (*Kynu*) of between 2- and 3-fold (Fig 5A, P≤0.05). Also downregulated by 2-fold was the expression of adrenal phenylalanine hydroxylase (*PAH*), the enzyme which converts phenylalanine into tyrosine, the precursor of L-DOPA and the catecholamines. Partek software was next used to create a sublist of 99 genes which were deregulated in the adrenals of ASP-exposed mice and also in ASP-exposed mice with developmental NMDAR antagonism (Table 3, P≤0.05).

**Table 3:** Adrenal DEGs induced in the ASP group and also in the ASP+CGP group, relative to control.

Entrez Gene Name	Gene Symbol	ASP	ASP+CGP	p-value
Apolipoprotein A4	*Apoa4*	-3.06	-4.63	**3.06E-10**
ADAM metallopeptidase with thrombospondin type 1 motif 9	*Adamts9*	-2.99	-2.43	**5.99E-07**
Fibrinogen gamma chain	*Fgg*	-2.86	-3.89	**3.07E-10**
Inter-alpha-trypsin inhibitor heavy chain family member 4	*Itih4*	-2.77	-3.06	**2.16E-09**
Murinoglobulin 1	*Mug1*	-2.66	-3.14	**2.43E-09**
Methionine adenosyltransferase 1A	*Mat1a*	-2.65	-3.41	**3.89E-12**
Cytochrome P450, family 2, subfamily c, polypeptide 40	*Cyp2c40*	-2.59	-2.56	**3.13E-07**
Apolipoprotein N	*Apon*	-2.58	-3.21	**2.83E-07**
Prostaglandin-endoperoxide synthase 2	*Ptgs2*	-2.57	-2.33	**5.41E-08**
Homogentisate 1,2-dioxygenase	*Hgd*	-2.51	-2.84	**6.87E-11**
Fatty acid binding protein 1	*Fabp1*	-2.48	-3.83	**7.92E-08**
Prolactin family 8, subfamily a, member 2	*Prl8a2*	-2.44	-2.40	**3.39E-07**
Urate oxidase	*Uox*	-2.44	-2.73	**1.47E-07**
Apolipoprotein B	*Apob*	-2.44	-2.90	**5.55E-10**
Tryptophan 2,3-dioxygenase	*Tdo2*	-2.43	-3.23	**2.33E-08**
Cytochrome P450 family 4 subfamily A member 22	*Cyp4a22*	-2.41	-3.92	**1.33E-09**
Histidine-rich glycoprotein	*Hrg*	-2.41	-3.66	**2.49E-08**
Hemopexin	*Hpx*	-2.38	-2.66	**7.63E-10**
N-acetyltransferase 8 (GCN5-related) family member 2	*Nat8f2*	-2.37	-2.97	**3.05E-11**
Cytochrome P450 family 8 subfamily B member 1	*Cyp8b1*	-2.34	-2.78	**3.81E-09**
Fibrinogen beta chain	*Fgb*	-2.33	-3.12	**2.58E-08**
Betaine—homocysteine S-methyltransferase	*Bhmt*	-2.32	-3.06	**2.38E-09**
4-hydroxyphenylpyruvate dioxygenase	*Hpd*	-2.32	-2.91	**8.68E-12**
C-reactive protein	*Crp*	-2.31	-2.27	**1.31E-07**
Cytochrome P450 family 1 subfamily A member 2	*Cyp1a2*	-2.30	-3.22	**5.00E-10**
Serpin family A member 3	*Serpina3*	-2.28	-2.71	**1.88E-09**
Alpha-2-glycoprotein 1, zinc-binding	*Azgp1*	-2.27	-3.04	**2.66E-09**
Carboxypeptidase N subunit 1	*Cpn1*	-2.26	-2.86	**3.60E-08**
Kynureninase	*Kynu*	-2.25	-2.39	**1.62E-07**
Fibrinogen like 1	*Fgl1*	-2.24	-1.90	**4.68E-05**
Tyrosine aminotransferase	*Tat*	-2.24	-3.20	**1.38E-09**
Carboxylesterase 3	*Ces3*	-2.23	-3.09	**7.92E-10**
Solute carrier family 38 member 4	*Slc38a4*	-2.22	-2.69	**3.65E-10**
Cytochrome P450, family 4, subfamily a, polypeptide 14	*Cyp4a14*	-2.21	-3.40	**7.15E-10**
Glutamyl-prolyl-tRNA synthetase	*Gprs*	-2.20	-1.99	**1.34E-08**
Leucine rich alpha-2-glycoprotein 1	*Lrg1*	-2.19	-2.19	**3.03E-06**
Carbamoyl-phosphate synthase 1	*Cps1*	-2.18	-2.80	**1.30E-11**
Alpha 2-HS glycoprotein	*Ahsg*	-2.18	-2.44	**2.67E-12**
Apolipoprotein C2	*Apoc2*	-2.18	-2.10	**1.39E-07**
Aldehyde dehydrogenase 8 family member A1	*Aldh8a1*	-2.17	-1.83	**6.77E-08**
Plasminogen	*Plg*	-2.16	-2.79	**1.36E-10**
Glucose-6-phosphatase catalytic subunit	*G6pc*	-2.16	-3.36	**9.45E-10**
Cytochrome P450 family 4 subfamily A member 11	*Cyp4a11*	-2.13	-3.06	**1.64E-08**
Fructose-bisphosphatase 1	*Fbp1*	-2.12	-2.93	**2.30E-10**
Hepatocyte nuclear factor 4 alpha	*Hnf4a*	-2.11	-2.07	**3.32E-07**
Carboxypeptidase B2	*Cpb2*	-2.10	-2.31	**2.78E-08**
Aldolase, fructose-bisphosphate B	*Aldob*	-2.1	-3.06	**5.36E-11**
Kynurenine 3-monooxygenase	*Kmo*	-2.09	-2.02	**6.08E-08**
Serpin family F member 2	*Serpinf2*	-2.09	-2.26	**2.19E-09**
Fibrinogen alpha chain	*Fga*	-2.09	-1.91	**2.68E-09**
Mannan binding lectin serine peptidase 2	*Masp2*	-2.07	-2.13	**1.28E-08**
Fatty acid binding protein 2	*Fabp2*	-2.07	-2.02	**5.25E-07**
SAA2-SAA4	*Saa2-Saa4*	-2.06	-2.03	**1.74E-06**
DOT1 like histone lysine methyltransferase	*Dot1l*	-2.06	-2.00	**1.02E-09**
Cytochrome P450, family 2, subfamily a, polypeptide 12	*Cyp2a12*	-2.05	-2.40	**6.31E-07**
Cytochrome P450 family 7 subfamily A member 1	*Cyp7a1*	-2.02	-2.77	**1.87E-10**
Apolipoprotein F	*Apof*	-2.02	-2.22	**2.27E-07**
Aquaporin 9	*Aqp9*	-2.01	-2.02	**7.73E-09**
Cytochrome P450, family 2, subfamily j, polypeptide 5	*Cyp2j5*	-2.00	-2.13	**5.55E-08**
Solute carrier family 10 member 1	*Slc10a1*	-2.00	-2.77	**6.98E-11**
ATP binding cassette subfamily C member 2	*Abcc2*	-2.00	-2.01	**3.35E-09**
Fetuin B	*Fetub*	-1.99	-2.12	**5.71E-08**
Phenylalanine hydroxylase	*Pah*	-1.99	-2.30	**1.36E-09**
Hepcidin antimicrobial peptide	*Hamp(2)*	-1.99	-4.46	**2.40E-09**
Kininogen 1	*Kng1*	-1.98	-2.97	**1.63E-09**
Complement C8 gamma chain	*C8g*	-1.98	-2.33	**1.27E-09**
Cell death-inducing DFFA-like effector b	*Cideb*	-1.97	-2.51	**1.44E-10**
Salt inducible kinase 1	*Sik1*	-1.97	-1.92	**2.32E-09**
Cytochrome P450 family 2 subfamily F member 1	*Cyp2f1*	-1.97	-2.31	**2.16E-10**
Cytochrome P450, family 2, subfamily d, polypeptide 26	*Cyp2d26*	-1.97	-2.87	**8.15E-10**
Apolipoprotein H	*Apoh*	-1.97	-2.65	**2.31E-11**
GC, vitamin D binding protein	*Gc*	-1.96	-2.96	**7.98E-11**
Apolipoprotein A1	*Apoa1*	-1.96	-2.50	**1.09E-08**
Aminoadipate-semialdehyde synthase	*Aass*	-1.95	-2.45	**1.24E-07**
Histidine ammonia-lyase	*Hal*	-1.95	-2.00	**1.12E-08**
Arylacetamide deacetylase	*Aadac*	-1.94	-2.30	**4.40E-08**
Solute carrier family 27 member 5	*Slc27a5*	-1.94	-1.88	**9.59E-08**
VPS37B, ESCRT-I subunit	*Vps37b*	-1.94	-1.83	**2.84E-08**
Serpin family C member 1	*Serpinc1*	-1.93	-2.38	**4.74E-09**
Arginase 1	*Arg1*	-1.93	-2.47	**5.47E-09**
Retinol dehydrogenase 7	*Rdh7*	-1.93	-2.60	**2.00E-07**
Apolipoprotein A2	*Apoa2*	-1.92	-2.92	**5.95E-09**
Complement component 4 binding protein	*C4bp*	-1.92	-1.96	**2.41E-07**
Cystathionine gamma-lyase	*Cth*	-1.91	-1.90	**1.53E-08**
Solute carrier organic anion transporter family member 1B3	*Slco1b3*	-1.91	-1.95	**8.32E-08**
Alpha-1-microglobulin/bikunin precursor	*Ambp*	-1.89	-3.02	**6.34E-10**
SPT2 chromatin protein domain containing 1	*Spty2d1*	-1.88	-2.03	**6.67E-06**
Polo like kinase 3	*Plk3*	-1.87	-2.07	**2.71E-09**
UDP glycosyltransferase family 3 member A1	*Ugt3a1*	-1.86	-2.56	**5.81E-09**
ELOVL fatty acid elongase 2	*Elovl2*	-1.86	-2.71	**1.34E-06**
Coagulation factor II, thrombin	*F2*	-1.85	-2.59	**1.47E-08**
Solute carrier family 25 member 47	*Slc25a47*	-1.85	-2.15	**5.94E-09**
UDP glucuronosyltransferase family 2 member B17	*Ugt2b17*	-1.84	-2.54	**4.46E-07**
JunB proto-oncogene, AP-1 transcription factor subunit	*Junb*	-1.83	-1.86	**3.54E-08**
GTP binding protein overexpressed in skeletal muscle	*Gem*	-1.82	-2.09	**1.43E-08**
Cytochrome P450, family 2, subfamily c, polypeptide 54	*Cyp2c54*	-1.81	-2.15	**5.87E-06**
Major urinary protein 1	*Mup1*	-1.81	-2.50	**1.54E-09**
Solute carrier family 22 member 1	*Slc22a1*	-1.80	-1.83	**5.83E-08**
Cell division cycle 23	*Cdc23*	1.82	6.86	**2.77E-03**

DEG: Differentially expressed gene; ASP: chronic aspartame exposure; ASP+CGP: chronic aspartame exposure and developmental NMDAR antagonism with CGP39551.

Stringency ≥±1.8 fold change in expression, P≤0.05.

Of these, 98 were downregulated in both groups and 1 gene was upregulated by ASP exposure and downregulated by a combination ASP+CGP. Top ASP-induced downregulated adrenal DEGs based on fold change included secretory proteins apolipoprotein A4 (*ApoA4*), *ApoN* and *ApoB*, ADAM metallopeptidase with thrombospondin type 1 motif 9 (*Adamts9*), fibrinogen gamma chain (*Fgg*), inter-alpha-trypsin inhibitor heavy chain family member 4 (*Itih4*) and Murinoglobin1 (*Mug1*)

As stated above, developmental NMDAR antagonism with CGP led to the differential regulation of 232 adrenal genes, and a further 1509 DEGs which were affected by both CGP and ASP combined (Fig 4A). Fig 5B shows the network of adrenal genes most affected by developmental NMDAR antagonism (based on fold-change relative to Control). Functionally, these genes related to steroid and drug metabolism [69], androgen inactivation [70] and Farnesoid X receptor (FXR) activation [71], and included increases in *Cyp7a1*, *Cyp3a25*, *Cyp3a11* and *Cyp2c44* of 3.3, 2.8, 2.6 and 2.0-fold respectively; together with elevated expression of 6 members of the UDP glycosyltransferase superfamily *Ugt2a3*, *Ugt2b1*, *Ugt2b34*, *Ugt2b36*, *Ugt3a1* and *Ugt3a2* (by between 2 and 3-fold; Fig 5B, P≤0.05). The nuclear hormone receptor LXR is important in the regulation of adrenal gland cholesterol homeostasis [72], and a number of LXR-responsive and cholesterol/lipid-related genes were upregulated by developmental NMDAR antagonism including obesity-associated *Angptl3* (2.8-fold), arylacetamide deacetylase (*Aadac*: 2.8-fold); carboxylesterases 1γ, 3α and 3β (*Ces1g*: 2.6-fold; *Ces3a*: 2.0-fold; *Ces3b*: 2.0-fold), paraoxonase 1 (*Pon1*: 2.3-fold) and 3-beta-HSD (*Hsd3b3*: 2.6-fold). Interestingly, the first and rate-limiting enzyme in the Tryptophan-Kynurenine pathway, which is active in neuronal tissues and understood to modulate aspects of inflammation and depression [73] is made by tryptophan 2,3-dioxygenase (*Tdo2*), which was upregulated by 2.5-fold in mice with developmental NMDAR antagonism, together with a doubling in the expression of kynureninase (*Kynu*), a key enzyme in the tryptophan to NAD conversion pathway. Additionally, kynurenine 3-monooxygenase (*Kmo*), which is necessary for the synthesis of NMDAR antagonist quinolinic acid, was upregulated 1.8-fold.

A number of transcripts without assigned gene names were upregulated in ASP-exposed mice with developmental NMDAR antagonism (ASP+CGP group) and were excluded from the analysis, however notable DEGs upregulated in this group included transmembrane protein 38B (*Tmem38B*: 3.6-fold), phospholipase B1 (*PLB1*: 2.1-fold), keratin-associated protein 5–1 (*Krtap5-1*: 2-fold), *Krtap5-4* (1.8-fold), and *Krtap3-1* (1.6-fold); together with a 2-fold increase in triadin (*Trdn*) and pancreatic polypeptide Y (*Ppy*). Downregulated adrenal DEGs affected by the combined treatment included morphological organizational protein vitronectin (*Vtn*: 3.2-fold), DEGs involved in glucocorticoid and mineralocorticoid synthesis (*Lcat*: 2.6-fold) and angiotensinogen (*Agt*: 2-fold), a 2-fold reduction in transthyretin (*Ttr*), argininosuccinate synthase 1 (*Ass1*) and drug-metabolizing *Cyp3a11*. Fig 5C is an IPA generated network featuring molecular interactions between 14 upregulated and 36 downregulated adrenal DEGs modulated by NMDAR antagonism in ASP-exposed mice (P≤0.05). In summary, adrenal gene expression was affected by the treatments to a far greater extent than the hypothalamus. Whereas chronic ASP exposure upregulated hypothalamic genes involved in neurosteroidogenesis, inflammation and lipid metabolism, adrenal pathways most affected by aspartame included GABAergic and glutamatergic signaling, and catecholamine biosynthesis. Developmental NMDAR antagonism with CGP affected hypothalamic genes involved in gonadotropin-releasing hormone signaling and adrenal drug and cholesterol metabolism.

### Commonalities in differential gene expression between the hypothalamus and adrenal tissues

**Fig 6:**
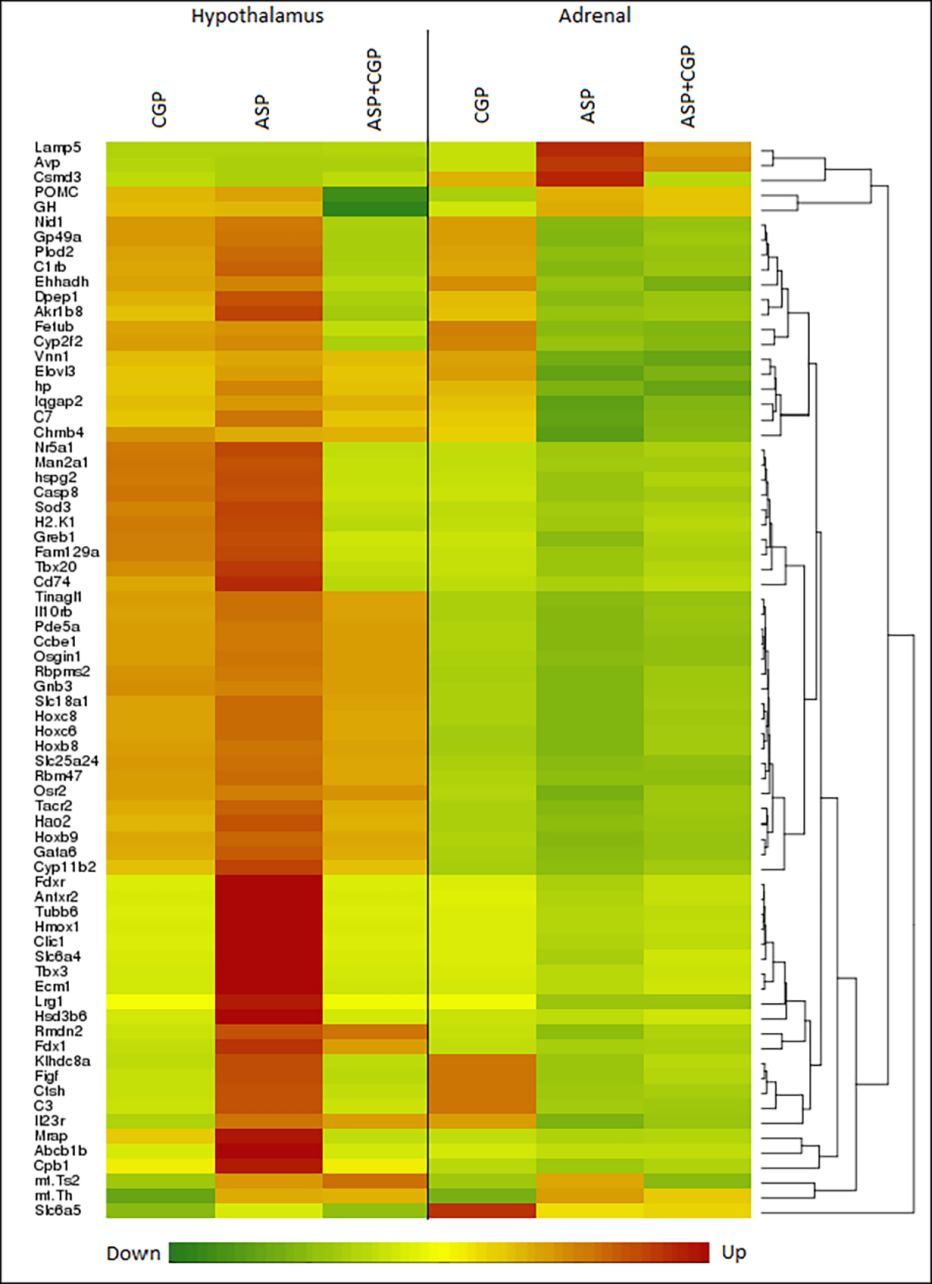
72 differentially expressed genes common to hypothalamus and adrenal glands were hierarchically clustered and illustrated in a heat map (stringency ≥±1.4 fold change in expression, P<0.05). Expression levels are represented by a color scale from green (low) to red (high) indicated at the bottom of the heatmap. CGP denotes developmental NMDAR antagonism with CGP 39551. ASP denotes chronic aspartame exposure. ASP+CGP denotes Aspartame exposure and developmental NMDAR antagonism with CGP 39551.

A number of genes were affected by chronic exposure to ASP in both the hypothalamus and the adrenal gland. Fig 6A is a gene heatmap of DEGs common to both tissues with a cut-off value of >±1.4-fold change. Interestingly almost all of the DEGs which were upregulated by ASP in the hypothalamus, were downregulated in the adrenal gland with the exception of three: *POMC* and *GH* which were upregulated in both, and *Slc6a5* which was downregulated in both. Interestingly, glycine transporter gene *Slc6a5* (GlyT2) was downregulated by chronic ASP exposure regardless of NMDAR antagonism in both tissues, suggesting a central mechanism for ASP-induced metabolic deregulation. Conversely, notable genes with an inverse expression relationship between the tissues included carboxypeptidase 1 (*Cpb1*), melanocortin 2 receptor accessory protein (*Mrap*), hydroxy-delta-5-steroid dehydrogenase, 3 beta- and steroid delta-isomerase 6 (*Hsd3b6*: all upregulated in the hypothalamus); and arginine vasopressin (*Avp*) and leucine-rich alpha-2-glycoprotein 1 (*Lrg1*: both upregulated in the adrenal tissue).

**Fig 7:**
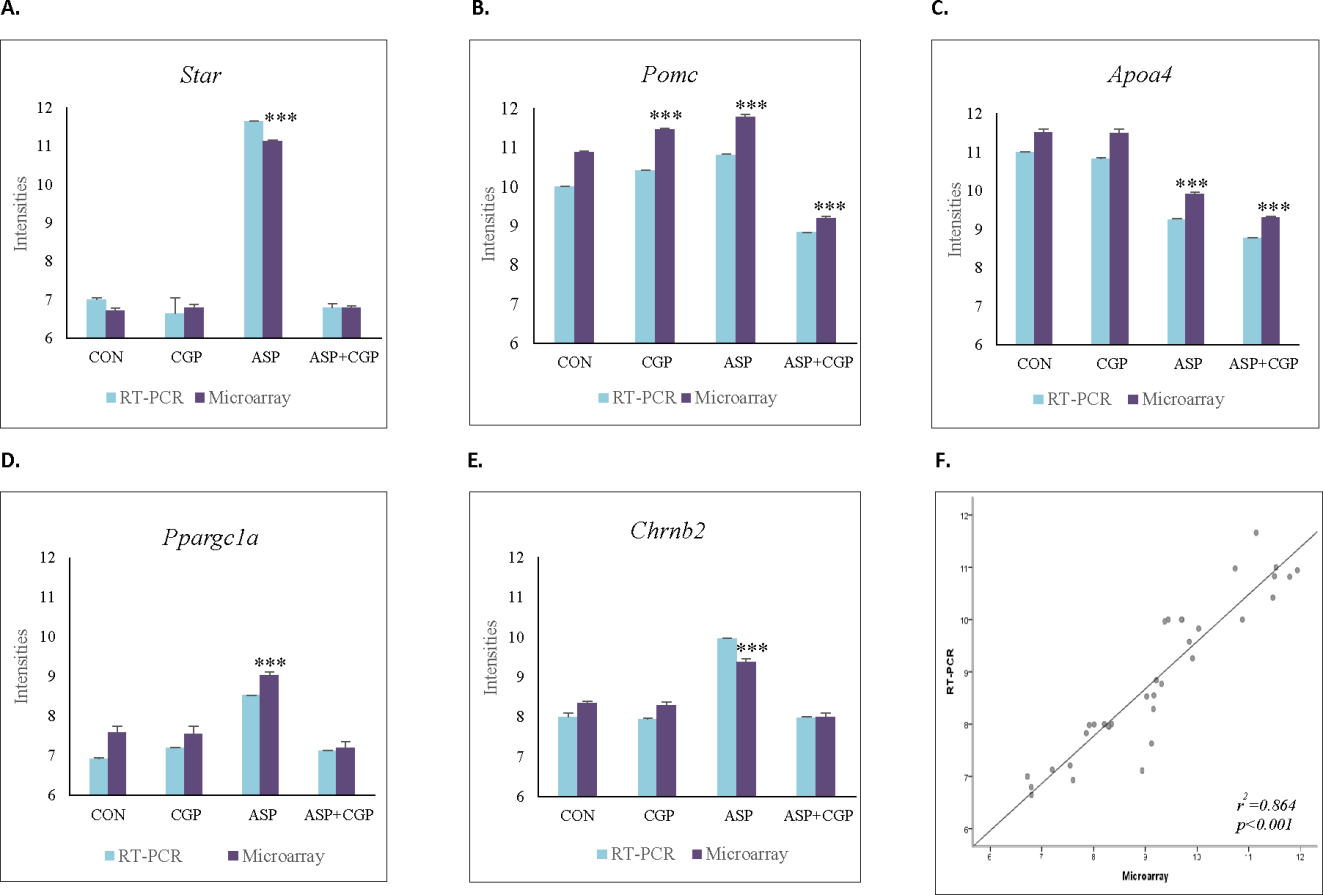
Expression plots of selected genes between RT-PCR and Microarray **(A)** Steroidogenic Acute Regulator, *Star* **(B)** Proopiomelanocortin, *Pomc* **(C)** Apolipoprotein A4, *Apoa4* **(D)** Peroxisome Proliferator Activated Receptor Gamma Coactivator 1 Alpha, *Ppargc1a* **(E)** Cholinergic Receptor Nicotinic Beta 2, Chrnb2. CON denotes Control; CGP denotes developmental NMDAR antagonism with CGP 39551; ASP denotes chronic exposure to aspartame; ASP+CGP denotes developmental NMDAR antagonism and aspartame exposure. Significance of group against Control is represented as *** at p-value <0.001. **(F)** Scatter-plot presentation of changes in expression of 13 selected genes as measured by microarray analysis and RT-PCR.

TaqMan real-time PCR was used to confirm our microarray results using a selection of hypothalamic and adrenal genes randomly chosen based on biological relevance (Fig 7A–7E). Pearson correlation coefficients between the microarray analysis and real-time PCR were calculated. Thirteen genes were analysed for expression by TaqMan PCR, the data obtained from RT-PCR correlated well with the microarray data. (Fig 7F, r^2^ = 0.864, P≤ 0.001). A complete list of these genes and TaqMan Primer assays is given in S1 Table.

### Discussion

Our previous studies have shown that chronic ASP exposure commencing *in utero* may detrimentally affect adulthood glucose metabolism and aspects of cognition and behavior [5], and that this can be modulated by developmental NMDAR blockade with the anticonvulsant NMDAR antagonist CGP 39551 [53]. Because glucose homeostasis and anxiety-related behavior are coordinated in part by the HPA axis [6, 74], and since augmented hypothalamic NMDAR activity has been shown to contribute to hyperactivation of the HPA axis [75], which can be modulated by NMDAR antagonism [76], we elected to examine changes in global gene expression in the hypothalamus and adrenal gland of these animals. The main outcomes of the present study were firstly that chronic ASP exposure affected adulthood gene expression to a far greater extent than developmental NMDAR antagonism, as was to be expected. Secondly, the separate and combined treatments affected adulthood adrenal gene expression to a greater extent than hypothalamic gene expression. Levels of serum ACTH and Growth Hormone (GH), indicative of pituitary activity, were differentially affected to the effect that ASP increased GH levels but not ACTH, and neither were affected by CGP.

The main effects of ASP exposure on hypothalamic gene expression was to robustly upregulate genes involved in the key steps of neurosteroidogenesis, which is known to be required for the physiological response to stress, including *StAR*, rate-limiting *Cyp11a1* (P450scc), *3βHSD* and *Srd5a2* which sequentially converts cholesterol firstly into pregnenolone followed by progesterone and then into 5α-dihydroprogesterone [77]. Additionally, expression levels of *Cyp11b1*, which converts 11-deoxycorticosterone into corticosterone, was also upregulated. Neurosteroids, unlike peripheral hormonal or circulating steroids, act as neurotransmitters capable of rapidly altering receptor-activated neuronal excitability in order to regulate many physiological processes including the HPA axis; and inhibition with finasteride is sufficient to prevent stress-induced anxiety-like behaviors in mice [78]. The biosynthesis of neurosteroids is tightly regulated by neurotransmitters (such as glutamate, GABA and melatonin), pituitary hormones, and various neuropeptides [79]. Functionally, neurosteroids can rapidly modulate GABAergic receptor function by acting locally and non-genomically. The HPA-modulating effects of these compounds depend firstly on the type and local concentration of the neurosteroid (i.e., agonist or antagonist), the location of the receptors (synaptic or extrasynaptic), and their subunit composition [80]. As well as being pivotal in regulating aspects of neurite growth, survival and apoptosis [81], neurosteroid production increases as a result of stress, and their effects are believed to be largely anxiolytic and antidepressant [82]. Previous studies in C57bl/6J mice have demonstrated that under normal conditions, low levels of neurosteroids potentiate inhibitory effects of GABA on corticotrophin releasing hormone-expressing (CRH) neurons; however following stress, neurosteroids activate the HPA axis promoting a collapse of the CRH neuronal chloride gradient resulting in excitatory GABAergic transmission [78]. Additionally, neurosteroids such as pregnenolone sulfate are capable of interacting with NMDARs in a subunit-dependent manner [64, 83], to modulate long term potentiation [84].

Chronic ASP exposure also upregulated hypothalamic DEGs involved in catecholamine synthesis including rate-limiting tyrosine hydroxylase (TH) which converts tyrosine into dopamine, dopamine β hydroxylase which converts L-DOPA into norepinephrine, and phenylethanolamine-N-methyltransferase which converts norepinephrine to epinephrine. ASP is rapidly metabolized into phenylalanine, aspartate and methanol; and phenylalanine can be converted into tyrosine via hepatic phenylalanine hydroxylase (PAH). Tyrosine itself cannot be synthesized in the brain and has to be actively transported across the mature BBB via neutral amino acid transporter (NAAT) complexes, and excess phenylalanine has the potential to compete for binding sites on NAATs, resulting in reduced CNS tyrosine levels and DOPA synthesis [36]. Part of the function of the hypothalamus is to sense levels of nutrients and hormones in order to maintain energy homeostasis, and the BBB in this area is specialized to create a more permeable interphase [85]. Additionally, the BBB is not fully formed during development and studies have shown that raising tyrosine concentrations by injecting the amino acid directly into rodent brain rapidly increases L-DOPA production [86]. Therefore it is possible that chronic ASP exposure commencing *in utero* could directly affect the expression of DEGs involved in hypothalamic catecholamine biosynthesis. Equally of interest is the fact that tyrosine hydroxylase is required for the normal development of pancreatic beta cells, which are known to express NMDARs [25,27] and to be functionally modulated by non-neuronal catecholaminergic signaling [87].

ASP ingestion has been associated with an increase in brain oxidative stress [88, 89]. We found a number of stress-associated and neuroinflammatory hypothalamic ASP-responsive DEGs including resistin, *Cyp2e1*, *Tspo*, *Pomc* and *Ly6D*, adiponectin, *Fabp4*, *Scarb1* and *Cd36*. Pomc is also known for its key functions in regulating energy and glucose homeostasis [90] and hypothalamic glucose sensing [91], whilst centrally-administered adiponectin has been shown to modulate hypothalamic Pomc neurons in a glucose-dependent manner [92]. In addition to its pro-inflammatory role, resistin has also been shown to modulate hepatic insulin sensitivity through both a direct action on the liver, and through hypothalamic mechanisms [93]. Furthermore, hypothalamic CD36 has been demonstrated to play an important role in fatty acid sensing and the maintenance of glucose homeostasis [94]. The possibility therefore exists that the changes in hypothalamic gene expression observed in response to ASP treatment could also have contributed to its potential to alter glucose homeostasis. Interestingly both competitive and uncompetitive NMDAR antagonists have been shown to alter cerebral glucose utility in many areas including thalamic nuclei [95], which may in turn affect blood glucose levels [91]. It is equally plausible that the ASP and CGP treatments could have modulated maternal and/or fetal gene expression in other glucocentric tissues such as the pancreas which is known to express functional NMDARs [25, 27]. Of importance is the finding that *in vivo*, the anticonvulsant NMDAR antagonist dextromethorphan (DXM) significantly improves pancreatic islet insulin content and enhances glucose tolerance when administered to db/db mice in the drinking water [25]. Moreover despite its known neuroprotective effects [96], acute [97, 98] or postnatal [99] administration of DXM has been shown to alter NMDAR gene expression [99], and to cause dose-dependent hyperlocomotion, impaired spatial learning [97, 99], and either anxiolytic or anxiogenic behavior depending on the dosage administered [98].

The main effect of chronic ASP exposure on adrenal gene expression was to upregulate GABAergic and Glutamatergic gene expression. In the brain, GABA is the main inhibitory neurotransmitter however in endocrine tissues such as the adrenal and pancreatic glands GABA may function as a para/autocrine factor [100]. In adrenal chromaffin cells, GABA receptors are mainly composed of pentamers made up of α3β2/3γ2 subunits [68], and the expression of all 3 transcripts were upregulated in ASP-exposed mice. Neuronal GABA is synthesized from glutamic acid by glutamate decarboxylase (GAD), an enzyme which is encoded by the product of two different genes: GAD65 and GAD67 both of which were also upregulated in the adrenals of ASP-exposed mice, as were GABA transporters *Slc6a1* and *Slc6a11*. Recent studies have shown that GABA might have a dual function in adrenal cells: inducing catecholamine secretion potentially leading to increased blood glucose levels [68, 101], and because it reduces the membrane depolarization resulting from electrical nerve stimulation, GABA can attenuate the large release of catecholamine which would be elicited by a barrage of nerve impulses [68].

In addition to its function as a conventional endocrine gland, the adrenal medulla is considered to be a modified neuronal tissue the activity of which is controlled by sympathetic preganglionic autonomic activity [102]. Adrenal medullary cells are modified postganglionic neurons that have lost their axons and dendrites, receiving innervation from corresponding preganglionic fibers driven by glutamatergic activation of the hypothalamic paraventricular nucleus [103]. However, upon activation, instead of releasing neurotransmitters adrenal medullary cells secrete hormones which contribute directly to the control of blood pressure and heart rate. Adrenal medullary tissue has been shown to express functionally active metabotropic glutamate receptors [104,105], and we found that expression of metabotropic glutamate receptors *Grm2*, *Grm3* and *Grm5* were upregulated by aspartame exposure in adrenal tissues, together with several ionotropic glutamate receptor subunits including *Grin1* (NR1), *Grin2a* (NR2A) and *Grin2b* (NR2B). Interestingly, ASP ingestion has previously been shown to increase the expression of rat hippocampal NR2B to levels significantly higher than that induced by the ingestion of monosodium glutamate [106], further confirming an interaction between aspartame and NMDA receptors with the likelihood of modulation by NMDAR antagonists. Our analysis also revealed an increase in adrenal prostaglandin D2 synthase (*PTGDS*) gene expression in ASP-exposed mice. Preferentially expressed in the brain, adrenal PTGDS is an enzyme that catalyzes the conversion of prostaglandin H2 (PGH2) to prostaglandin D2 (PGD2), an increase in which has been shown to stimulate cyclic-AMP-dependent calcium influx in cultured bovine adrenal cells [107]. Intracellular calcium levels have been shown to directly influence adrenal catecholamine secretion [108], however in our study we did not detect a statistically significant change in epinephrine levels in the serum of ASP-exposed mice.

Although developmental NMDAR antagonism with CGP affected adulthood gene expression to a lesser extent than chronic ASP exposure, with approximately 10-fold less differential gene expression compered to ASP, several important adrenal and hypothalamic genes were affected by the treatment. A number of hypothalamic genes involved in neurogenesis were upregulated including *Gpr151*, a G-protein coupled orphan receptor of unknown function expressed in many areas of the brain including the paraventricular nucleus of the thalamus [109]. Levels of LIM homeobox transcription factor *Lhx9*, a developmental gene believed to be involved in hypocretin neuron specification [110] were increased by CGP, as well as CHRNB4, a nicotinic acetylcholine receptor subunit involved in the development of tolerance to nicotine [111]. In the adrenal gland, developmental NMDAR antagonism led to an increase in the expression of many genes involved in steroid and androgen biotransformation including a robust upregulation of the oxidoreductase *Cyp7a1* (Cholesterol 7 alpha-hydroxylase) and several uridine glucuronosyltransferases (UGTs). The glucuronidation reaction, catalyzed by membrane-bound UGTs, involves the transfer of the ubiquitous co-substrate UDPGA to hydrophobic molecules such as steroids, leading to the formation of glucuronide derivatives which can no longer interact with their receptors [112].

In contrast, developmental NMDAR antagonism of ASP-exposed mice with CGP 39551 led to a different pattern of gene expression. Hypothalamic neuronal genes such as neurogenic differentiation 1 (*Neurod1*) and cerebellin 3 precursor protein (*Cbln3*) were upregulated in ASP+CGP mice, whereas a number were downregulated including *Dapl1*, *Fmod*, *Lcn2*, *Prg4* and *Phxr4*. Neurod1 is a transcription factor which regulates expression of the insulin gene [113], and mutations in Neurod1 are associated with type 2 diabetes [114]. Cerebelin in the brain modulates synaptic structure formation whereas in peripheral tissues it regulates catecholamine secretion and also may modulate insulin secretion [115]. In adrenal tissue, chronic ASP exposure of NMDAR antagonized mice resulted in the upregulation of TMEM38B and a number of keratin associated proteins, together with the downregulation of angiotensinogen and arginosuccinate synthase 1. TMEM38B encodes an intracellular monovalent cation channel (TRICB) that functions in the maintenance of intracellular calcium levels whereas angiotensinogen–the precursor of angiotensin 1 and 2 –indirectly inhibits calcium extrusion through the Na+/Ca2+ exchanger and thus has the potential to regulate adrenal aldosterone secretion [116].

Lastly we identified a subset of DEGs which were commonly regulated by chronic ASP exposure in both the hypothalamus and the adrenal gland, and found an interesting inverse relationship between expression of these genes within the tissues in the sense that DEGs upregulated in the hypothalamus were downregulated in the adrenal gland, with only 3 exceptions. These included POMC and growth hormone (GH) together with the neuronal glycine transporter *Slc6a5* (GlyT-2). Whereas *POMC* and *GH* were both upregulated in hypothalamic and adrenal tissues in response to ASP, levels of expression of hypothalamic *Slc6a5* were downregulated by ASP exposure as well as by CGP 39551. This is of interest because glycine is a co-agonist of the NMDA receptor and is required for efficient voltage gating [117]; whereas phenylalanine, a metabolite of aspartame can also interact with NMDARs at the glycine binding site. The possibility exists therefore that deregulation of Slc6a5 expression may form part of the mechanism by which NMDAR antagonism modulates ASP-induced gene expression. Amongst the most notable DEGs which were inversely expressed in the 2 tissues as a result of ASP, arginine vasopressin (AVP) was downregulated in the hypothalamus and upregulated in the adrenal gland where it is known to act as an autocrine/paracrine regulator of adrenal secretion [118]. AVP is an antidiuretic hormone which plays a key role in maintaining osmolality and blood pressure. A third function is its involvement in modulating social behaviors in rodents, and previous studies have shown that exposure to conditioned fear stimuli suppresses AVP production, whereas pretreatment with the NMDAR antagonist MK-801 blocks recall of the emotional memory associated with the attenuated AVP [118]. Therefore AVP represents another DEG which was deregulated by ASP in several tissues and which may also be modulated by NMDAR antagonism.

In conclusion, we have previously shown that developmental NMDAR antagonism with CGP 39551 may modulate ASP-induced deregulation of glucose homeostasis, and may impact on aspects of behavior. The present work identifies changes in hypothalamic and adrenal gene expression associated with these physiological changes, and indicates that ASP may modulate hypothalamic neurosteroidogenic gene expression and adrenal catecholamine-related gene expression, which did not occur in ASP-exposed mice with developmental NMDAR antagonism. Further studies are warranted to examine the effects of these treatments on other NMDAR-expressing tissues, such as the pancreas for example.

## Supporting information

S1 Table Taqman assay IDs and their corresponding Affymetrix probeset IDs. (PDF)

S2 Table Differential changes in hypothalamic gene expression in ASP+ CGP treated mice relative to ASP diet mice (≥±1.4 fold change, p< 0.05). (PDF)

S3 Table Differential changes in adrenal gene expression in ASP+CGP treated mice relative to ASP diet mice (≥±1.8 fold change, p <0.05). (PDF)
