# Supplementary material for: Effect of developmental NMDAR antagonism with CGP 39551 on aspartame-induced hypothalamic and adrenal gene expression
Source: PLoS One. 2018 Mar 21;13(3):e0194416. doi: 10.1371/journal.pone.0194416 (PMC5862471; doi:10.1371/journal.pone.0194416)
Supplement: S1 Table — (PDF) [file pone.0194416.s001.pdf]

S1 Table: Taqman assay IDs and their corresponding Affymetrix probeset IDs

Gene symbol	Gene name	Taqman assay ID	Affymetrix Probeset ID	Sample
Actb	Actin Beta	Mm00607939_s1	10535381	Control
Apoa4	Apolipoprotein A-IV	Mm00431814_m1	10585010	Adrenal
Atg5	Autophagy related 5	Mm01187303_m1	10362922	Adrenal
Chrnb2	Cholinergic receptor, nicotinic, beta polypeptide 2	Mm00515323_m1	10499643	Adrenal
Cyp7a1	Cytochrome P450, family 7, subfamily a, polypeptide 1	Mm00484150_m1	10511375	Adrenal
Gabra1	Gamma-aminobutyric acid (GABA) A receptor, subunit alpha 1	Mm00439046_m1	10385297	Adrenal
Gapdh	Glyceraldehyde-3-phosphate dehydrogenase	Mm99999915_g1	10480032	Control
Gria1	Glutamate receptor, ionotropic, AMPA1 (alpha 1)	Mm00433753_m1	10376245	Adrenal
Lamp5	Lysosomal-associated membrane protein family, member 5	Mm00512854_m1	10476482	Hypothalamus
Pomc	Pro-opiomelanocortin-alpha	Mm00435874_m1	10394240	Hypothalamus
Ppargc1a	Peroxisome proliferative activated receptor, gamma, coactivator 1 alpha	Mm01208835_m1	10529977	Adrenal
Slc6a3	Solute carrier family 6 member 3(nuerotransmitter transporter, dopamine)	Mm00438388_m1	10406050	Adrenal
Slc6a5	Solute carrier family 6 member 5(nuerotransmitter transporter, glycine)	Mm01202538_m1	10553430	Adrenal & Hypothalamus
Srd5a2	Steroid 5 alpha-reductase 2	Mm00446421_m1	10452854	Hypothalamus
Star	Steroidogenic acute regulatory protein	Mm00441558_m1	10571054	Adrenal
