# Supplementary material for: Effect of developmental NMDAR antagonism with CGP 39551 on aspartame-induced hypothalamic and adrenal gene expression
Source: PLoS One. 2018 Mar 21;13(3):e0194416. doi: 10.1371/journal.pone.0194416 (PMC5862471; doi:10.1371/journal.pone.0194416)
Supplement: S2 Table — (PDF) [file pone.0194416.s002.pdf]

S2 Table: Differential changes in hypothalamic gene expression in ASP+ CGP treated mice relative to ASP diet mice (≥±1.4 fold change, p< 0.05).

Gene Symbol	Gene name	RefSeq	P-value	Fold change
Abcb1b	ATP-binding cassette, sub-family B (MDR TAP), member 1B	NM_011075	2.97E-09	4.36
Abhd2	abhydrolase domain containing 2	NM_018811	2.33E-07	1.59
Acaa2	acetyl-Coenzyme A acyltransferase 2	NM_177470	2.03E-06	1.51
Acacb	Acetyl-Coenzyme A carboxylase bet	NM_133904	1.27E-06	1.41
Acsf2	Acyl-CoA synthetase family member 2	NM_153807	7.25E-05	1.40
Acsl1	acyl-CoA synthetase long-chain family member 1	NM_007981	8.78E-09	1.58
Acsm3	acyl-CoA synthetase medium-chain family member 3	NM_016870	7.14E-05	1.59
Adh1	Alcohol dehydrogenase 1 (class I)	NM_007409	1.02E-11	11.59
Adig	adipogenin	NM_145635	4.94E-08	2.27
Adipoq	adiponectin, C1Q and collagen domain containing	NM_009605	6.40E-09	5.12
Aebp1	AE binding protein 1	NM_001291857	7.61E-08	1.47
Agpat2	1-acylglycerol-3-phosphate O-acyltransferase 2	NM_026212	1.30E-08	2.72
Agtr1a	angiotensin II receptor, type 1a	NM_177322	7.19E-10	5.01
Akr1b7	Aldo-keto reductase family 1, member B7	NM_009731	4.03E-12	32.76
Akr1b8	aldo-keto reductase family 1, member B8	NM_008012	6.80E-10	3.00
Akr1cl	aldo-keto reductase family 1, member C-like	NM_027582	1.12E-07	6.01
Alas1	aminolevulinic acid synthase 1	NM_001291835	4.07E-07	1.57
Alb	albumin	NM_009654	4.34E-05	1.78
Amy2a5	Amylase 2a5	NM_001042711	9.15E-15	14.12
Antxr2	anthrax toxin receptor 2	NM_133738	1.99E-06	1.67
Aoc3	amine oxidase, copper containing 3	NM_009675	4.46E-07	1.58
Aqp1	aquaporin 1	NM_007472	0.001199	1.44
Aqp7	aquaporin 7	NM_007473	4.30E-05	2.14
Ascl1	achaete-scute complex homolog 1 (Drosophila)	NM_008553	0.000337	1.50
Bgn	biglycan	NM_007542	3.49E-08	1.99
C1rb	complement component 1, r subcomponent B	NM_001113356	7.30E-06	1.93
C2	Complement component 2 (within H-2S)	NM_013484	0.000329	1.42
C3	complement component 3	NM_009778	5.00E-08	1.94
C7	complement component 7	NM_001243837	7.29E-07	1.94
Car3	Carbonic anhydrase 3	NM_007606	7.29E-12	19.70
Car5b	carbonic anhydrase 5b, mitochondrial	NM_181315	4.85E-06	1.69
Casp8	caspase 8	NM_001080126	5.52E-06	1.43
Cbln3	Cerebellin 3 precursor protein	NM_019820	0.000136	-1.80
Ccnd2	cyclin D2	NM_009829	7.24E-08	1.84
Cd151	CD151 antigen	NM_001111049	4.67E-06	1.48
Cd36	CD36 antigen	NM_001159555	8.70E-10	7.26
Cd74	CD74 antigen	NM_001042605	3.54E-10	3.35
Cel	carboxyl ester lipase	NM_009885	4.44E-06	2.07
Cela2a	chymotrypsin-like elastase family, member 2A	NM_007919	1.34E-05	2.02
Cela3b	chymotrypsin-like elastase family, member 3B	NM_026419	0.000102	2.20
Ces1d	carboxylesterase 1D	NM_053200	1.07E-07	3.84
Ces1f	carboxylesterase 1F	NM_144930	5.34E-05	1.42

S2 Table: Differential changes in hypothalamic gene expression in ASP+ CGP treated mice relative to ASP diet mice (≥±1.4 fold change, p< 0.05).

Gene Symbol	Gene name	RefSeq	P-value	Fold change
Cfd	complement factor D (adipsin)	NM_001291915	1.75E-07	2.35
Cfh	complement component factor h	NM_009888	1.89E-05	1.77
Cga	Glycoprotein hormones, alpha subunit	NM_009889	4.65E-09	9.72
Cidea	cell death-inducing DNA fragmentation factor, α subunit-like	NM_007702	6.39E-07	2.06
Cidec	cell death-inducing DFFA-like effector c	NM_178373	3.17E-11	6.09
Cldn9	claudin 9	NM_020293	7.03E-05	1.51
Clic1	chloride intracellular channel 1	NM_033444	6.80E-05	1.49
Clps	colipase, pancreatic	NM_025469	8.01E-10	3.37
Cobll1	Cobl-like 1	NM_027225	1.05E-06	1.62
Cox8b	cytochrome c oxidase subunit VIIIb	NM_007751	2.25E-10	3.76
Cpa1	carboxypeptidase A1, pancreatic	NM_025350	2.70E-10	6.19
Cpa2	carboxypeptidase A2, pancreatic	NM_001024698	2.38E-05	1.63
Cpb1	carboxypeptidase B1 (tissue)	NM_029706	1.36E-10	5.28
Cpt1b	carnitine palmitoyltransferase 1b, muscle	NM_009948	9.09E-06	1.79
Creb3l2	cAMP responsive element binding protein 3-like 2	NM_178661	8.11E-07	1.44
Ctrb1	Chymotrypsinogen B1	NM_025583	3.12E-10	8.64
Ctrl	chymotrypsin-like	NM_023182	2.17E-07	2.04
Ctsh	cathepsin H	NM_007801	4.56E-05	1.49
Cxcr4	chemokine (C-X-C motif) receptor 4	NM_009911	2.54E-05	1.47
Cybb	cytochrome b-245, beta polypeptide	NM_007807	9.01E-06	1.47
Cyp11a1	Cytochrome P450, family 11, subfamily a, polypeptide 1	NM_019779	2.27E-11	27.43
Cyp11b1	Cytochrome P450, family 11, subfamily b, polypeptide 1	NM_001033229	1.62E-13	60.83
Cyp11b2	cytochrome P450, family 11, subfamily b, polypeptide 2	NM_009991	5.25E-07	2.79
Cyp1b1	cytochrome P450, family 1, subfamily b, polypeptide 1	NM_009994	1.13E-05	1.52
Cyp21a1	Cytochrome P450, family 21, subfamily a, polypeptide 1	NM_009995	9.49E-12	53.13
Cyp2e1	Cytochrome P450, family 2, subfamily e, polypeptide 1	NM_021282	1.04E-11	13.27
Cyp2f2	cytochrome P450, family 2, subfamily f, polypeptide 2	NM_007817	8.14E-09	2.49
Dab2	disabled 2, mitogen-responsive phosphoprotein	NM_001008702	1.12E-08	1.97
Dap	Death-associated protein	NM_146057	1.90E-05	1.40
Dapl1	death associated protein-like 1	NM_029723	1.72E-05	1.67
Dbh	Dopamine beta hydroxylase	NM_138942	6.28E-10	7.14
Dcn	decorin	NM_001190451	3.72E-07	1.77
Dhcr7	7-dehydrocholesterol reductase	NM_007856	3.67E-07	1.52
Dmbt1	deleted in malignant brain tumors 1	NM_007769	2.62E-05	1.57
Dpep1	dipeptidase 1 (renal)	NM_007876	5.98E-07	2.49
Ecm1	extracellular matrix protein 1	NM_001252653	8.91E-09	2.42
Ehhadh	enoyl-Coenzyme A, hydratase 3-hydroxyacyl dehydrogenase	NM_023737	0.000296	1.47
Elovl3	elongation of very long chain fatty acids	NM_007703	8.92E-05	1.45
Emcn	endomucin	NM_001163522	0.00072	1.47
Emp3	epithelial membrane protein 3	NM_001146346	4.64E-09	4.41
Epcam	epithelial cell adhesion molecule	NM_008532	1.80E-07	2.67
Ephx1	Epoxide hydrolase 1, microsomal	NM_010145	1.60E-05	1.41
Ern1	endoplasmic reticulum (ER) to nucleus signalling 1	NM_023913	1.14E-08	1.45

S2 Table: Differential changes in hypothalamic gene expression in ASP+ CGP treated mice relative to ASP diet mice (≥±1.4 fold change, p< 0.05).

Gene Symbol	Gene name	RefSeq	P-value	Fold change
Fabp4	Fatty acid binding protein 4, adipocyte	BC002148	1.24E-10	16.17
Fam129a	family with sequence similarity 129, member A	NM_022018	4.09E-06	1.87
Fdx1	ferredoxin 1	NM_007996	4.40E-08	2.32
Fdxr	ferredoxin reductase	NM_007997	5.86E-08	1.57
Fetub	fetuin beta	NM_001083904	7.61E-05	1.49
Figf	c-fos induced growth factor	NM_010216	4.73E-06	1.63
Fshb	follicle stimulating hormone beta	NM_008045	1.30E-08	3.21
Gabra6	Gamma-aminobutyric acid (GABA) A receptor, subunit alpha 6	NM_001099641	0.000193	-1.58
Gadd45b	Growth arrest and DNA-damage-inducible 45 beta	NM_008655	1.65E-06	1.42
Gata6	GATA binding protein 6	NM_010258	1.14E-05	1.84
Gh	Growth hormone	NM_008117	1.79E-14	8.34
Ghrhr	growth hormone releasing hormone receptor	NM_001003685	5.46E-07	1.88
Gp49a	glycoprotein 49 A	NM_001291892	0.00018	1.49
Gpc4	glypican 4	NM_008150	2.26E-07	1.68
Gpnmb	glycoprotein (transmembrane) nmb	NM_053110	7.64E-05	1.57
Greb1	gene regulated by estrogen in breast cancer protein	NM_001252071	2.56E-07	1.66
Gstm2	Glutathione S-transferase, mu 2	NM_008183	0.00083	1.42
H2-Aa	histocompatibility 2, class II antigen A, alpha	NM_010378	1.56E-09	3.55
H2-Ab1	histocompatibility 2, class II antigen A, beta 1	NM_207105	2.45E-08	2.85
H2-Eb1	histocompatibility 2, class II antigen E beta	NM_010382	0.000275	1.57
H2-K1	histocompatibility 2, K1, K region	NM_001001892	2.80E-07	1.78
H2-Q4	histocompatibility 2, Q region locus 4	NM_001143689	0.000429	1.43
H2-Q7	histocompatibility 2, Q region locus 7	NM_001198560	7.55E-05	1.53
Hadh	hydroxyacyl-Coenzyme A dehydrogenase	NM_008212	9.69E-07	1.45
Hao2	hydroxyacid oxidase 2	NM_019545	2.42E-06	2.15
Hmox1	heme oxygenase (decycling) 1	NM_010442	1.32E-06	1.63
Hoxb9	homeobox B9	NM_008270	3.25E-06	1.69
Hoxc8	homeobox C8	NM_010466	9.80E-07	1.73
Hp	haptoglobin	NM_017370	1.46E-05	1.72
Hsd3b1	Hydroxy-delta-5-steroid dehydrogenase,3 beta and steroid δ	NM_008293	3.43E-11	28.80
Hsd3b6	hydroxy-delta-5-steroid dehydrogenase,3 beta and steroid δ	NM_013821	1.49E-06	2.93
Hspb1	heat shock protein 1	NM_013560	5.90E-06	1.48
Hspg2	perlecan (heparan sulfate proteoglycan 2)	NM_008305	1.99E-07	1.51
Ifi27	interferon, alpha-inducible protein 27	NM_026790	3.22E-06	1.54
Ifi27l2a	interferon, alpha-inducible protein 27 like 2A	NM_029803	7.14E-05	1.72
Ifitm2	interferon induced transmembrane protein 2	NM_030694	1.38E-05	2.44
Ifitm3	interferon induced transmembrane protein 3	NM_025378	0.000115	1.60
Igfbp7	insulin-like growth factor binding protein 7	NM_001159518	4.36E-09	1.80
Il10rb	interleukin 10 receptor, beta	NM_008349	1.04E-05	1.54
Inmt	indolethylamine N-methyltransferase	NM_009349	5.05E-07	2.39
Isg20	interferon-stimulated protein	NM_001291220	3.97E-05	1.52
Kcnk3	potassium channel, subfamily K, member 3	NM_010608	1.02E-07	1.90
Kdr	kinase insert domain protein receptor	NM_010612	1.32E-06	1.48

S2 Table: Differential changes in hypothalamic gene expression in ASP+ CGP treated mice relative to ASP diet mice (≥±1.4 fold change, p< 0.05).

Gene Symbol	Gene name	RefSeq	P-value	Fold change
Klhdc8a	kelch domain containing 8A	NM_144810	1.48E-05	1.47
Lcn2	lipocalin 2	NM_008491	3.60E-07	1.86
Lgals1	lectin, galactose binding, soluble 1	NM_008495	1.82E-05	1.96
Lgals12	lectin, galactose binding, soluble 12	NM_019516	0.000133	1.43
Lhb	luteinizing hormone beta	NM_008497	6.22E-08	5.44
Lipe	lipase, hormone sensitive	NM_001039507	7.22E-07	1.62
Lpl	lipoprotein lipase	NM_008509	4.78E-11	3.45
Lrg1	leucine-rich alpha-2-glycoprotein 1	NM_029796	7.33E-06	1.74
Ly6d	Lymphocyte antigen 6 complex, locus D	NM_010742	1.79E-09	9.71
Lyz2	lysozyme 2	NM_017372	9.60E-05	1.78
Man2a1	mannosidase 2, alpha 1	NM_008549	9.91E-06	1.53
Mc2r	Melanocortin 2 receptor	NM_001271716	7.14E-09	13.38
Me1	malic enzyme 1, NADP(+)-dependent, cytosolic	NM_001198933	5.10E-09	1.71
Mgarp	Mitochondria localized glutamic acid rich protein	NM_026358	4.64E-12	10.62
Mmp19	matrix metallopeptidase 19	NM_001164197	0.000223	1.46
Mrap	melanocortin 2 receptor accessory protein	NM_029844	3.09E-10	4.68
Mup10	major urinary protein 10	NM_001122647	9.31E-08	2.72
Mup2	major urinary protein 2	NM_001045550	3.44E-07	3.00
Myo9a	Myosin IXa	NM_173018	8.66E-05	-1.44
Nid1	nidogen 1	NM_010917	7.01E-07	1.46
Npr1	natriuretic peptide receptor 1	NM_008727	7.76E-07	2.21
Npy	neuropeptide Y	NM_023456	1.20E-05	1.95
Nr0b2	nuclear receptor subfamily 0, group B, member 2	NM_011850	2.63E-05	2.12
Nr4a1	nuclear receptor subfamily 4, group A, member 1	NM_010444	0.000157	1.53
Nr5a1	nuclear receptor subfamily 5, group A, member 1	NM_139051	2.49E-07	1.64
Oacyl	O-acyltransferase like	NM_177028	4.47E-08	1.91
Ogn	osteoglycin	NM_008760	0.000204	1.52
Osgin1	oxidative stress induced growth inhibitor 1	NM_027950	0.000106	1.45
Pck1	phosphoenolpyruvate carboxykinase 1, cytosolic	NM_011044	8.28E-12	2.97
Pcm1	Pericentriolar material 1	NM_023662	3.89E-05	-1.50
Pcolce	procollagen C-endopeptidase enhancer protein	NM_008788	2.23E-09	3.55
Pdk4	pyruvate dehydrogenase kinase, isoenzyme 4	NM_013743	2.68E-07	1.93
Plin1	perilipin 1	NM_001113471	1.35E-07	2.69
Plin2	perilipin 2	NM_007408	4.96E-07	2.06
Plod2	procollagen lysine, 2-oxoglutarate 5-dioxygenase 2	NM_001142916	1.15E-05	1.67
Plp2	proteolipid protein 2	NM_019755	0.000351	1.57
Pnlip	pancreatic lipase	NM_026925	1.71E-06	2.00
Pnliprp1	pancreatic lipase related protein 1	NM_018874	2.62E-05	1.96
Pnmt	Phenylethanolamine-N-methyltransferase	NM_008890	1.11E-08	6.82
Pnpla2	patatin-like phospholipase domain containing 2	NM_001163689	4.55E-08	1.65
Pomc	pro-opiomelanocortin-alpha	NM_001278581	8.29E-13	5.98
Pon3	paraoxonase 3	NM_173006	1.42E-07	1.78
Prl	Prolactin	NM_001163530	3.87E-09	29.82

S2 Table: Differential changes in hypothalamic gene expression in ASP+ CGP treated mice relative to ASP diet mice (≥±1.4 fold change, p< 0.05).

Gene Symbol	*Gene name*	*RefSeq*	*P-value*	*Fold change*
Prss2	protease, serine 2	NM_009430	1.89E-06	1.79
Prss35	protease, serine 35	NM_178738	5.27E-09	5.17
Rab25	RAB25, member RAS oncogene family	NM_016899	2.93E-05	1.62
Rbm47	RNA binding motif protein 47	NM_001127382	4.75E-06	1.66
Rbp4	Retinol binding protein 4, plasma	NM_001159487	3.05E-05	1.41
Rbpms	RNA binding protein gene with multiple splicing	NM_001042674	1.45E-06	1.62
Rcn3	reticulocalbin 3, EF-hand calcium binding domain	NM_026555	0.000991	1.47
Reg1	regenerating islet-derived 1	NM_009042	1.22E-05	1.80
Retn	resistin	NM_001204959	6.63E-10	6.63
Rnase1	ribonuclease, RNase A family, 1 (pancreatic)	NM_011271	2.22E-06	1.75
S100a11	S100 calcium binding protein A11 (calgizzarin)	NM_016740	2.76E-05	1.55
S100a5	S100 calcium binding protein A5	NM_011312	3.08E-05	1.60
Scarb1	scavenger receptor class B, member 1	NM_001205082	1.46E-11	5.24
Serping1	serine (or cysteine) peptidase inhibitor, clade G, member 1	NM_009776	1.06E-08	1.80
Slc18a1	solute carrier family 18 (vesicular monoamine), member 1	NM_153054	8.92E-06	1.61
Slc1a5	solute carrier family 1 (neutral amino acid transporter)	NM_009201	7.68E-08	1.96
Slc25a24	solute carrier family 25 (mitochondrial carrier)	NM_172685	0.000366	1.59
Slc27a3	solute carrier family 27 (fatty acid transporter), member 3	NM_011988	8.96E-08	1.56
Slc43a3	solute carrier family 43, member 3	NM_021398	1.01E-06	2.28
Slc44a3	solute carrier family 44, member 3	NM_145394	9.60E-05	1.62
Slc6a4	solute carrier family 6 (serotonin)	NM_010484	1.57E-05	1.50
Soat1	sterol O-acyltransferase 1	NM_009230	1.35E-09	1.57
Sod3	superoxide dismutase 3, extracellular	NM_011435	6.62E-06	1.78
Srd5a2	Steroid 5 alpha-reductase 2	NM_053188	2.09E-10	10.15
Star	Steroidogenic acute regulatory protein	NM_011485	9.47E-13	20.27
Steap1	six transmembrane epithelial antigen of the prostate 1	NM_027399	3.10E-05	1.68
Sycn	syncollin	NM_026716	2.32E-07	2.49
Tacr2	tachykinin receptor 2	NM_009314	2.91E-06	1.97
Tbx19	T-box 19	NM_032005	0.000447	1.45
Tbx20	T-box 20	NM_001205085	2.79E-05	2.19
Tbx3	T-box 3	NM_011535	1.61E-07	2.25
Tgfbr3	transforming growth factor, beta receptor III	NM_011578	3.45E-08	1.55
Th	tyrosine hydroxylase	NM_009377	9.42E-05	1.48
Timp1	tissue inhibitor of metalloproteinase 1	NM_001044384	5.89E-05	1.50
Tinagl1	tubulointerstitial nephritis antigen-like 1	NM_001168333	8.36E-06	1.55
Tmem140	Transmembrane protein 140	NM_197986	0.000147	1.41
Tmem159	transmembrane protein 159	NM_145586	2.93E-05	1.61
Tmem176a	transmembrane protein 176A	NM_001098271	0.000295	1.49
Tmem184a	transmembrane protein 184a	NM_001161548	5.86E-05	1.85
Tmem45b	transmembrane protein 45b	NM_144936	1.26E-06	1.95
Tnfrsf12a	tumor necrosis factor receptor superfamily, member 12a	NM_001161746	0.000103	1.43
Tns1	tensin 1	AK053112	1.11E-05	1.49
Trim25	tripartite motif-containing 25	NM_009546	7.46E-06	1.50

S2 Table: Differential changes in hypothalamic gene expression in ASP+ CGP treated mice relative to ASP diet mice (≥±1.4 fold change, p< 0.05).

Gene Symbol	*Gene name*	*RefSeq*	*P-value*	*Fold change*
Try5	trypsin 5	NM_001003405	0.000411	1.87
Tshb	thyroid stimulating hormone, beta subunit	NM_001165939	0.000155	1.63
Tspo	translocator protein	NM_009775	3.44E-06	2.00
Tubb6	tubulin, beta 6 class V	NM_026473	5.83E-05	1.63
Tusc5	tumor suppressor candidate 5	NM_177709	1.30E-05	1.84
Txnip	thioredoxin interacting protein	NM_001009935	8.65E-09	1.97
Ucp1	uncoupling protein 1 (mitochondrial, proton carrier)	NM_009463	3.06E-10	5.98
Unc13c	Unc-13 homolog C (C. elegans)	NM_001081153	1.12E-06	-1.55
Zfp36	zinc finger protein 36	NM_011756	0.000217	1.45
Zfp935	Zinc finger protein 935	NM_001136496	0.000875	-1.45
Zg16	zymogen granule protein 16	NM_026918	2.80E-08	4.09
