# Supplementary material for: Effect of developmental NMDAR antagonism with CGP 39551 on aspartame-induced hypothalamic and adrenal gene expression
Source: PLoS One. 2018 Mar 21;13(3):e0194416. doi: 10.1371/journal.pone.0194416 (PMC5862471; doi:10.1371/journal.pone.0194416)
Supplement: S3 Table — (PDF) [file pone.0194416.s003.pdf]

S3 Table: Differential changes in adrenal gene expression in ASP+CGP treated mice relative to ASP diet mice (≥±1.8 fold change, p <0.05).

Gene symbol	*Gene name*	*RefSeq*	*P-value*	*Fold Change*
Abat	4-aminobutyrate aminotransferase	NM_001170978	2.74E-08	2.09
Abca2	ATP-binding cassette, sub-family A (ABC1), member 2	NM_007379	1.48E-09	2.17
Abhd8	abhydrolase domain containing 8	NM_022419	1.88E-06	1.84
Ache	acetylcholinesterase	NM_001290010	1.35E-09	2.84
Ackr1	atypical chemokine receptor 1 (Duffy blood group)	NM_010045	2.16E-07	2.67
Acot7	acyl-CoA thioesterase 7	NM_001146057	4.80E-10	2.74
Acsl6	acyl-CoA synthetase long-chain family member 6	NM_001033597	7.09E-08	2.72
Actr3b	ARP3 actin-related protein 3B	NM_001004365	2.30E-06	2.05
Acvr1b	activin A receptor, type 1B	NM_007395	1.47E-05	1.99
Adam22	a disintegrin and metallopeptidase domain 22	NM_001007220	2.48E-08	3.07
Adam23	a disintegrin and metallopeptidase domain 23	NM_001177600	1.23E-08	2.09
Adap1	ArfGAP with dual PH domains 1	NM_172723	1.56E-08	3.53
Adar	adenosine deaminase, RNA-specific	NM_001038587	3.20E-07	1.85
Adcy1	adenylate cyclase 1	NM_009622	2.82E-05	1.81
Adcy2	adenylate cyclase 2	NM_153534	7.07E-07	4.37
Adcy5	adenylate cyclase 5	NM_001012765	5.97E-08	2.88
Adcy8	adenylate cyclase 8	NM_001291903	4.76E-07	2.71
Adcyap1	adenylate cyclase activating polypeptide 1	NM_009625	5.93E-07	3.81
Add2	adducin 2 (beta)	NM_001271860	9.28E-05	1.88
Add3	adducin 3 (gamma)	NM_001164099	2.88E-07	1.81
Aff2	AF4 FMR2 family, member 2	NM_008032	5.20E-06	2.24
Aff3	AF4 FMR2 family, member 3	NM_001290814	3.81E-07	2.07
Agap2	ArfGAP with GTPase domain, ankyrin repeat and PH domain 2	NM_001033263	4.09E-11	5.01
Agbl4	ATP GTP binding protein-like 4	NM_001048189	2.25E-05	2.13
Agt	angiotensinogen (serpin peptidase inhibitor, member 8)	NM_007428	2.46E-11	6.86
Agtpbp1	ATP GTP binding protein 1	NM_001048008	1.28E-07	2.40
Ahi1	Abelson helper integration site 1	NM_026203	2.70E-09	3.76
Ajap1	adherens junction associated protein 1	NM_001099299	4.32E-08	5.60
Ak4	adenylate kinase 4	NM_001177602	3.13E-07	1.94
Ak5	adenylate kinase 5	NM_001081277	8.58E-07	2.09
Akap6	A kinase (PRKA) anchor protein 6	NM_198111	2.10E-07	4.21
Aktip	thymoma viral proto-oncogene 1 interacting protein	NM_010241	1.75E-07	1.82
Aldoc	aldolase C, fructose-bisphosphate	NM_009657	4.54E-13	13.20
Amigo2	adhesion molecule with Ig like domain 2	NM_001164563	1.55E-05	2.11
Amph	amphiphysin	NM_001289546	2.38E-09	5.22
Ank2	ankyrin 2, brain	NM_001034168	2.26E-10	3.38
Ank3	ankyrin 3, epithelial	NM_009670	9.88E-10	4.06
Ankrd34a	ankyrin repeat domain 34A	NM_001024851	0.0002732	1.95
Ankrd34c	ankyrin repeat domain 34C	NM_207260	2.82E-06	2.39
Ankrd55	ankyrin repeat domain 55	NM_001168404	2.17E-08	1.96
Anks1b	ankyrin repeat and sterile alpha motif domain containing 1B	NM_001177397	2.60E-08	5.45
Anln	anillin, actin binding protein	NM_028390	8.55E-07	2.35
Ano4	anoctamin 4	NM_001277188	2.86E-06	2.09
Ap3m2	adaptor-related protein complex 3, mu 2 subunit	NM_001122820	7.91E-07	2.29
Apba1	amyloid beta (A4) precursor protein binding, family A, member 1	NM_177034	2.93E-08	3.88
Apba2	amyloid beta (A4) precursor protein-binding, family A	NM_001291166	1.47E-07	3.92
Apbb1	amyloid beta (A4) precursor protein-binding, family B, member	NM_001253885	4.78E-08	2.48
Apc2	adenomatosis polyposis coli 2	NM_011789	1.59E-06	2.12
Aplp1	amyloid beta (A4) precursor-like protein 1	NM_007467	3.94E-11	4.59
Apod	apolipoprotein D	NM_007470	1.08E-11	5.26

S3 Table: Differential changes in adrenal gene expression in ASP+CGP treated mice relative to ASP diet mice (≥±1.8 fold change, p <0.05).

Gene symbol	Gene name	RefSeq	P-value	Fold Change
App	amyloid beta (A4) precursor protein	NM_001198823	1.31E-10	2.20
Arhgap20	Rho GTPase activating protein 20	NM_175535	5.94E-08	3.98
Arhgap32	Rho GTPase activating protein 32	NM_001195632	5.73E-07	2.18
Arhgap36	Rho GTPase activating protein 36	NM_001081123	3.23E-07	3.96
Arhgap44	Rho GTPase activating protein 44	NM_001099288	2.80E-09	2.12
Arhgef4	Rho guanine nucleotide exchange factor (GEF) 4	NM_183019	1.05E-08	3.35
Arhgef9	CDC42 guanine nucleotide exchange factor (GEF) 9	NM_001033329	6.65E-10	5.43
Arpp21	cyclic AMP-regulated phosphoprotein, 21	NM_001177616	3.35E-09	3.56
Arrb1	arrestin, beta 1	NM_177231	1.34E-07	2.02
Arxes1	adipocyte-related X-chromosome expressed sequence 1	NM_029541	1.66E-06	2.47
Arxes2	adipocyte-related X-chromosome expressed sequence 2	NM_029823	3.59E-06	1.90
Asic1	acid-sensing (proton-gated) ion channel 1	NM_001289791	8.03E-07	2.10
Asic2	acid-sensing (proton-gated) ion channel 2	NM_001034013	1.04E-08	2.59
Asphd2	aspartate beta-hydroxylase domain containing 2	NM_028386	1.98E-06	2.81
Asrgl1	asparaginase like 1	NM_025610	6.78E-07	1.86
Astn1	astrotactin 1	NM_001205204	6.57E-10	2.90
Atcay	ataxia, cerebellar, Cayman type homolog (human)	NM_178662	4.36E-08	3.70
Atl1	atlastin GTPase 1	NM_178628	2.89E-09	4.34
Atp13a4	ATPase type 13A4	NM_001164612	2.81E-05	1.99
Atp1a2	ATPase, Na+ K+ transporting, alpha 2 polypeptide	NM_178405	3.18E-08	2.34
Atp1a3	ATPase, Na+ K+ transporting, alpha 3 polypeptide	NM_001290469	1.25E-11	7.11
Atp1b1	ATPase, Na+ K+ transporting, beta 1 polypeptide	NM_009721	6.82E-10	3.86
Atp1b2	ATPase, Na+ K+ transporting, beta 2 polypeptide	NM_013415	2.39E-08	3.30
Atp2b1	ATPase, Ca++ transporting, plasma membrane 1	NM_026482	5.34E-08	2.15
Atp2b2	ATPase, Ca++ transporting, plasma membrane 2	NM_001036684	5.66E-10	4.23
Atp6ap2	ATPase, H+ transporting, lysosomal accessory protein 2	NM_027439	1.04E-05	1.88
Atp6v0a1	ATPase, H+ transporting, lysosomal V0 subunit A1	NM_001243049	8.64E-09	1.94
Atp6v1b2	ATPase, H+ transporting, lysosomal V1 subunit B2	NM_007509	4.02E-09	1.91
Atp6v1c1	ATPase, H+ transporting, lysosomal V1 subunit C1	NM_025494	1.98E-08	2.23
Atp6v1g2	ATPase, H+ transporting, lysosomal V1 subunit G2	NM_023179	1.56E-12	7.11
Atp8a1	ATPase, aminophospholipid transporter (APLT), class I, type 8	NM_001038999	6.76E-09	2.93
Atp8a2	ATPase, aminophospholipid transporter-like, class I, type 8A2	NM_015803	2.50E-07	2.20
Atp9a	ATPase, class II, type 9A	NM_001289445	5.68E-07	1.90
Atrn	attractin	NM_009730	6.53E-09	1.84
Atxn1	ataxin 1	NM_001199304	1.92E-07	2.22
Atxn7l3	ataxin 7-like 3	NM_001098836	1.05E-06	2.05
Avp	arginine vasopressin	NM_009732	6.09E-05	1.96
B3galt5	UDP-Gal:betaGlcNAc beta 1,3-galactosyltransferase	NM_001122993	1.41E-06	3.03
B3gat2	beta-1,3-glucuronyltransferase 2 (glucuronosyltransferase S)	NM_172124	8.39E-06	2.06
B4galnt4	beta-1,4-N-acetyl-galactosaminyl transferase 4	NM_177897	7.00E-07	2.39
B4galt6	UDP-Gal:betaGlcNAc beta 1,4-galactosyltransferase	NM_019737	4.17E-09	2.64
Baalc	brain and acute leukemia, cytoplasmic	NM_080640	1.99E-06	1.98
Bai1	brain-specific angiogenesis inhibitor 1	NM_174991	1.79E-09	3.67
Bai3	brain-specific angiogenesis inhibitor 3	NM_175642	3.04E-09	7.00
Baiap3	BAI1-associated protein 3	NM_001163270	1.18E-05	2.06
Basp1	brain abundant, membrane attached signal protein 1	NM_027395	2.63E-07	2.71
Bcas1	breast carcinoma amplified sequence 1	NM_001164369	3.77E-09	4.50
Bcat1	branched chain aminotransferase 1, cytosolic	NM_001024468	6.57E-09	4.22
Begain	brain-enriched guanylate kinase-associated	NM_001163175	2.23E-07	2.58
Bend6	BEN domain containing 6	NM_177235	5.03E-10	12.11

S3 Table: Differential changes in adrenal gene expression in ASP+CGP treated mice relative to ASP diet mice (≥±1.8 fold change, p <0.05).

Gene symbol	Gene name	RefSeq	P-value	Fold Change
Bex1	brain expressed gene 1	NM_009052	1.93E-06	4.25
Bex2	brain expressed X-linked 2	NM_009749	1.36E-06	2.17
Bglap	bone gamma carboxyglutamate protein	NM_001037939	0.0004952	-2.14
Bin1	bridging integrator 1	NM_001083334	3.53E-09	2.07
Brinp1	bone morphogenetic protein retinoic acid neural specific 1	NM_019967	1.04E-08	3.18
Brinp3	bone morphogenetic protein retinoic acid neural specific 3	NM_001145807	2.50E-06	2.71
Brsk1	BR serine threonine kinase 1	NM_001003920	9.18E-09	4.62
Brsk2	BR serine threonine kinase 2	NM_001009929	3.01E-08	4.06
Btbd3	BTB (POZ) domain containing 3	NM_001025431	4.73E-09	1.99
Btnl5-ps	butyrophilin-like 5, pseudogene	NR_004051	6.45E-06	-2.95
C1ql3	C1q-like 3	NM_153155	1.60E-06	1.95
C1qtnf4	C1q and tumor necrosis factor related protein 4	NM_026161	0.0001288	1.85
Cabp7	calcium binding protein 7	NM_138948	2.97E-05	2.50
Cacna1a	calcium channel, voltage-dependent, P Q type, alpha 1A	NM_001252059	4.83E-08	2.39
Cacna1b	calcium channel, voltage-dependent, N type, alpha 1B subunit	NM_001042528	8.40E-08	2.60
Cacna1e	calcium channel, voltage-dependent, R type, alpha 1E subunit	NM_009782	6.87E-09	3.39
Cacna1g	calcium channel, voltage-dependent, T type, alpha 1G subunit	NM_001112813	2.00E-07	2.96
Cacna1i	calcium channel, voltage-dependent, alpha 1I subunit	NM_001044308	5.88E-05	1.85
Cacna2d2	calcium channel, voltage-dependent, alpha 2 delta subunit 2	NM_001174047	1.35E-09	3.03
Cacna2d3	calcium channel, voltage-dependent, alpha 2 delta subunit 3	NM_009785	2.04E-07	2.28
Cacnb1	calcium channel, voltage-dependent, beta 1 subunit	NM_001159319	2.07E-07	2.01
Cacnb4	calcium channel, voltage-dependent, beta 4 subunit	NM_001037099	7.78E-09	5.30
Cacng3	calcium channel, voltage-dependent, gamma subunit 3	NM_019430	5.01E-08	5.81
Cacng4	calcium channel, voltage-dependent, gamma subunit 4	NM_019431	9.74E-07	2.33
Cacng7	calcium channel, voltage-dependent, gamma subunit 7	NM_133189	5.50E-09	2.33
Cadm2	cell adhesion molecule 2	NM_001145977	3.13E-11	7.07
Cadm3	cell adhesion molecule 3	NM_053199	8.30E-09	5.36
Cadm4	cell adhesion molecule 4	NM_153112	9.24E-06	2.27
Cadps	Ca2+-dependent secretion activator	NM_001042617	3.80E-08	4.62
Calb1	calbindin 1	NM_009788	6.37E-10	11.20
Calb2	calbindin 2	NM_007586	1.94E-10	26.70
Calcr	calcitonin receptor	NM_001042725	2.28E-06	1.85
Caly	calcyon neuron-specific vesicular protein	NM_001190385	6.21E-09	2.97
Camk1g	calcium calmodulin-dependent protein kinase I gamma	NM_144817	6.13E-08	4.84
Camk2a	calcium calmodulin-dependent protein kinase II alpha	NM_001286809	2.00E-11	16.26
Camk2b	calcium calmodulin-dependent protein kinase II, beta	XM_006514478	6.87E-10	3.21
Camk2g	calcium calmodulin-dependent protein kinase II gamma	NM_001039138	2.28E-07	2.22
Camk2n1	calcium calmodulin-dependent protein kinase II inhibitor 1	NM_025451	6.58E-08	2.33
Camkk1	calcium calmodulin-dependent protein kinase kinase 1, alpha	NM_018883	1.50E-05	1.89
Camkv	CaM kinase-like vesicle-associated	NM_145621	9.68E-09	4.19
Camta1	calmodulin binding transcription activator 1	NM_001081557	1.34E-12	9.47
Cand1	cullin associated and neddylation disassociated 1	NM_027994	2.96E-06	1.89
Cap2	CAP, adenylate cyclase-associated protein, 2 (yeast)	NM_026056	3.74E-08	4.82
Car10	carbonic anhydrase 10	NM_028296	1.55E-08	5.10
Car14	carbonic anhydrase 14	NM_011797	1.33E-06	2.04
Cbln1	cerebellin 1 precursor protein	NM_019626	1.63E-08	3.77
Cbln2	cerebellin 2 precursor protein	NM_172633	8.14E-08	1.94
Cbx6	chromobox 6	NM_028763	1.07E-07	1.89
Ccdc106	coiled-coil domain containing 106	NM_001290429	1.05E-06	2.08
Ccdc85a	coiled-coil domain containing 85A	NM_001166661	9.77E-08	2.84
Ccdc92	coiled-coil domain containing 92	NM_144819	2.14E-07	2.16

S3 Table: Differential changes in adrenal gene expression in ASP+CGP treated mice relative to ASP diet mice (≥±1.8 fold change, p <0.05).

Gene symbol	Gene name	RefSeq	P-value	Fold Change
Cck	cholecystokinin	NM_001284508	1.35E-07	2.80
Cdh13	cadherin 13	NM_019707	4.31E-08	2.69
Cdh8	cadherin 8	NM_001285913	2.83E-06	3.21
Cdk14	cyclin-dependent kinase 14	NM_011074	4.53E-06	2.11
Cdk5r1	cyclin-dependent kinase 5, regulatory subunit 1 (p35)	NM_009871	1.05E-05	3.23
Cdkl5	cyclin-dependent kinase-like 5	NM_001024624	5.87E-06	2.08
Cds1	CDP-diacylglycerol synthase 1	NM_173370	6.73E-06	2.13
Cds2	CDP-diacylglycerol synthase (phosphatidate cytidylyltransferase)	NM_001291039	1.48E-09	1.88
Celf2	CUGBP, Elav-like family member 2	NM_001110231	3.90E-08	2.44
Celf4	CUGBP, Elav-like family member 4	NM_001146292	2.50E-10	4.66
Celf5	CUGBP, Elav-like family member 5	NM_176954	3.79E-05	1.94
Celsr2	cadherin, EGF LAG seven-pass G-type receptor 2	NM_001004177	5.13E-07	2.89
Cend1	cell cycle exit and neuronal differentiation 1	NM_021316	6.45E-11	6.19
Cers6	ceramide synthase 6	NM_172856	3.97E-07	2.16
Chd3os	chromodomain helicase DNA binding protein 3	NR_027827	1.49E-05	2.15
Chd5	chromodomain helicase DNA binding protein 5	NM_001081376	9.55E-07	2.15
Chl1	cell adhesion molecule with homology to L1CAM	NM_007697	3.98E-06	2.70
Chn1	chimerin 1	NM_001113246	3.46E-10	5.74
Chn2	chimerin 2	NM_023543	6.08E-09	2.04
Chrm2	cholinergic receptor, muscarinic 2, cardiac	NM_203491	5.71E-07	5.04
Chrm3	cholinergic receptor, muscarinic 3, cardiac	NM_033269	2.63E-06	1.96
Chrna4	cholinergic receptor, nicotinic, alpha polypeptide 4	NM_015730	1.09E-06	2.19
Chrnb2	cholinergic receptor, nicotinic, beta polypeptide 2 (neuronal)	NM_009602	2.36E-08	2.57
Chst1	carbohydrate (keratan sulfate Gal-6) sulfotransferase 1	NM_023850	1.76E-07	2.24
Ciart	circadian associated repressor of transcription	NM_001033302	8.24E-06	3.34
Cit	citron	NM_007708	1.06E-07	2.93
Ckb	creatine kinase, brain	NM_021273	4.00E-09	3.07
Ckmt1	creatine kinase, mitochondrial 1, ubiquitous	NM_009897	2.61E-09	3.84
Clcn6	chloride channel 6	NM_011929	4.23E-09	2.80
Cldn10	claudin 10	NM_001160099	3.74E-07	3.54
Cldn11	claudin 11	NM_008770	1.66E-08	3.93
Clip3	CAP-GLY domain containing linker protein 3	NM_001081114	4.84E-09	3.42
Clip4	CAP-GLY domain containing linker protein family, member 4	NM_001271483	1.86E-06	2.00
Clmn	calmin	NM_001040682	3.56E-06	2.31
Clstn1	calsyntenin 1	NM_001290989	9.43E-08	2.02
Clstn2	calsyntenin 2	NM_022319	6.01E-08	2.64
Clstn3	calsyntenin 3	NM_153508	6.89E-09	3.74
Clvs1	clavesin 1	NM_028940	1.51E-08	3.13
Clvs2	clavesin 2	NM_175448	7.05E-08	4.17
Cmas	cytidine monophospho-N-acetylneuraminic acid synthetase	NM_009908	3.11E-08	2.03
Cmtm5	CKLF-like MARVEL transmembrane domain containing 5	NM_026066	5.73E-09	6.63
Cnih2	cornichon homolog 2 (Drosophila)	NM_009920	3.18E-11	6.23
Cnksr2	connector enhancer of kinase suppressor of Ras 2	NM_177751	9.07E-06	1.88
Cnnm1	cyclin M1	NM_031396	1.40E-08	5.46
Cnp	2,3-cyclic nucleotide 3 phosphodiesterase	NM_001146318	4.57E-10	3.14
Cntn1	contactin 1	NM_001159647	1.19E-09	9.92
Cntn2	contactin 2	NM_177129	2.80E-07	2.97
Cntn5	contactin 5	NM_001033359	3.20E-07	1.92
Cntnap1	contactin associated protein-like 1	NM_016782	1.66E-08	4.60
Cntnap2	contactin associated protein-like 2	NM_001004357	1.17E-11	3.69
Cntnap4	contactin associated protein-like 4	NM_130457	0.000163	2.05

S3 Table: Differential changes in adrenal gene expression in ASP+CGP treated mice relative to ASP diet mice (≥±1.8 fold change, p <0.05).

Gene symbol	Gene name	RefSeq	P-value	Fold Change
Coro2b	coronin, actin binding protein, 2B	NM_175484	1.75E-06	1.92
Cox8c	cytochrome c oxidase subunit VIIIc	NM_001039049	0.0001352	-1.81
Cpeb3	cytoplasmic polyadenylation element binding protein 3	NM_001290826	1.73E-07	2.93
Cplx1	complexin 1	NM_007756	2.64E-09	8.59
Cpne4	copine IV	NM_028719	7.91E-09	2.15
Cpne5	copine V	NM_153166	2.97E-07	2.85
Cpne6	copine VI	NM_001136057	4.44E-09	5.20
Cpne9	copine family member IX	NM_170673	3.12E-08	2.21
Crmp1	collapsin response mediator protein 1	NM_001136058	6.77E-10	2.01
Cry2	cryptochrome 2 (photolyase-like)	NM_009963	1.64E-08	2.02
Cryab	crystallin, alpha B	NM_001289784	4.42E-07	2.09
Csdc2	cold shock domain containing C2, RNA binding	NM_145473	1.53E-07	1.92
Csmd3	CUB and Sushi multiple domains 3	NM_001081391	3.66E-07	3.28
Cspg5	chondroitin sulfate proteoglycan 5	NM_001166273	1.66E-10	3.48
Csrnp3	cysteine-serine-rich nuclear protein 3	NM_001290665	1.63E-07	3.29
Ctnnd2	catenin (cadherin associated protein), delta 2	NM_008729	2.42E-11	4.32
Ctrc	chymotrypsin C (caldecrin)	NM_001033875	7.64E-06	-1.96
Ctxn1	cortexin 1	NM_183315	3.54E-11	3.49
Ctxn2	cortexin 2	NM_001162934	7.31E-06	2.79
Cyp2b23	cytochrome P450, family 2, subfamily b, polypeptide 23	NM_001081148	6.01E-05	-2.10
Cyp2j9	cytochrome P450, family 2, subfamily j, polypeptide 9	NM_028979	7.49E-08	3.04
Cyp3a11	cytochrome P450, family 3, subfamily a, polypeptide 11	NM_007818	8.04E-05	2.35
Cyp46a1	cytochrome P450, family 46, subfamily a, polypeptide 1	NM_010010	4.91E-09	3.20
Cyp4f15	cytochrome P450, family 4, subfamily f, polypeptide 15	NM_134127	8.91E-06	2.00
Dab1	disabled 1	NM_010014	4.27E-06	2.46
Dagla	diacylglycerol lipase, alpha	NM_198114	3.21E-06	1.99
Dbndd2	dysbindin (dystrobrevin binding protein 1) domain containing	NM_001048227	1.48E-06	2.16
Dcc	deleted in colorectal carcinoma	NM_007831	7.14E-06	1.96
Dclk1	doublecortin-like kinase 1	NM_001195538	9.09E-11	9.36
Dclk2	doublecortin-like kinase 2	NM_001195498	5.66E-06	1.91
Dcun1d4	DCN1, defective in cullin neddylation 1, domain containing 4	NM_001190733	7.49E-07	1.98
Ddah1	dimethylarginine dimethylaminohydrolase 1	NM_026993	9.71E-11	9.71
Ddhd1	DDHD domain containing 1	NM_001039106	6.55E-08	2.03
Ddn	dendrin	NM_001013741	3.22E-08	4.51
Ddx25	DEAD (Asp-Glu-Ala-Asp) box polypeptide 25	NM_013932	5.76E-06	2.06
Dennd6b	DENN MADD domain containing 6B	NM_027081	1.49E-07	2.07
Dgkb	diacylglycerol kinase, beta	AK032509	5.51E-09	2.16
Dgkg	diacylglycerol kinase, gamma	NM_138650	1.01E-08	2.32
Dgki	diacylglycerol kinase, iota	NM_001081206	1.32E-06	2.24
Dgkk	diacylglycerol kinase kappa	NM_177914	1.07E-07	2.40
Disp2	dispatched homolog 2 (Drosophila)	NM_170593	6.46E-09	2.26
Dlg4	discs, large homolog 4 (Drosophila)	NM_001109752	2.90E-10	3.64
Dlgap1	discs, large (Drosophila) homolog-associated protein 1	NM_001128180	2.18E-07	1.90
Dlx1	distal-less homeobox 1	NM_010053	4.11E-07	2.33
Dmtn	dematin actin binding protein	NM_001252662	1.07E-08	2.17
Dnajc18	DnaJ (Hsp40) homolog, subfamily C, member 18	NM_029669	6.27E-06	2.22
Dnajc27	DnaJ (Hsp40) homolog, subfamily C, member 27	NM_153082	1.11E-08	2.79
Dnajc6	DnaJ (Hsp40) homolog, subfamily C, member 6	NM_001164583	2.83E-09	4.99
Dner	delta notch-like EGF-related receptor	NM_152915	1.56E-08	6.15
Dnm1	dynamin 1	XM_006497658	3.70E-10	4.72
Dnm3	dynamin 3	NM_001038619	4.13E-07	3.01

S3 Table: Differential changes in adrenal gene expression in ASP+CGP treated mice relative to ASP diet mice (≥±1.8 fold change, p <0.05).

Gene symbol	Gene name	RefSeq	P-value	Fold Change
Dock3	dedicator of cyto-kinesis 3	NM_153413	1.34E-09	5.10
Dock9	dedicator of cytokinesis 9	NM_001081039	1.32E-06	1.88
Dok5	docking protein 5	NM_001163686	1.37E-08	2.17
Dpp10	dipeptidylpeptidase 10	NM_199021	6.89E-07	3.64
Dpp6	dipeptidylpeptidase 6	NM_001136060	3.27E-10	4.25
Dpysl2	dihydropyrimidinase-like 2	NM_009955	5.44E-09	2.31
Dpysl5	dihydropyrimidinase-like 5	NM_023047	6.61E-08	2.48
Drd2	dopamine receptor D2	NM_010077	8.82E-06	1.93
Drp2	dystrophin related protein 2	NM_010078	4.44E-08	3.37
Dscam	Down syndrome cell adhesion molecule	NM_031174	5.16E-09	6.20
Dtna	dystrobrevin alpha	NM_001285807	3.69E-09	2.71
Dusp15	dual specificity phosphatase-like 15	NM_001159376	4.46E-07	2.80
Dusp26	dual specificity phosphatase 26 (putative)	NM_025869	3.49E-08	1.89
Dync1i1	dynein cytoplasmic 1 intermediate chain 1	NM_001191023	8.64E-07	3.01
Dzank1	double zinc ribbon and ankyrin repeat domains 1	NM_172859	2.55E-09	7.94
Dzip1	DAZ interacting protein 1	NM_025943	4.12E-08	3.06
Ecel1	endothelin converting enzyme-like 1	NM_001277925	2.50E-10	3.83
Edil3	EGF-like repeats and discoidin I-like domains 3	NM_001037987	7.76E-10	7.00
Eef1a2	eukaryotic translation elongation factor 1 alpha 2	NM_007906	8.46E-09	2.43
Efna3	ephrin A3	NM_010108	1.67E-07	4.00
Efnb3	ephrin B3	NM_007911	2.53E-05	2.34
Efr3b	EFR3 homolog B (S. cerevisiae)	NM_001082483	5.16E-12	3.99
Eif5a2	eukaryotic translation initiation factor 5A2	NM_177586	1.05E-07	2.32
Elavl2	ELAV (embryonic lethal, abnormal vision, Drosophila)-like 2	NM_001177883	7.54E-05	2.61
Elavl3	ELAV (embryonic lethal, abnormal vision, Drosophila)-like 3	NM_010487	1.10E-09	4.51
Elavl4	ELAV (embryonic lethal, abnormal vision, Drosophila)-like 4	NM_001163397	1.26E-08	3.65
Elmo1	engulfment and cell motility 1	NM_080288	1.09E-07	2.27
Elmod1	ELMO CED-12 domain containing 1	NM_177769	1.02E-10	6.69
Elovl4	elongation of very long chain fatty acids SUR4	NM_148941	2.40E-08	5.08
Endod1	endonuclease domain containing 1	NM_028013	3.23E-07	2.72
Eno2	enolase 2, gamma neuronal	NM_013509	7.17E-12	8.08
Enpp2	ectonucleotide pyrophosphatase phosphodiesterase 2	NM_001136077	3.02E-07	2.40
Entpd6	ectonucleoside triphosphate diphosphohydrolase 6	NM_172117	4.00E-06	2.05
Epdr1	ependymin related protein 1 (zebrafish)	NM_134065	1.31E-06	2.15
Epha4	Eph receptor A4	NM_007936	2.81E-07	2.46
Epha6	Eph receptor A6	NM_007938	2.22E-08	2.79
Ephb1	Eph receptor B1	NM_001168296	3.40E-06	2.03
Erbb4	v-erb-a erythroblastic leukemia viral oncogene homolog 4	NM_010154	1.33E-07	3.16
Ermn	ermin, ERM-like protein	NM_029972	0.0002269	2.30
Etnppl	ethanolamine phosphate phospholyase	NM_001163587	5.88E-08	4.75
Extl2	exostoses (multiple)-like 2	NM_001163514	3.18E-06	1.84
F3	coagulation factor III	NM_010171	1.02E-05	2.51
Fa2h	fatty acid 2-hydroxylase	NM_178086	9.73E-07	3.44
Fabp7	fatty acid binding protein 7, brain	NM_021272	7.95E-09	2.61
Faim2	Fas apoptotic inhibitory molecule 2	NM_001038658	7.62E-10	2.65
Fam107a	family with sequence similarity 107, member A	NM_183187	7.73E-10	6.05
Fam120c	family with sequence similarity 120, member C	NM_198105	3.19E-06	1.81
Fam131b	family with sequence similarity 131, member B	NM_001113327	2.33E-07	2.05
Fam135b	family with sequence similarity 135, member B	NM_177819	0.0076601	2.17
Fam155a	family with sequence similarity 155, member A	NM_173446	2.32E-06	2.29
Fam169a	family with sequence similarity 169, member A	NM_001100458	5.45E-06	2.28

S3 Table: Differential changes in adrenal gene expression in ASP+CGP treated mice relative to ASP diet mice (≥±1.8 fold change, p <0.05).

Gene symbol	Gene name	RefSeq	P-value	Fold Change
Fam171b	family with sequence similarity 171, member B	NM_175514	4.00E-09	4.74
Fam19a5	family with sequence similarity 19, member A5	NM_001252310	1.32E-07	1.96
Fam217b	family with sequence similarity 217, member B	NM_001081289	8.12E-06	1.91
Fam219a	family with sequence similarity 219, member A	NM_001159583	7.64E-08	2.21
Fam49b	family with sequence similarity 49, member B	NM_144846	2.62E-08	2.08
Fam63b	family with sequence similarity 63, member B	NM_172772	0.0002835	1.90
Fam65b	family with sequence similarity 65, member B	NM_001286100	1.46E-08	2.24
Fam73a	family with sequence similarity 73, member A	NM_001162375	6.29E-07	2.01
Fam81a	family with sequence similarity 81, member A	NM_029784	3.05E-06	2.28
Fam84a	family with sequence similarity 84, member A	NM_029007	3.14E-05	2.19
Far2	fatty acyl CoA reductase 2	NM_178797	4.44E-08	2.98
Fat3	FAT tumor suppressor homolog 3 (Drosophila)	NM_001080814	5.79E-08	3.72
Fbxl16	F-box and leucine-rich repeat protein 16	NM_001164225	2.06E-09	2.46
Fbxo44	F-box protein 44	NM_001161851	4.27E-07	2.19
Fcho1	FCH domain only 1	NM_028715	0.0001526	1.82
Fez1	fasciculation and elongation protein zeta 1 (zygin I)	NM_183171	7.30E-11	5.26
Fgf12	fibroblast growth factor 12	NM_183064	1.44E-08	5.64
Fgf13	fibroblast growth factor 13	NM_001290415	7.46E-06	2.41
Fgf14	fibroblast growth factor 14	NM_010201	2.31E-09	3.31
Fgfr3	fibroblast growth factor receptor 3	NM_001163215	1.06E-07	3.42
Fmn2	formin 2	NM_019445	3.73E-08	2.45
Fn3k	fructosamine 3 kinase	NM_001038699	5.31E-06	1.82
Fnbp1	formin binding protein 1	NM_001038700	8.34E-07	1.96
Fndc4	fibronectin type III domain containing 4	NM_022424	8.01E-06	2.09
Fndc9	fibronectin type III domain containing 9	NM_177075	5.51E-05	1.85
Frmpd3	FERM and PDZ domain containing 3	NM_177750	9.54E-08	2.77
Frrs1l	ferric-chelate reductase 1 like	NM_001142965	5.46E-09	4.89
Fry	furry homolog (Drosophila)	NM_172887	1.15E-09	3.25
Fscn1	fascin homolog 1, actin bundling protein	NM_007984	6.84E-07	2.13
Fsd1	fibronectin type 3 and SPRY domain-containing protein	NM_183178	1.77E-07	1.97
Fstl4	follistatin-like 4	NM_177059	0.0001103	1.88
Fto	fat mass and obesity associated	NM_011936	1.80E-08	1.81
Fuca2	fucosidase, alpha-L- 2, plasma	NM_025799	7.89E-08	1.90
Fxyd7	FXYD domain-containing ion transport regulator 7	NM_022007	7.09E-07	2.63
Gaa	glucosidase, alpha, acid	NM_001159324	4.94E-08	1.83
Gabbr1	gamma-aminobutyric acid (GABA) B receptor, 1	NM_019439	2.46E-11	6.47
Gabbr2	gamma-aminobutyric acid (GABA) B receptor, 2	NM_001081141	6.47E-12	9.58
Gabra1	gamma-aminobutyric acid (GABA) A receptor, subunit alpha 1	NM_010250	2.33E-12	39.65
Gabra2	gamma-aminobutyric acid (GABA) A receptor, subunit alpha 2	NM_008066	1.14E-07	2.61
Gabra3	gamma-aminobutyric acid (GABA) A receptor, subunit alpha 3	NM_008067	8.46E-08	2.53
Gabra4	gamma-aminobutyric acid (GABA) A receptor, subunit alpha 4	NM_010251	2.52E-08	5.47
Gabra5	gamma-aminobutyric acid (GABA) A receptor, subunit alpha 5	NM_176942	5.07E-11	10.82
Gabrb1	gamma-aminobutyric acid (GABA) A receptor, subunit beta 1	NM_008069	7.46E-11	6.11
Gabrb2	gamma-aminobutyric acid (GABA) A receptor, subunit beta 2	NM_008070	2.23E-10	13.75
Gabrb3	gamma-aminobutyric acid (GABA) A receptor, subunit beta 3	NM_001038701	7.30E-09	3.03
Gabre	gamma-aminobutyric acid (GABA) A receptor, subunit epsilon	NM_017369	4.59E-07	2.56
Gabrg1	gamma-aminobutyric acid (GABA) A receptor, subunit gamma 1	NM_010252	3.25E-09	8.44
Gabrg2	gamma-aminobutyric acid (GABA) A receptor, subunit gamma 2	NM_008073	1.76E-10	7.49
Gabrg3	gamma-aminobutyric acid (GABA) A receptor, subunit gamma 3	NM_008074	6.55E-08	4.15
Gabrq	gamma-aminobutyric acid (GABA) A receptor, subunit theta	NM_001290435	4.50E-09	6.42
Gad1	glutamate decarboxylase 1	NM_008077	3.52E-10	4.19

S3 Table: Differential changes in adrenal gene expression in ASP+CGP treated mice relative to ASP diet mice (≥±1.8 fold change, p <0.05).

Gene symbol	Gene name	RefSeq	P-value	Fold Change
Gad2	glutamic acid decarboxylase 2	NM_008078	2.65E-10	11.90
Gal3st3	galactose-3-O-sulfotransferase 3	NM_001024717	2.14E-05	1.84
Galnt13	UDP-N-acetyl-alpha-D-galactosamine:polypeptide N-acetylgalactos	NM_173030	6.87E-08	3.46
Galnt16	UDP-N-acetyl-alpha-D-galactosamine:polypeptide N-acetylgalactose	NM_001081421	4.36E-07	2.04
Galntl6	UDP-N-acetyl-alpha-D-galactosamine:polypeptide N-acetylgalactose	NM_175032	2.14E-07	1.87
Gap43	growth associated protein 43	NM_008083	7.85E-08	6.77
Gatm	glycine amidinotransferase	NM_025961	5.23E-09	6.92
Gatsl2	GATS protein-like 2	NM_030719	3.25E-09	1.99
Gda	guanine deaminase	NM_010266	1.27E-05	1.88
Gdap1	ganglioside-induced differentiation-associated-protein 1	NM_010267	6.48E-06	2.25
Gdap1l1	ganglioside-induced differentiation-associated protein 1-1	NM_144891	8.10E-08	2.69
Gdf1	growth differentiation factor 1	NM_001163282	4.70E-06	1.98
Gdpd1	glycerophosphodiester phosphodiesterase domain containing 1	NM_025638	1.74E-07	2.63
Gfap	glial fibrillary acidic protein	NM_010277	9.70E-09	3.94
Ggt7	gamma-glutamyltransferase 7	NM_144786	9.18E-08	2.34
Git1	G protein-coupled receptor kinase-interactor 1	NM_001004144	1.01E-07	1.85
Gja1	gap junction protein, alpha 1	NM_010288	5.32E-09	2.26
Gjb6	gap junction protein, beta 6	NM_001271663	8.83E-09	4.62
Gjc3	gap junction protein, gamma 3	NM_080450	3.76E-11	9.47
Glp1r	glucagon-like peptide 1 receptor	NM_021332	1.14E-05	1.86
Glra1	glycine receptor, alpha 1 subunit	NM_001290821	9.30E-07	3.75
Glra2	glycine receptor, alpha 2 subunit	NM_183427	2.15E-09	4.17
Glrb	glycine receptor, beta subunit	NM_001281969	2.03E-08	3.29
Gls	glutaminase	NM_001113383	6.08E-05	1.81
Gnai1	guanine nucleotide binding protein (G protein), alpha inhibiting	NM_010305	2.01E-07	2.76
Gnal	guanine nucleotide binding protein, alpha stimulating,	NM_010307	1.68E-08	7.69
Gnaz	guanine nucleotide binding protein, alpha z subunit	NM_010311	1.22E-07	2.06
Gng2	guanine nucleotide binding protein (G protein), gamma 2	NM_001038637	4.76E-09	2.29
Gng3	guanine nucleotide binding protein (G protein), gamma 3	NM_010316	1.14E-09	2.93
Gng7	guanine nucleotide binding protein (G protein), gamma 7	NM_001038655	2.01E-06	1.96
Gnl3l	guanine nucleotide binding protein-like 3 (nucleolar)-like	NM_001168600	3.31E-07	2.11
Gp1bb	glycoprotein Ib, beta polypeptide	NM_001001999	4.30E-10	2.74
Gpc5	glypican 5	NM_175500	0.0001091	2.08
Gpld1	glycosylphosphatidylinositol specific phospholipase D1	NM_008156	1.54E-08	3.30
Gpm6a	glycoprotein m6a	NM_001253754	2.65E-13	20.74
Gpm6b	glycoprotein m6b	NM_001177955	2.59E-11	8.33
Gpr101	G protein-coupled receptor 101	NM_001033360	4.04E-05	1.93
Gpr123	G protein-coupled receptor 123	NM_177469	2.41E-08	2.08
Gpr155	G protein-coupled receptor 155	NM_001190297	6.50E-07	1.86
Gpr158	G protein-coupled receptor 158	NM_001004761	2.40E-09	4.76
Gpr162	G protein-coupled receptor 162	NM_013533	1.66E-08	4.12
Gpr165	G protein-coupled receptor 165	NM_029536	1.83E-08	7.85
Gpr37	G protein-coupled receptor 37	NM_010338	2.53E-07	3.99
Gpr37l1	G protein-coupled receptor 37-like 1	NM_134438	1.03E-09	6.06
Gpr50	G-protein-coupled receptor 50	NM_010340	7.55E-07	3.57
Gpr52	G protein-coupled receptor 52	NM_001146330	4.12E-05	2.06
Gpr83	G protein-coupled receptor 83	NM_010287	1.54E-05	2.38
Gprasp2	G protein-coupled receptor associated sorting protein 2	NM_001163015	4.86E-08	3.08
Gria1	glutamate receptor, ionotropic, AMPA1 (alpha 1)	NM_001113325	9.87E-10	15.85
Gria2	glutamate receptor, ionotropic, AMPA2 (alpha 2)	NM_001039195	2.08E-08	2.86
Gria4	glutamate receptor, ionotropic, AMPA4 (alpha 4)	NM_001113180	1.09E-06	2.53

S3 Table: Differential changes in adrenal gene expression in ASP+CGP treated mice relative to ASP diet mice (≥±1.8 fold change, p <0.05).

Gene symbol	Gene name	RefSeq	P-value	Fold Change
Grid1	glutamate receptor, ionotropic, delta 1	NM_008166	2.10E-08	4.99
Grid2	glutamate receptor, ionotropic, delta 2	NM_008167	5.23E-07	3.10
Grik2	glutamate receptor, ionotropic, kainate 2 (beta 2)	NM_001111268	1.55E-07	3.16
Grik3	glutamate receptor, ionotropic, kainate 3	NM_001081097	4.22E-08	3.46
Grik5	glutamate receptor, ionotropic, kainate 5 (gamma 2)	NM_008168	6.89E-08	1.93
Grin1	glutamate receptor, ionotropic, NMDA1 (zeta 1)	NM_001177656	5.97E-11	3.28
Grin2a	glutamate receptor, ionotropic, NMDA2A (epsilon 1)	NM_008170	4.30E-06	2.96
Grin2b	glutamate receptor, ionotropic, NMDA2B (epsilon 2)	NM_008171	3.46E-09	4.79
Grin2d	glutamate receptor, ionotropic, NMDA2D (epsilon 4)	NM_008172	7.65E-06	1.99
Grin3a	glutamate receptor ionotropic, NMDA3A	NM_001033351	1.12E-10	3.23
Grm1	glutamate receptor, metabotropic 1	NM_001114333	8.93E-08	10.30
Grm2	glutamate receptor, metabotropic 2	NM_001160353	0.0002441	1.88
Grm3	glutamate receptor, metabotropic 3	NM_181850	7.70E-09	4.28
Grm4	glutamate receptor, metabotropic 4	NM_001013385	2.06E-06	1.84
Grm5	glutamate receptor, metabotropic 5	NM_001081414	1.56E-09	4.42
Grm7	glutamate receptor, metabotropic 7	NM_177328	9.30E-07	2.21
Gstm6	glutathione S-transferase, mu 6	NM_008184	5.17E-07	1.84
Gucy1b3	guanylate cyclase 1, soluble, beta 3	NM_001161796	5.14E-06	2.11
Habp4	hyaluronic acid binding protein 4	NM_019986	4.60E-06	2.12
Hamp	hepcidin antimicrobial peptide	NM_032541	1.37E-07	2.24
Hap1	huntingtin-associated protein 1	NM_010404	2.72E-08	2.34
Hapln4	hyaluronan and proteoglycan link protein 4	NM_177900	3.36E-06	2.52
Hcn1	hyperpolarization-activated, cyclic nucleotide-gated K+ 1	NM_010408	8.38E-09	6.17
Hcn2	hyperpolarization-activated, cyclic nucleotide-gated K+ 2	NM_008226	3.71E-08	3.83
Hcrt	hypocretin	NM_010410	6.61E-09	9.85
Hcst	Hcst hematopoietic cell signal transducer	NM_011827	7.56E-05	-1.89
Hdac11	histone deacetylase 11	NM_144919	3.13E-08	3.38
Hdac9	histone deacetylase 9	NM_024124	1.81E-06	2.06
Hecw1	HECT, C2 and WW domain containing E3 ubiquitin protein ligase	NM_001081348	3.74E-07	2.51
Hepacam	hepatocyte cell adhesion molecule	NM_175189	1.37E-09	7.66
Herc3	hect domain and RLD 3	NM_028705	1.53E-07	2.72
Hey1	hairy enhancer-of-split related with YRPW motif 1	NM_010423	3.17E-07	2.28
Hivep2	human immunodeficiency virus type I enhancer binding protein 2	NM_010437	2.17E-05	1.85
Hk1	hexokinase 1	NM_001146100	4.50E-08	2.02
Homer2	homer homolog 2 (Drosophila)	NM_001164086	1.13E-06	2.39
Hpca	hippocalcin	NM_001130419	2.28E-10	6.19
Hpcal4	hippocalcin-like 4	NM_174998	4.16E-10	5.07
Hrh3	histamine receptor H3	NM_133849	4.06E-09	3.60
Hs6st2	heparan sulfate 6-O-sulfotransferase 2	NM_001077202	1.43E-09	3.80
Htr1b	5-hydroxytryptamine (serotonin) receptor 1B	NM_010482	7.08E-08	2.10
Htr2c	5-hydroxytryptamine (serotonin) receptor 2C	NM_008312	6.16E-09	5.96
Htra1	HtrA serine peptidase 1	NM_019564	5.19E-07	2.22
Ids	iduronate 2-sulfatase	NM_010498	4.50E-09	2.07
Igsf1	immunoglobulin superfamily, member 1	NM_177591	4.98E-07	2.13
Igsf21	immunoglobulin superfamily, member 21	NM_198610	1.02E-07	2.58
Ildr2	immunoglobulin-like domain containing receptor 2	NM_001164528	8.71E-06	2.18
Impact	impact, RWD domain protein	NM_008378	1.93E-09	2.56
Ina	internexin neuronal intermediate filament protein, alpha	NM_146100	8.84E-11	3.89
Inpp4a	inositol polyphosphate-4-phosphatase, type I	NM_001290797	5.89E-09	2.07
Inpp5j	inositol polyphosphate 5-phosphatase J	NM_172439	2.70E-07	2.75
Iqsec3	IQ motif and Sec7 domain 3	NM_001033354	1.26E-07	3.01

S3 Table: Differential changes in adrenal gene expression in ASP+CGP treated mice relative to ASP diet mice (≥±1.8 fold change, p <0.05).

Gene symbol	Gene name	RefSeq	P-value	Fold Change
Irs4	insulin receptor substrate 4	NM_010572	0.0001244	2.06
Itgb8	integrin beta 8	NM_177290	3.44E-05	1.93
Itih3	inter-alpha trypsin inhibitor, heavy chain 3	NM_008407	4.33E-09	4.83
Itm2a	integral membrane protein 2A	NM_008409	5.15E-08	2.76
Jakmip2	janus kinase and microtubule interacting protein 2	NM_001163637	0.0001077	2.04
Jph3	junctophilin 3	NM_020605	1.09E-06	2.63
Katnal1	katanin p60 subunit A-like 1	NM_153572	6.74E-06	1.87
Kcna1	potassium voltage-gated channel, shaker-related subfamily	NM_010595	7.27E-07	3.11
Kcna2	potassium voltage-gated channel, shaker-related subfamily	NM_008417	1.78E-07	3.48
Kcna4	potassium voltage-gated channel, shaker-related subfamily	NM_021275	4.21E-05	2.13
Kcna6	potassium voltage-gated channel, shaker-related, subfamily	NM_013568	5.22E-11	4.43
Kcnab1	potassium voltage-gated channel, shaker-related subfamily	NM_001289450	1.17E-07	2.65
Kcnab2	potassium voltage-gated channel, shaker-related subfamily	NM_001252654	1.36E-08	4.35
Kcnc1	potassium voltage gated channel, Shaw-related subfamily)	NM_008421	4.40E-10	4.62
Kcnc2	potassium voltage gated channel, Shaw-related subfamily	NM_001025581	6.89E-09	6.44
Kcnd2	potassium voltage-gated channel, Shal-related, member 2	NM_019697	7.16E-08	4.44
Kcnd3	potassium voltage-gated channel, Shal-related family 3	NM_001039347	5.36E-07	2.01
Kcnh1	potassium voltage-gated channel, subfamily H1	NM_001038607	8.33E-08	2.02
Kcnh5	potassium voltage-gated channel, subfamily H (eag-related)	NM_172805	1.93E-08	2.10
Kcnip1	Kv channel-interacting protein 1	NM_001190885	2.52E-06	1.95
Kcnip2	Kv channel-interacting protein 2	NM_001276358	6.99E-07	2.10
Kcnip3	Kv channel interacting protein 3, calsenilin	NM_001111331	1.15E-07	2.54
Kcnip4	Kv channel interacting protein 4	NM_001199242	2.31E-10	6.44
Kcnj10	potassium inwardly-rectifying channel, subfamily J, member 10	NM_001039484	3.18E-08	7.78
Kcnj16	potassium inwardly-rectifying channel, subfamily J, member 16	NM_001252207	1.30E-07	2.71
Kcnj3	potassium inwardly-rectifying channel, subfamily J, member 3	NM_008426	1.67E-08	2.88
Kcnq2	potassium voltage-gated channel, subfamily Q, member 2	NM_001003824	2.35E-08	2.51
Kcnq5	potassium voltage-gated channel, subfamily Q, member 5	NM_001160139	1.60E-06	2.39
Kcnt1	potassium channel, subfamily T, member 1	NM_001145403	2.48E-10	2.10
Kctd3	potassium channel tetramerisation domain containing 3	NM_172650	0.000201	1.81
Kif1a	kinesin family member 1A	NM_001110315	7.47E-10	3.21
Kif1b	kinesin family member 1B	NM_008441	7.54E-08	2.06
Kif21a	kinesin family member 21A	NM_001109040	6.63E-08	2.10
Kif3a	kinesin family member 3A	NM_001290805	0.0001168	1.96
Kif3c	kinesin family member 3C	NM_008445	8.95E-06	1.94
Kif5a	kinesin family member 5A	NM_001039000	1.36E-10	11.51
Kif5c	kinesin family member 5C	NM_008449	2.20E-08	4.26
Kifap3	kinesin-associated protein 3	NM_010629	1.36E-09	2.56
Kifc2	kinesin family member C2	NM_010630	1.92E-11	2.51
Kirrel3	kin of IRRE like 3 (Drosophila)	NM_001190911	3.02E-06	2.23
Klc2	kinesin light chain 2	NM_008451	9.76E-07	1.82
Kndc1	kinase non-catalytic C-lobe domain (KIND) containing 1	NM_177261	3.30E-09	5.17
Krtap10-10	keratin associated protein 10-10	NM_001024709	6.86E-05	-2.10
Krtap10-4	keratin associated protein 10-4	NM_001135991	0.0004312	-1.96
Krtap1-3	keratin associated protein 1-3	NM_001085526	0.0001978	-1.82
Krtap5-3	keratin associated protein 5-3	NM_023860	0.0006283	-2.26
L1cam	L1 cell adhesion molecule	NM_008478	3.77E-10	3.16
Lamp5	lysosomal-associated membrane protein family, member 5	NM_029530	2.22E-06	2.50
Lancl2	LanC (bacterial lantibiotic synthetase component C)-like 2	NM_133737	3.64E-07	1.97
Lcat	lecithin cholesterol acyltransferase	NM_008490	2.48E-05	1.86
Ldhb	lactate dehydrogenase B	NM_008492	1.33E-08	2.38

S3 Table: Differential changes in adrenal gene expression in ASP+CGP treated mice relative to ASP diet mice (≥±1.8 fold change, p <0.05).

Gene symbol	Gene name	RefSeq	P-value	Fold Change
Lgi1	leucine-rich repeat LGI family, member 1	NM_020278	1.35E-08	11.82
Lgi2	leucine-rich repeat LGI family, member 2	NM_144945	1.51E-07	3.78
Lgi3	leucine-rich repeat LGI family, member 3	NM_145219	3.45E-10	4.12
Lin7b	lin-7 homolog B (C. elegans)	NM_011698	7.99E-07	1.90
Lingo1	leucine rich repeat and Ig domain containing 1	NM_181074	1.65E-09	4.13
Lingo2	leucine rich repeat and Ig domain containing 2	NM_001166001	2.20E-06	2.03
Lmo3	LIM domain only 3	NM_207222	6.44E-09	3.92
Lonrf2	LON peptidase N-terminal domain and ring finger 2	NM_001029878	8.53E-10	4.56
Lor	loricrin	NM_008508	5.65E-06	1.90
Lpcat4	lysophosphatidylcholine acyltransferase 4	NM_207206	1.14E-06	2.27
Lpgat1	lysophosphatidylglycerol acyltransferase 1	NM_001134829	4.12E-07	1.87
Lphn2	latrophilin 2	NM_001081298	1.48E-08	7.69
Lphn3	latrophilin 3	NM_198702	3.22E-09	6.48
Lrfn5	leucine rich repeat and fibronectin type III domain containing 5	NM_178714	3.71E-07	2.73
Lrig1	leucine-rich repeats and immunoglobulin-like domains 1	NM_008377	9.01E-08	2.02
Lrrc49	leucine rich repeat containing 49	NM_001146046	8.60E-08	1.90
Lrrc4c	leucine rich repeat containing 4C	NM_001289742	1.66E-06	3.13
Lrrc7	leucine rich repeat containing 7	NM_001081358	3.33E-09	5.35
Lrrn1	leucine rich repeat protein 1, neuronal	NM_008516	1.78E-07	2.39
Lrrtm1	leucine rich repeat transmembrane neuronal 1	NM_028880	1.24E-08	4.31
Lrrtm2	leucine rich repeat transmembrane neuronal 2	NM_178005	1.70E-07	3.53
Lrrtm3	leucine rich repeat transmembrane neuronal 3	NM_178678	3.15E-09	4.51
Lrrtm4	leucine rich repeat transmembrane neuronal 4	NM_001134743	5.34E-07	2.65
Lsamp	limbic system-associated membrane protein	NM_175548	3.98E-09	2.39
Luzp2	leucine zipper protein 2	NM_178705	2.27E-07	3.94
Lypd1	Ly6 Plaur domain containing 1	NM_145100	3.00E-06	1.82
Mag	myelin-associated glycoprotein	NM_010758	5.65E-10	10.05
Magee1	melanoma antigen, family E, 1	NM_053201	6.59E-07	2.69
Magi2	membrane associated guanylate kinase, WW and PDZ	NM_001170745	3.79E-07	2.70
Mal	myelin and lymphocyte protein, T cell differentiation protein	NM_001171187	8.84E-08	2.88
Mal2	mal, T cell differentiation protein 2	NM_178920	4.02E-10	8.61
Maneal	mannosidase, endo-alpha-like	NM_001007573	2.04E-06	2.13
Map1a	microtubule-associated protein 1 A	NM_001173506	2.90E-09	8.32
Map1b	microtubule-associated protein 1B	NM_008634	2.47E-09	2.54
Map2	microtubule-associated protein 2	NM_001039934	1.35E-08	4.43
Map2k4	mitogen-activated protein kinase kinase 4	NM_009157	2.33E-06	2.05
Map3k12	mitogen-activated protein kinase kinase kinase 12	NM_001163643	1.28E-08	2.16
Map6	microtubule-associated protein 6	NM_001048167	5.46E-09	2.42
Map6d1	MAP6 domain containing 1	NM_198599	6.60E-07	1.85
Map7d2	MAP7 domain containing 2	NM_001081124	9.47E-10	5.63
Mapk10	mitogen-activated protein kinase 10	NM_001081567	2.31E-11	6.45
Mapk4	mitogen-activated protein kinase 4	NM_172632	1.29E-08	2.99
Mapk8ip1	mitogen-activated protein kinase 8 interacting protein 1	NM_001202445	5.17E-08	2.27
Mapk8ip2	mitogen-activated protein kinase 8 interacting protein 2	NM_021921	3.03E-09	3.37
Mapk9	mitogen-activated protein kinase 9	NM_001163671	1.12E-05	1.89
Mapt	microtubule-associated protein tau	NM_001038609	7.02E-08	2.31
Mast1	microtubule associated serine threonine kinase 1	NM_019945	3.00E-11	4.48
Mbd5	methyl-CpG binding domain protein 5	NM_001290656	7.84E-07	1.87
Mboat2	membrane bound O-acyltransferase domain containing 2	NM_001083341	1.53E-06	2.17
Mbp	myelin basic protein	NM_001025245	9.81E-12	10.34
Mdga2	MAM domain containing glycosylphosphatidylinositol anchor 2	NM_001193266	2.48E-07	2.92

S3 Table: Differential changes in adrenal gene expression in ASP+CGP treated mice relative to ASP diet mice (≥±1.8 fold change, p <0.05).

Gene symbol	Gene name	RefSeq	P-value	Fold Change
Megf9	multiple EGF-like-domains 9	NM_172694	4.15E-06	1.86
Mfsd2a	major facilitator superfamily domain containing 2A	NM_029662	8.05E-11	3.64
Mgat4a	mannoside acetylglucosaminyltransferase 4, isoenzyme A	NM_001290801	1.25E-05	1.98
Mical2	microtubule associated monooxygenase, calponin and LIM domain	NM_001193305	1.13E-05	1.81
Micu3	mitochondrial calcium uptake family, member 3	NM_030110	5.42E-06	2.29
Mkl2	MKL myocardin-like 2	NM_001122667	2.17E-07	1.95
Mlc1	megalencephalic leukoencephalopathy with subcortical cysts 1	NM_133241	2.76E-08	7.11
Mlip	muscular LMNA-interacting protein	NM_027150	1.36E-06	2.28
Mllt11	myeloid lymphoid or mixed-lineage leukemia	NM_019914	1.31E-07	3.59
Mobp	myelin-associated oligodendrocytic basic protein	NM_001039364	2.06E-10	12.82
Mog	myelin oligodendrocyte glycoprotein	NM_010814	3.02E-08	5.27
Mt3	metallothionein 3	NM_013603	3.94E-08	2.61
Mtmr7	myotubularin related protein 7	NM_001040699	8.02E-08	2.46
Mtss1l	metastasis suppressor 1-like	NM_198625	4.09E-10	3.50
Myo5a	myosin VA	NM_010864	7.45E-07	1.96
Myrf	myelin regulatory factor	NM_001033481	8.23E-06	2.22
Myt1l	myelin transcription factor 1-like	NM_001093775	1.65E-05	2.17
Naa20	N(alpha)-acetyltransferase 20, NatB catalytic subunit	NM_001141965	2.76E-06	1.95
Nalcn	sodium leak channel, non-selective	NM_177393	1.45E-06	1.96
Nap1l2	nucleosome assembly protein 1-like 2	NM_008671	1.54E-07	4.59
Nap1l3	nucleosome assembly protein 1-like 3	NM_138742	6.29E-06	2.08
Nap1l5	nucleosome assembly protein 1-like 5	NM_021432	9.95E-11	4.06
Napb	N-ethylmaleimide sensitive fusion proteinβ	NM_019632	1.28E-11	9.07
Nat8l	N-acetyltransferase 8-like	NM_001001985	5.92E-09	3.39
Ncam2	neural cell adhesion molecule 2	NM_010954	6.84E-08	3.04
Ncan	neurocan	NM_007789	4.94E-07	2.70
Ncdn	neurochondrin	NM_011986	1.12E-09	4.37
Ncs1	neuronal calcium sensor 1	NM_019681	2.49E-09	6.78
Ndrg3	N-myc downstream regulated gene 3	NM_013865	2.93E-07	1.96
Ndrg4	N-myc downstream regulated gene 4	NM_001195006	2.09E-11	5.78
Ndst3	N-deacetylase N-sulfotransferase (heparan glucosaminyl) 3	NM_001293682	0.0001826	1.92
Ndst4	N-deacetylase N-sulfotransferase (heparin glucosaminyl) 4	NM_022565	2.25E-07	3.33
Necab1	N-terminal EF-hand calcium binding protein 1	NM_178617	8.88E-07	2.62
Necap1	NECAP endocytosis associated 1	NM_026267	1.13E-07	1.98
Nedd4l	neural precursor cell expressed, developmentally down-regulated 4l	NM_001114386	5.13E-08	3.46
Nefh	neurofilament, heavy polypeptide	NM_010904	2.64E-06	1.86
Nefl	neurofilament, light polypeptide	NM_010910	2.04E-10	11.04
Nefm	neurofilament, medium polypeptide	NM_008691	1.87E-08	3.49
Negr1	neuronal growth regulator 1	NM_001039094	5.92E-10	2.32
Nell1	NEL-like 1	NM_001037906	2.56E-08	3.82
Nell2	NEL-like 2	NM_001289653	2.25E-11	6.52
Neto1	neuropilin (NRP) and tolloid (TLL)-like 1	NM_144946	1.19E-10	6.80
Neto2	neuropilin (NRP) and tolloid (TLL)-like 2	NM_001081324	5.68E-06	2.42
Nfasc	neurofascin	NM_001160316	7.38E-11	3.53
Ngb	neuroglobin	NM_001294308	0.0002786	1.82
Nipa1	non imprinted in Prader-Willi Angelman syndrome 1 homolog	NM_153578	2.21E-06	2.15
Nkain2	Na+ K+ transporting ATPase interacting 2	NM_001013411	5.44E-09	5.43
Nkain4	Na+ K+ transporting ATPase interacting 4	NM_001141933	1.04E-07	2.54
Nkiras1	NFKB inhibitor interacting Ras-like protein 1	NM_023526	1.64E-05	2.19
Nlgn1	neuroligin 1	NM_001163387	1.46E-10	4.04
Nlgn2	neuroligin 2	NM_198862	3.02E-07	2.10

S3 Table: Differential changes in adrenal gene expression in ASP+CGP treated mice relative to ASP diet mice (≥±1.8 fold change, p <0.05).

Gene symbol	Gene name	RefSeq	P-value	Fold Change
Nlgn3	neuroligin 3	NM_172932	1.18E-07	1.96
Nlrc5	NLR family, CARD domain containing 5	NM_001033207	4.44E-05	-2.07
Nmnat2	nicotinamide nucleotide adenylyltransferase 2	NM_175460	8.42E-09	5.17
Nnat	neuronatin	NM_001291128	4.66E-08	2.10
Nos1	nitric oxide synthase 1, neuronal	NM_008712	2.72E-05	1.83
Nova1	neuro-oncological ventral antigen 1	NM_021361	2.13E-11	10.04
Nptn	neuroplastin	NM_001293673	2.36E-09	1.88
Nptx1	neuronal pentraxin 1	NM_008730	1.30E-06	2.23
Nrcam	neuron-glia-CAM-related cell adhesion molecule	NM_001146031	5.81E-07	3.40
Nrg3	neuregulin 3	NM_001190187	6.77E-06	2.00
Nrgn	neurogranin	NM_022029	5.06E-07	3.50
Nrip2	nuclear receptor interacting protein 2	NM_001162858	1.24E-06	2.51
Nrip3	nuclear receptor interacting protein 3	NM_020610	2.31E-08	4.17
Nrn1	neuritin 1	NM_153529	4.49E-08	3.39
Nrsn1	neurensin 1	NM_009513	3.25E-09	6.59
Nrsn2	neurensin 2	NM_001009948	2.27E-09	4.00
Nrxn1	neurexin I	NM_020252	1.61E-08	3.48
Nrxn2	neurexin II	NM_001205234	7.75E-10	4.13
Nrxn3	neurexin III	ENSMUST00000057634	3.00E-09	10.10
Nsf	N-ethylmaleimide sensitive fusion protein	NM_008740	1.08E-08	2.67
Nsg1	neuron specific gene family member 1	NM_010942	1.07E-08	2.74
Nsg2	neuron specific gene family member 2	XM_006514581	8.41E-10	2.69
Nsmf	NMDA receptor synaptonuclear signaling and neuronal migration	NM_001039386	2.29E-07	1.98
Ntm	neurotrimin	NM_172290	4.84E-11	10.59
Ntng1	netrin G1	NM_001163348	2.58E-09	5.13
Ntrk2	neurotrophic tyrosine kinase, receptor, type 2	NM_008745	8.08E-10	4.80
Ntrk3	neurotrophic tyrosine kinase, receptor, type 3	NM_008746	1.01E-09	2.45
Ntsr1	neurotensin receptor 1	NM_018766	1.31E-06	2.15
Ntsr2	neurotensin receptor 2	NM_008747	3.46E-09	6.60
Nudt10	nudix (nucleoside diphosphate linked moiety X)-type motif 10	NM_001031664	1.09E-05	3.12
Nudt11	nudix (nucleoside diphosphate linked moiety X)-type motif 11	NM_021431	0.0011802	1.89
Nup210l	nucleoporin 210-like	NM_029937	0.0001202	-1.93
Nxph1	neurexophilin 1	NM_008751	0.0006201	2.14
Ociad2	OCIA domain containing 2	NM_026950	5.51E-07	1.96
Ogdhl	oxoglutarate dehydrogenase-like	NM_001081130	2.23E-09	2.85
Ogfod1	2-oxoglutarate and iron-dependent oxygenase domain	NM_001093757	8.09E-08	2.66
Ogfrl1	opioid growth factor receptor-like 1	NM_001081079	2.17E-07	2.22
Olfr314	olfactory receptor 314	NM_001011760	0.0001693	-1.99
Olfr401	olfactory receptor 401	NM_146706	3.84E-05	-1.89
Olfr91	olfactory receptor 91	NM_182714	0.0004257	-1.85
Olig1	oligodendrocyte transcription factor 1	NM_016968	1.24E-09	6.53
Olig2	oligodendrocyte transcription factor 2	NM_016967	6.36E-06	2.72
Omg	oligodendrocyte myelin glycoprotein	NM_019409	9.12E-08	3.07
Opalin	oligodendrocytic myelin paranodal and inner loop protein	NM_153520	1.86E-07	3.46
Opcml	opioid binding protein cell adhesion molecule-like	NM_177906	2.44E-10	9.55
Oprl1	opioid receptor-like 1	NM_001252565	1.72E-08	2.94
Oscp1	organic solute carrier partner 1	NM_172701	2.76E-06	2.03
Otud7a	OTU domain containing 7A	NM_130880	5.29E-05	2.00
Oxt	oxytocin	NM_011025	1.05E-06	3.35
Pacsin1	protein kinase C and casein kinase substrate in neurons 1	NM_001286743	5.78E-09	3.71
Pak1	p21 protein (Cdc42 Rac)-activated kinase 1	NM_011035	2.46E-08	2.93

S3 Table: Differential changes in adrenal gene expression in ASP+CGP treated mice relative to ASP diet mice (≥±1.8 fold change, p <0.05).

Gene symbol	Gene name	RefSeq	P-value	Fold Change
Pcdh10	protocadherin 10	NM_001098171	1.49E-07	3.23
Pcdh11x	protocadherin 11 X-linked	NM_001271809	5.29E-07	4.19
Pcdh17	protocadherin 17	NM_001013753	1.60E-06	2.50
Pcdh19	protocadherin 19	NM_001105245	3.56E-05	2.35
Pcdh7	protocadherin 7	NM_001122758	1.26E-07	2.54
Pcdh9	protocadherin 9	NM_001081377	3.52E-10	3.93
Pclo	piccolo (presynaptic cytomatrix protein)	NM_001110796	6.66E-05	1.80
Pcnxl2	pecanex-like 2 (Drosophila)	NM_175561	1.58E-06	2.42
Pcolce2	procollagen C-endopeptidase enhancer 2	NM_029620	1.62E-05	1.83
Pcp4	Purkinje cell protein 4	NM_008791	3.98E-10	4.88
Pcp4l1	Purkinje cell protein 4-like 1	NM_025557	1.18E-07	1.94
Pcsk2	proprotein convertase subtilisin kexin type 2	NM_008792	1.29E-11	6.49
Pde1b	phosphodiesterase 1B, Ca2+-calmodulin dependent	NM_001285890	5.19E-09	3.28
Pde4b	phosphodiesterase 4B, cAMP specific	NM_001177980	1.70E-10	4.04
Pde4d	phosphodiesterase 4D, cAMP specific	NM_011056	6.61E-05	1.89
Pde7b	phosphodiesterase 7B	NM_013875	6.33E-08	1.97
Pde9a	phosphodiesterase 9A	NM_001163748	7.99E-06	2.00
Pdxk	pyridoxal (pyridoxine, vitamin B6) kinase	NM_172134	3.72E-08	2.07
Pdxp	pyridoxal (pyridoxine, vitamin B6) phosphatase	NM_020271	4.14E-10	4.71
Pea15a	phosphoprotein enriched in astrocytes 15A	NM_011063	8.52E-09	2.42
Peg3	paternally expressed 3	NM_008817	2.14E-10	3.42
Penk	preproenkephalin	NM_001002927	1.17E-08	2.10
Pex5l	peroxisomal biogenesis factor 5-like	NM_001163516	3.49E-07	2.49
Pfkm	phosphofructokinase, muscle	NM_001163487	1.10E-09	2.25
Pfkp	phosphofructokinase, platelet	NM_001291071	2.87E-07	2.11
Pfn2	profilin 2	NM_019410	6.47E-10	2.86
Pgap1	post-GPI attachment to proteins 1	NM_001163314	4.65E-06	2.91
Pglyrp1	peptidoglycan recognition protein 1	NM_009402	0.0001404	1.89
Pgm2l1	phosphoglucomutase 2-like 1	NM_027629	3.46E-08	3.49
Phactr1	phosphatase and actin regulator 1	NM_001005740	7.64E-09	3.08
Phlpp1	PH domain and leucine rich repeat protein phosphatase 1	NM_133821	3.28E-08	2.80
Phyhip	phytanoyl-CoA hydroxylase interacting protein	NM_145981	5.48E-10	5.80
Phyhipl	phytanoyl-CoA hydroxylase interacting protein-like	NM_001162846	7.44E-11	9.15
Pi4ka	phosphatidylinositol 4-kinase, catalytic, alpha polypeptide	NM_001001983	2.55E-07	1.82
Pigz	phosphatidylinositol glycan anchor biosynthesis, class Z	NM_172822	2.96E-06	1.98
Pip4k2a	phosphatidylinositol-5-phosphate 4-kinase, type II, alpha	NM_008845	1.93E-07	1.89
Pip4k2b	phosphatidylinositol-5-phosphate 4-kinase, type II, beta	NM_054051	1.57E-06	1.84
Pitpnc1	phosphatidylinositol transfer protein, cytoplasmic 1	NM_145823	6.33E-06	2.19
Pja2	praja 2, RING-H2 motif containing	NM_001025309	1.04E-06	1.95
Pkia	protein kinase inhibitor, alpha	NM_008862	5.99E-09	4.45
Pkp4	plakophilin 4	NM_026361	4.01E-08	2.49
Pla2g2e	phospholipase A2, group IIE	NM_012044	2.13E-06	-1.85
Pla2g7	phospholipase A2, group VII (platelet-activating factor)	NM_013737	1.36E-08	1.92
Plagl1	pleiomorphic adenoma gene-like 1	NM_009538	5.81E-06	1.84
Plb1	phospholipase B1	NM_001081407	0.0006584	-1.83
Plcxd2	phosphatidylinositol-specific phospholipase C, X2	NM_001134480	6.43E-06	2.05
Plekhb1	pleckstrin homology domain containing, family B (evectins)	NM_001163182	5.54E-12	8.98
Plekhg1	pleckstrin homology domain containing, family G	NM_001033253	1.24E-06	2.13
Pllp	plasma membrane proteolipid	NM_026385	1.61E-05	2.67
Plp1	proteolipid protein (myelin) 1	NM_001290561	2.35E-11	19.58
Plxnc1	plexin C1	NM_018797	5.20E-06	2.50

S3 Table: Differential changes in adrenal gene expression in ASP+CGP treated mice relative to ASP diet mice (≥±1.8 fold change, p <0.05).

Gene symbol	Gene name	RefSeq	P-value	Fold Change
Pmch	pro-melanin-concentrating hormone	NM_029971	4.43E-10	17.52
Pnck	pregnancy upregulated non-ubiquitously expressed CaM kinase	NM_001199351	3.88E-08	3.00
Pnkd	paroxysmal nonkinesiogenic dyskinesia	NM_019999	2.10E-08	2.61
Pnma2	paraneoplastic antigen MA2	NM_175498	2.49E-06	1.89
Pnma3	paraneoplastic antigen MA3	NM_153169	1.38E-05	2.35
Pnmal1	PNMA-like 1	NM_001007569	7.45E-08	2.86
Pnmal2	PNMA-like 2	NM_001099636	9.01E-08	3.04
Pou3f4	POU domain, class 3, transcription factor 4	NM_008901	6.77E-07	1.89
Ppargc1a	peroxisome proliferative activated receptor, gamma	NM_008904	1.61E-09	3.95
Ppp1r12b	protein phosphatase 1, regulatory (inhibitor) subunit 12B	NM_001081307	1.10E-06	1.93
Ppp1r16b	protein phosphatase 1, regulatory (inhibitor) subunit 16B	NM_001159662	8.18E-09	2.21
Ppp1r1b	protein phosphatase 1, regulatory (inhibitor) subunit 1B	NM_144828	7.26E-07	2.66
Ppp1r3c	protein phosphatase 1, regulatory (inhibitor) subunit 3C	NM_016854	1.45E-05	2.20
Ppp1r9b	protein phosphatase 1, regulatory subunit 9B	NM_172261	1.81E-08	1.97
Ppp2r2b	protein phosphatase 2, regulatory subunit B, beta	NR_073583	1.88E-07	2.37
Ppp2r2c	protein phosphatase 2, regulatory subunit B, gamma	NM_172994	2.08E-09	6.04
Ppp2r5c	protein phosphatase 2, regulatory subunit B, gamma	NM_001135001	7.98E-06	1.97
Ppp3ca	protein phosphatase 3, catalytic subunit, alpha isoform	NM_001293622	2.23E-09	2.67
Ppy	pancreatic polypeptide	NM_008918	0.0007232	-1.87
Praf2	PRA1 domain family 2	NM_138602	4.00E-07	1.93
Prepl	prolyl endopeptidase-like	NM_001163622	4.97E-10	5.58
Prg4	proteoglycan 4	NM_001110146	1.20E-06	2.23
Prickle2	prickle homolog 2 (Drosophila)	NM_001081146	1.44E-06	2.65
Prkacb	protein kinase, cAMP dependent, catalytic, beta	NM_001164198	8.49E-10	1.89
Prkar1b	protein kinase, cAMP dependent regulatory, type I beta	NM_001253890	3.73E-11	3.09
Prkca	protein kinase C, alpha	NM_011101	2.08E-07	2.20
Prkcb	protein kinase C, beta	NM_008855	8.55E-09	3.56
Prkce	protein kinase C, epsilon	NM_011104	5.19E-07	2.38
Prkcz	protein kinase C, zeta	NM_001039079	1.65E-10	5.35
Prlh	prolactin releasing hormone	NM_001101647	0.0017631	-1.81
Prmt8	protein arginine N-methyltransferase 8	NM_201371	6.80E-08	2.32
Prnp	prion protein	NM_001278256	1.21E-10	2.17
Prp2	proline rich protein 2	NM_031499	0.0012366	-2.31
Prrt2	proline-rich transmembrane protein 2	NM_001102563	9.76E-09	2.51
Psd	pleckstrin and Sec7 domain containing	NM_028627	8.70E-09	2.41
Psd2	pleckstrin and Sec7 domain containing 2	NM_001289602	1.58E-08	3.57
Ptbp2	polypyrimidine tract binding protein 2	NM_019550	2.25E-08	1.90
Ptch1	patched homolog 1	NM_008957	7.59E-09	2.05
Ptchd1	patched domain containing 1	NM_001093750	1.24E-05	2.13
Ptgds	prostaglandin D2 synthase (brain)	NM_008963	2.65E-11	17.44
Ptk2b	PTK2 protein tyrosine kinase 2 beta	NM_001162365	4.22E-07	2.30
Ptpn5	protein tyrosine phosphatase, non-receptor type 5	NM_001163565	1.67E-05	2.65
Ptpro	protein tyrosine phosphatase, receptor type, O	NM_001164401	5.99E-09	2.98
Ptprt	protein tyrosine phosphatase, receptor type, T	NM_001291149	8.34E-06	2.05
Ptprz1	protein tyrosine phosphatase, receptor type Z, polypeptide 1	NM_001081306	2.63E-08	6.49
Purb	purine rich element binding protein B	NM_011221	3.78E-06	2.12
Pvalb	parvalbumin	NM_013645	2.44E-08	4.62
Pydc4	pyrin domain containing 4	NM_001177349	0.0006518	-2.00
Pygb	brain glycogen phosphorylase	NM_153781	1.36E-06	1.89
Rab11fip4	RAB11 family interacting protein 4 (class II)	NM_175543	2.93E-06	2.07
Rab15	RAB15, member RAS oncogene family	NM_134050	8.72E-11	3.57

S3 Table: Differential changes in adrenal gene expression in ASP+CGP treated mice relative to ASP diet mice (≥±1.8 fold change, p <0.05).

Gene symbol	Gene name	RefSeq	P-value	Fold Change
Rab39b	RAB39B, member RAS oncogene family	NM_175122	1.52E-05	2.11
Rab3a	RAB3A, member RAS oncogene family	NM_001166399	1.14E-09	2.30
Rab3c	RAB3C, member RAS oncogene family	NM_023852	1.22E-06	2.16
Rab6b	RAB6B, member RAS oncogene family	NM_173781	1.17E-11	3.68
Rab9b	RAB9B, member RAS oncogene family	NM_176971	3.25E-06	2.23
Rac3	RAS-related C3 botulinum substrate 3	NM_133223	9.13E-10	2.57
Ralgps1	Ral GEF with PH domain and SH3 binding motif 1	NM_001290570	5.40E-07	2.13
Ralyl	RALY RNA binding protein-like	NM_001163328	1.71E-07	3.81
Rangap1	RAN GTPase activating protein 1	NM_011241	9.27E-08	1.89
Rap1gds1	RAP1, GTP-GDP dissociation stimulator 1	NM_001040690	3.89E-08	2.03
Rapgef2	Rap guanine nucleotide exchange factor (GEF) 2	NM_001099624	7.18E-07	1.96
Rapgefl1	Rap guanine nucleotide exchange factor (GEF)-like 1	NM_001080925	3.83E-06	2.45
Rasd2	RASD family, member 2	NM_029182	3.39E-06	2.95
Rasgef1a	RasGEF domain family, member 1A	NM_027526	1.95E-09	10.98
Rasgrf1	RAS protein-specific guanine nucleotide-releasing factor 1	NM_011245	5.57E-11	2.42
Rasgrf2	RAS protein-specific guanine nucleotide-releasing factor 2	NM_009027	3.83E-10	4.91
Rasgrp1	RAS guanyl releasing protein 1	NM_011246	1.12E-10	11.98
Rbbp6	retinoblastoma binding protein 6	NM_175023	0.0001944	1.83
Rbfox1	RNA binding protein, fox-1 homolog (C. elegans) 1	NM_021477	1.33E-09	5.73
Rbfox3	RNA binding protein, fox-1 homolog (C. elegans) 3	NM_001039167	1.88E-09	3.85
Rcan2	regulator of calcineurin 2	NM_001286654	3.84E-12	7.18
Reep1	receptor accessory protein 1	NM_178608	3.99E-07	2.26
Rell2	RELT-like 2	NM_153793	1.93E-08	3.25
Reln	reelin	NM_011261	7.24E-07	1.98
Reps2	RALBP1 associated Eps domain containing protein 2	NM_001290633	6.57E-09	4.11
Ret	ret proto-oncogene	NM_001080780	2.94E-08	2.31
Rgs10	regulator of G-protein signalling 10	NM_026418	1.32E-05	2.01
Rgs7	regulator of G protein signaling 7	NM_001199003	1.90E-08	2.94
Rgs7bp	regulator of G-protein signalling 7 binding protein	NM_029879	3.07E-09	4.17
Rgs8	regulator of G-protein signaling 8	NM_026380	6.37E-09	3.34
Rhobtb2	Rho-related BTB domain containing 2	NM_153514	4.04E-09	2.32
Rhof	ras homolog gene family, member f	NM_175092	2.48E-05	2.07
Rimbp2	RIMS binding protein 2	NM_001081388	6.49E-06	1.91
Rimklb	ribosomal modification protein rimK-like family member B	NM_027664	1.82E-08	8.26
Rims1	regulating synaptic membrane exocytosis 1	NM_001012623	3.12E-06	2.73
Rit2	Ras-like without CAAX 2	NM_009065	6.92E-09	3.88
Rnd2	Rho family GTPase 2	NM_009708	3.24E-08	3.51
Rnf14	ring finger protein 14	NM_001164621	4.96E-08	2.18
Rnf157	ring finger protein 157	NM_027258	1.47E-10	4.25
Rnf208	ring finger protein 208	NM_176834	7.57E-09	2.50
Rny1	RNA, Y1 small cytoplasmic, Ro-associated	NR_004419	0.0001502	1.97
Rph3a	rabphilin 3A	NM_011286	1.04E-07	2.54
Rpp25	ribonuclease P MRP 25 subunit	NM_133982	2.98E-07	2.62
Rragb	Ras-related GTP binding B	NM_001004154	3.42E-06	2.52
Rtn1	reticulon 1	NM_001007596	1.53E-10	5.97
Rtn2	reticulon 2 (Z-band associated protein)	NM_001025364	1.48E-08	2.10
Rufy3	RUN and FYVE domain containing 3	NM_001289775	2.78E-08	2.48
Runx1t1	runt-related transcription factor 1; translocated to, 1	NM_001111026	1.37E-07	2.49
Rwdd2a	RWD domain containing 2A	NM_001145968	3.86E-08	2.84
Rxrg	retinoid X receptor gamma	NM_001159731	1.36E-07	2.32
S100b	S100 protein, beta polypeptide, neural	NM_009115	8.61E-08	4.29

S3 Table: Differential changes in adrenal gene expression in ASP+CGP treated mice relative to ASP diet mice (≥±1.8 fold change, p <0.05).

Gene symbol	Gene name	RefSeq	P-value	Fold Change
Sbpl	spermine binding protein-like	NM_001077421	3.05E-05	-1.85
Scamp1	secretory carrier membrane protein 1	NM_029153	1.40E-08	1.80
Scamp5	secretory carrier membrane protein 5	NM_020270	1.35E-09	3.38
Sccpdh	saccharopine dehydrogenase (putative)	NM_178653	1.15E-06	2.11
Scg3	secretogranin III	NM_001164790	8.43E-07	2.67
Scn1a	sodium channel, voltage-gated, type I, alpha	NM_018733	2.97E-05	3.07
Scn1b	sodium channel, voltage-gated, type I, beta	NM_011322	6.10E-08	2.89
Scn2a1	sodium channel, voltage-gated, type II, alpha 1	NM_001099298	2.43E-11	15.60
Scn2b	sodium channel, voltage-gated, type II, beta	NM_001014761	2.48E-10	4.70
Scn3a	sodium channel, voltage-gated, type III, alpha	NM_018732	2.73E-06	2.27
Scn3b	sodium channel, voltage-gated, type III, beta	NM_001083917	6.27E-09	2.88
Scn4b	sodium channel, type IV, beta	NM_001013390	7.88E-06	2.37
Scn8a	sodium channel, voltage-gated, type VIII, alpha	NM_001077499	1.67E-08	3.33
Scoc	short coiled-coil protein	NM_001039137	5.48E-05	2.26
Scrg1	scrapie responsive gene 1	NM_009136	1.34E-09	7.46
Scrn1	secernin 1	NM_027268	5.29E-09	2.09
Scrt1	scratch homolog 1, zinc finger protein (Drosophila)	NM_130893	1.36E-07	2.26
Sema6a	sema domain, transmembrane domain (TM), and cytoplasmic domain,	NM_018744	1.07E-06	1.89
Sema6d	sema domain, transmembrane domain (TM), and cytoplasmic 6d	NM_001290997	2.74E-08	2.34
Sept3	septin 3	NM_011889	9.88E-10	7.28
Sept4	septin 4	NM_001284392	7.59E-08	2.28
Serpine2	serine (or cysteine) peptidase inhibitor, clade E, member 2	NM_009255	2.70E-09	4.15
Sez6	seizure related gene 6	NM_001291225	1.30E-06	2.53
Sez6l	seizure related 6 homolog like	NM_001253916	1.43E-08	2.39
Sez6l2	seizure related 6 homolog like 2	NM_001252566	8.20E-09	2.33
Sgip1	SH3-domain GRB2-like (endophilin) interacting protein 1	NM_001285852	3.50E-12	3.75
Sgsm1	small G protein signaling modulator 1	NM_001162965	3.76E-08	2.10
Sgtb	small glutamine-rich tetratricopeptide repeat (TPR)-containing	NM_144838	6.25E-08	2.32
Sh3gl2	SH3-domain GRB2-like 2	NM_019535	1.49E-07	2.14
Shank1	SH3 ankyrin domain gene 1	NM_001034115	6.77E-10	4.81
Shc3	src homology 2 domain-containing transforming protein C3	NM_009167	3.13E-10	6.55
Shd	src homology 2 domain-containing transforming protein D	NM_001159523	2.45E-06	1.99
Shisa6	shisa homolog 6 (Xenopus laevis)	NM_001034874	7.48E-06	3.02
Shisa7	shisa homolog 7 (Xenopus laevis)	NM_001290291	4.92E-06	1.96
Sipa1l1	signal-induced proliferation-associated 1 like 1	NM_001167983	3.44E-07	2.05
Slain1	SLAIN motif family, member 1	NM_198014	2.02E-06	3.73
Slc12a5	solute carrier family 12, member 5	NM_020333	1.77E-11	6.22
Slc14a1	solute carrier family 14 (urea transporter), member 1	NM_001171010	1.01E-06	2.59
Slc17a6	solute carrier family 17	NM_080853	6.41E-08	8.21
Slc1a1	solute carrier family 1 (neuronal epithelial high affinity glu)	NM_009199	5.91E-09	4.43
Slc1a2	solute carrier family 1 (glial high affinity glutamate transport 2)	NM_011393	6.92E-11	13.09
Slc1a3	solute carrier family 1 (glial high affinity glutamate transport 3)	NM_148938	1.99E-09	5.25
Slc20a1	solute carrier family 20, member 1	NM_001159593	2.15E-07	2.38
Slc22a17	solute carrier family 22 (organic cation transporter), member	NM_021551	2.12E-08	2.28
Slc22a23	solute carrier family 22, member 23	NM_001033167	2.06E-07	2.55
Slc22a8	solute carrier family 22 (organic anion transporter), member	NM_001164634	1.14E-06	2.25
Slc24a2	solute carrier family 24 (sodium potassium	NM_001110240	3.57E-09	2.64
Slc24a3	solute carrier family 24 (sodium potassium	NM_053195	1.45E-08	4.20
Slc25a12	solute carrier family 25 (mitochondrial carrier, Aralar)	NM_172436	2.32E-06	1.84
Slc25a18	solute carrier family 25 (mitochondrial carrier), member 18	NM_001081048	8.89E-09	5.92
Slc25a4	solute carrier family 25 (mitochondrial carrier), member 18	NM_007450	1.13E-07	1.93

S3 Table: Differential changes in adrenal gene expression in ASP+CGP treated mice relative to ASP diet mice (≥±1.8 fold change, p <0.05).

Gene symbol	Gene name	RefSeq	P-value	Fold Change
Slc2a1	solute carrier family 2 (facilitated glucose transporter)	NM_011400	2.27E-08	1.97
Slc2a13	solute carrier family 2 (facilitated glucose transporter)	NM_001033633	5.97E-08	3.69
Slc2a3	solute carrier family 2 (facilitated glucose transporter)	NM_011401	5.15E-09	2.12
Slc32a1	solute carrier family 32 (GABA vesicular transporter), member 1	NM_009508	6.06E-08	3.11
Slc34a1	solute carrier family 34 (sodium phosphate), member 1	NM_011392	3.08E-05	-1.90
Slc35e3	solute carrier family 35, member E3	NM_029875	9.20E-06	2.14
Slc35f1	solute carrier family 35, member F1	NM_178675	6.30E-09	4.13
Slc35f3	solute carrier family 35, member F3	NM_175434	3.08E-06	1.96
Slc35g2	solute carrier family 35, member G2	NM_001101483	1.43E-05	1.95
Slc38a1	solute carrier family 38, member 1	AF184240	1.47E-10	3.75
Slc38a3	solute carrier family 38, member 3	NM_001199217	2.32E-09	2.81
Slc39a12	solute carrier family 39 (zinc transporter), member 12	NM_001012305	7.78E-08	2.69
Slc39a5	solute carrier family 39 (metal ion transporter), member 5	NM_028051	1.95E-05	-1.89
Slc4a10	solute carrier family 4, sodium bicarbonate cotransporter	NM_001242378	2.63E-08	3.70
Slc4a4	solute carrier family 4 (anion exchanger), member 4	NM_001136260	3.00E-09	2.97
Slc4a8	solute carrier family 4 (anion exchanger), member 8	NM_021530	2.21E-06	1.90
Slc6a1	solute carrier family 6 (neurotransmitter trans, GABA), m1	NM_178703	4.61E-11	23.11
Slc6a11	solute carrier family 6 (neurotransmitter transporter, GABA)	NM_172890	2.75E-13	38.37
Slc6a13	solute carrier family 6 (neurotransmitter transporter, GABA)	NM_144512	6.20E-07	2.48
Slc6a15	solute carrier family 6 (neurotransmitter transporter), memb	NM_001252330	3.00E-06	2.39
Slc6a17	solute carrier family 6 (neurotransmitter transporter)	NM_001293689	5.18E-10	4.07
Slc6a3	solute carrier family 6 (neurotransmitter, dopamine)	NM_010020	3.46E-08	6.15
Slc6a7	solute carrier family 6 (neurotransmitter transporter, L-proline)	NM_201353	5.04E-07	2.14
Slc6a9	solute carrier family 6 (neurotransmitter transporter, glycine),	NM_008135	2.15E-07	1.99
Slc7a11	solute carrier family 7 (cationic amino acid transporter	NM_011990	1.18E-07	3.08
Slc7a14	solute carrier family 7 (cationic amino acid transporter, y+ sy	NM_172861	3.33E-08	2.72
Slc7a3	solute carrier family 7 (cationic amino acid transporter, y+ sys	NM_007515	2.62E-05	2.55
Slc8a1	solute carrier family 8 (sodium calcium exchanger), member 1	NM_001112798	4.54E-06	1.82
Slc8a3	solute carrier family 8 (sodium calcium exchanger), member 3	NM_001167920	7.95E-09	2.10
Slc9a6	solute carrier family 9 (sodium hydrogen exchanger), member 6	NM_172780	9.36E-07	1.82
Slco1a4	solute carrier organic anion transporter family, member 1a4	NM_030687	5.47E-06	3.34
Slco1c1	solute carrier organic anion transporter family, member 1c1	NM_001177772	2.21E-06	2.36
Slitrk1	SLIT and NTRK-like family, member 1	NM_199065	7.65E-05	2.82
Slitrk3	SLIT and NTRK-like family, member 3	NM_198864	6.62E-08	5.32
Slmo1	slowmo homolog 1 (Drosophila)	NM_144867	1.12E-05	1.85
Sms	spermine synthase	NM_009214	4.58E-06	2.57
Snap25	synaptosomal-associated protein 25	NM_001291056	3.73E-11	3.74
Snap47	synaptosomal-associated protein, 47	NM_144521	4.65E-08	1.90
Snap91	synaptosomal-associated protein 91	NM_001277982	2.00E-08	2.02
Snca	synuclein, alpha	NM_001042451	1.63E-08	2.55
Sncb	synuclein, beta	NM_033610	3.87E-10	5.01
Snhg11	small nucleolar RNA host gene 11	NM_175692	1.19E-08	3.89
Snord116	small nucleolar RNA, C D box 116	NR_002895	5.11E-09	4.34
Snrpn	small nuclear ribonucleoprotein N	NM_001082961	2.88E-10	5.11
Sntg1	syntrophin, gamma 1	NM_001290390	2.33E-05	1.81
Snx32	sorting nexin 32	NM_001024560	1.97E-07	2.51
Sobp	sine oculis-binding protein homolog (Drosophila)	NM_175407	2.52E-09	2.31
Sorcs3	sortilin-related VPS10 domain containing receptor 3	NM_025696	1.08E-05	2.46
Sorl1	sortilin-related receptor, LDLR class A repeats-containing	NM_011436	1.72E-07	1.99
Sort1	sortilin 1	NM_001271599	2.13E-07	2.01
Sowaha	sosondowah ankyrin repeat domain family member A	NM_183173	4.95E-07	2.50

S3 Table: Differential changes in adrenal gene expression in ASP+CGP treated mice relative to ASP diet mice (≥±1.8 fold change, p <0.05).

Gene symbol	Gene name	RefSeq	P-value	Fold Change
Sox2	SRY (sex determining region Y)-box 2	NM_011443	4.02E-06	3.99
Sox9	SRY (sex determining region Y)-box 9	NM_011448	3.41E-07	2.01
Sparcl1	SPARC-like 1	NM_010097	2.10E-10	6.83
Sphkap	SPHK1 interactor, AKAP domain containing	NM_172430	3.83E-07	2.27
Spire1	spire homolog 1 (Drosophila)	NM_176832	1.45E-07	2.47
Spock1	sparc osteonectin, cwcv and kazal-like domains proteoglycan 1	NM_001166463	6.31E-09	2.70
Spock2	sparc osteonectin, cwcv and kazal-like domains proteoglycan 2	NM_052994	7.63E-08	2.35
Spock3	sparc osteonectin, cwcv and kazal-like domains proteoglycan 3	NM_001252620	2.99E-08	2.40
Spon1	spondin 1, (f-spondin) extracellular matrix protein	NM_145584	1.15E-07	2.01
Sprn	shadow of prion protein	NM_183147	3.60E-07	2.75
Sprr1a	small proline-rich protein 1A	NM_009264	8.61E-06	-1.95
Spry3	sprouty homolog 3 (Drosophila)	NM_001030293	5.90E-05	1.87
Sptbn2	spectrin beta, non-erythrocytic 2	NM_021287	1.35E-08	1.84
Srgap1	SLIT-ROBO Rho GTPase activating protein 1	NM_001081037	1.45E-07	2.34
Srgap3	SLIT-ROBO Rho GTPase activating protein 3	NM_080448	1.92E-08	2.74
Srpk2	serine arginine-rich protein specific kinase 2	NM_009274	1.21E-09	1.97
Sry	sex determining region of Chr Y	NM_011564	0.0002709	-2.01
Ssbp2	single-stranded DNA binding protein 2	NM_024186	4.22E-09	2.02
Sst	somatostatin	NM_009215	8.03E-11	19.55
St6gal2	beta galactoside alpha 2,6 sialyltransferase 2	NM_172829	1.23E-08	4.38
St6galnac5	ST6 (alpha-N-acetyl-neuraminyl-2,3-beta-galactosyl-1,3)-N-ac-5	NM_012028	4.11E-05	2.06
St8sia1	ST8 alpha-N-acetyl-neuraminide alpha-2,8-sialyltransferase 1	NM_011374	1.57E-08	3.01
St8sia3	ST8 alpha-N-acetyl-neuraminide alpha-2,8-sialyltransferase 3	NM_009182	2.24E-09	3.22
Stk32c	serine threonine kinase 32C	NM_001162540	3.58E-06	1.99
Stmn1	stathmin 1	NM_019641	5.31E-10	6.72
Stmn2	stathmin-like 2	NM_025285	1.81E-09	3.96
Stmn3	stathmin-like 3	NM_009133	1.47E-09	4.49
Stmn4	stathmin-like 4	NM_019675	1.86E-07	2.31
Stox2	storkhead box 2	NM_001114311	1.29E-07	4.88
Stox2	storkhead box 2	NM_001114311	1.03E-05	1.84
Strbp	spermatid perinuclear RNA binding protein	NM_009261	2.28E-07	2.81
Strip2	striatin interacting protein 2	NM_001037740	3.36E-06	2.40
Stx1b	syntaxin 1B	NM_024414	2.61E-11	5.48
Stxbp1	syntaxin binding protein 1	NM_001113569	9.90E-12	4.71
Stxbp5l	syntaxin binding protein 5-like	NM_001114611	2.20E-06	2.18
Sult4a1	sulfotransferase family 4A, member 1	NM_013873	6.39E-10	4.78
Susd4	sushi domain containing 4	NM_144796	7.45E-09	5.82
Sv2a	synaptic vesicle glycoprotein 2 a	NM_022030	2.00E-10	8.24
Sv2b	synaptic vesicle glycoprotein 2 b	NM_001109753	1.35E-08	6.00
Sv2c	synaptic vesicle glycoprotein 2c	NM_029210	6.07E-08	3.64
Svop	SV2 related protein	NM_026805	4.22E-07	2.87
Syn1	synapsin I	NM_001110780	1.17E-09	4.43
Syndig1	synapse differentiation inducing 1	NM_001085521	1.78E-05	2.18
Syngap1	synaptic Ras GTPase activating protein 1 homolog (rat)	NM_001281491	1.19E-07	7.17
Syngr1	synaptogyrin 1	NM_009303	1.17E-08	1.97
Syngr3	synaptogyrin 3	NM_011522	3.67E-09	4.07
Synj1	synaptojanin 1	NM_001045515	1.42E-09	2.49
Syp	synaptophysin	NM_009305	4.80E-11	3.58
Syt1	synaptotagmin I	NM_001252341	2.90E-10	4.58
Syt10	synaptotagmin X	NM_018803	0.0001897	2.08
Syt11	synaptotagmin XI	NM_018804	1.42E-11	6.24

S3 Table: Differential changes in adrenal gene expression in ASP+CGP treated mice relative to ASP diet mice (≥±1.8 fold change, p <0.05).

Gene symbol	Gene name	RefSeq	P-value	Fold Change
Syt13	synaptotagmin XIII	NM_030725	3.11E-05	2.16
Syt3	synaptotagmin III	NM_001114116	7.65E-08	1.84
Syt4	synaptotagmin IV	NM_009308	1.56E-06	2.03
Syt5	synaptotagmin V	NM_016908	4.46E-10	3.94
Syt9	synaptotagmin IX	NM_021889	1.48E-06	1.88
Szt2	seizure threshold 2	NM_198170	7.03E-08	-1.88
Tac1	tachykinin 1	NM_009311	5.99E-10	6.32
Tac2	tachykinin 2	NM_001199971	1.87E-08	5.84
Tagln3	transgelin 3	NM_019754	9.15E-10	4.93
Tanc2	tetratricopeptide repeat, ankyrin repeat and coiled-coil 2	XM_006534482	4.00E-08	2.63
Tasp1	taspase, threonine aspartase 1	NM_001159640	1.66E-05	1.81
Tbc1d30	TBC1 domain family, member 30	NM_029057	2.74E-06	2.01
Tbc1d9	TBC1 domain family, member 9	NM_001111304	4.43E-08	1.87
Tceal3	transcription elongation factor A (SII)-like 3	NM_001029978	5.92E-08	2.08
Tekt5	tektin 5	NM_001099275	6.60E-07	2.97
Tenm1	teneurin transmembrane protein 1	NM_011855	5.37E-09	2.80
Tenm2	teneurin transmembrane protein 2	NM_001290702	1.02E-07	2.69
Tenm3	teneurin transmembrane protein 3	NM_001145937	1.24E-06	2.54
Thra	thyroid hormone receptor alpha	NM_178060	2.25E-09	2.20
Thy1	thymus cell antigen 1, theta	NM_009382	3.21E-09	5.16
Timp4	tissue inhibitor of metalloproteinase 4	NM_080639	5.71E-07	3.28
Tle4	transducin-like enhancer of split 4, homolog of Drosophila E	NM_011600	1.22E-06	1.82
Tmc7	transmembrane channel-like gene family 7	NM_172476	6.24E-06	2.13
Tmcc2	transmembrane and coiled-coil domains 2	NM_178874	1.64E-08	2.33
Tmeff1	transmembrane protein with EGF-like and two follistatin-like	NM_021436	3.04E-09	3.35
Tmeff2	transmembrane protein with EGF-like and two follistatin-like domain	NM_019790	9.86E-08	1.86
Tmem130	transmembrane protein 130	NM_177735	7.25E-09	4.18
Tmem145	transmembrane protein 145	NM_183311	6.99E-07	2.07
Tmem150c	transmembrane protein 150C	NM_182841	4.05E-07	2.27
Tmem163	transmembrane protein 163	NM_028135	1.77E-05	2.05
Tmem178b	transmembrane protein 178B	NM_001004182	1.51E-07	3.17
Tmem179	transmembrane protein 179	NM_178915	2.49E-07	2.16
Tmem213	transmembrane protein 213	NM_029921	0.0008157	-1.86
Tmem217	transmembrane protein 217	NM_001162901	0.000313	-1.83
Tmem229a	transmembrane protein 229A	NM_177013	5.41E-08	3.71
Tmem246	transmembrane protein 246	NM_025944	2.72E-09	2.66
Tmem38a	transmembrane protein 38A	NM_144534	3.83E-08	2.36
Tmem38b	transmembrane protein 38B	NM_028053	2.26E-07	-3.56
Tmem47	transmembrane protein 47	NM_138751	4.92E-09	2.01
Tmem59l	transmembrane protein 59-like	NM_182991	3.03E-07	2.01
Tmem63c	transmembrane protein 63c	NM_172583	7.18E-09	2.37
Tmem88b	transmembrane protein 88B	NM_001033394	4.84E-06	3.71
Tmem91	transmembrane protein 91	NM_001290497	2.29E-05	2.74
Tmod2	tropomodulin 2	NM_001038710	5.21E-09	3.12
Tmx4	thioredoxin-related transmembrane protein 4	NM_029148	3.96E-08	2.37
Tnfaip6	tumor necrosis factor alpha induced protein 6	NM_009398	1.66E-06	2.04
Tnfrsf21	tumor necrosis factor receptor superfamily, member 21	NM_178589	5.03E-09	3.81
Tnik	TRAF2 and NCK interacting kinase	NM_001163007	3.78E-07	2.22
Tnk2	tyrosine kinase, non-receptor, 2	NM_001110147	2.92E-07	1.91
Tnr	tenascin R	NM_022312	5.22E-08	2.75
Tpgs2	tubulin polyglutamylase complex subunit 2	NM_001004361	2.75E-10	2.40

S3 Table: Differential changes in adrenal gene expression in ASP+CGP treated mice relative to ASP diet mice (≥±1.8 fold change, p <0.05).

Gene symbol	Gene name	RefSeq	P-value	Fold Change
Tppp	tubulin polymerization promoting protein	NM_182839	9.20E-09	3.95
Tppp3	tubulin polymerization-promoting protein family member 3	NM_026481	8.57E-10	2.15
Trdn	triadin	NM_029726	0.0003912	-1.89
Trh	thyrotropin releasing hormone	NM_009426	1.91E-08	4.60
Tril	TLR4 interactor with leucine-rich repeats	NM_025817	9.68E-08	1.90
Trim2	tripartite motif-containing 2	NM_001271725	3.64E-07	2.14
Trim32	tripartite motif-containing 32	NM_001161782	9.43E-08	2.41
Trim37	tripartite motif-containing 37	NM_197987	3.05E-08	2.41
Trim66	tripartite motif-containing 66	NM_001170912	6.35E-06	1.84
Trim9	tripartite motif-containing 9	NM_001286387	1.47E-10	5.15
Tro	trophinin	NM_001002272	1.95E-07	1.80
Trpc1	transient receptor potential cation channel, subfamily C1	NM_011643	2.78E-09	2.53
Trpm3	transient receptor potential cation channel, subfamily M	NM_001035239	2.96E-07	1.98
Tspan13	tetraspanin 13	NM_025359	2.53E-08	2.47
Tspan2	tetraspanin 2	NM_001243132	1.64E-06	2.28
Tspan7	tetraspanin 7	NM_019634	3.41E-10	2.42
Tspyl2	TSPY-like 2	NM_029836	1.62E-07	1.94
Tspyl4	TSPY-like 4	NM_030203	2.89E-11	3.86
Ttbk2	tau tubulin kinase 2	NM_001024856	8.45E-07	1.83
Ttc3	tetratricopeptide repeat domain 3	NM_009441	1.07E-08	2.25
Ttc9	tetratricopeptide repeat domain 9	NM_001033149	2.75E-07	3.44
Ttll1	tubulin tyrosine ligase-like 1	NM_178869	6.25E-07	2.37
Ttll7	tubulin tyrosine ligase-like family, member 7	NM_027594	2.76E-07	2.35
Ttyh1	tweety homolog 1 (Drosophila)	NM_001001454	6.87E-11	11.71
Tuba1a	tubulin, alpha 1A	NM_011653	1.42E-07	2.87
Tuba4a	tubulin, alpha 4A	NM_009447	1.38E-08	2.46
Tubb2a	tubulin, beta 2A class IIA	NM_009450	1.23E-08	3.86
Tubb2b	tubulin, beta 2B class IIB	NM_023716	3.03E-08	4.05
Tubb3	tubulin, beta 3 class III	NM_023279	2.18E-07	4.22
Tubb4a	tubulin, beta 4A class IVA	NM_009451	2.02E-14	11.69
Tubg2	tubulin, gamma 2	NM_134028	2.87E-06	2.62
Tulp4	tubby like protein 4	NM_001103181	3.51E-07	2.12
Tvp23a	trans-golgi network vesicle protein 23A	NM_001013778	1.78E-07	2.31
Ube2o	ubiquitin-conjugating enzyme E2O	NM_173755	9.07E-06	1.86
Ube2ql1	ubiquitin-conjugating enzyme E2Q family-like 1	NM_001145162	8.08E-11	6.58
Ubqln2	ubiquilin 2	NM_018798	1.12E-07	1.90
Uchl1	ubiquitin carboxy-terminal hydrolase L1	NM_011670	5.64E-09	2.04
Ugt8a	UDP galactosyltransferase 8A	NM_011674	3.55E-06	2.19
Unc13a	unc-13 homolog A (C. elegans)	NM_001029873	1.42E-06	1.93
Unc13c	unc-13 homolog C (C. elegans)	NM_001081153	3.53E-08	5.16
Unc79	unc-79 homolog (C. elegans)	NM_001081017	3.85E-07	1.90
Unc80	unc-80 homolog (C. elegans)	NM_175510	9.88E-08	3.12
Usp11	ubiquitin specific peptidase 11	NM_145628	1.03E-06	1.94
Usp22	ubiquitin specific peptidase 22	NM_001004143	2.49E-07	1.82
Usp29	ubiquitin specific peptidase 29	NM_001290994	7.07E-06	2.46
Usp46	ubiquitin specific peptidase 46	NM_177561	5.01E-06	1.92
Utp14b	UTP14, U3 small nucleolar ribonucleoprotein, homolog B	NM_001001981	4.30E-07	2.32
Vamp1	vesicle-associated membrane protein 1	NM_001080557	7.99E-08	2.98
Vat1l	vesicle amine transport protein 1 homolog-like (T. californica)	NM_173016	3.10E-08	3.03
Vgf	VGF nerve growth factor inducible	NM_001039385	1.19E-06	1.98
Vsnl1	visinin-like 1	NM_012038	1.94E-08	3.13

S3 Table: Differential changes in adrenal gene expression in ASP+CGP treated mice relative to ASP diet mice (≥±1.8 fold change, p <0.05).

Gene symbol	*Gene name*	*RefSeq*	*P-value*	*Fold Change*
Vstm2a	V-set and transmembrane domain containing 2A	NM_001290539	6.62E-09	15.44
Vstm2b	V-set and transmembrane domain containing 2B	NM_021387	1.35E-06	1.96
Vtn	vitronectin	NM_011707	4.36E-08	2.45
Vwc2l	von Willebrand factor C domain-containing protein 2-like	NM_177164	6.63E-06	1.87
Wasf1	WAS protein family, member 1	NM_031877	1.98E-07	2.93
Wdr17	WD repeat domain 17	NM_001172152	1.41E-06	2.14
Wdr47	WD repeat domain 47	NM_181400	2.79E-07	2.49
Wdr7	WD repeat domain 7	NM_001014981	1.04E-08	1.90
Wnk1	WNK lysine deficient protein kinase 1	NM_001199083	7.95E-06	2.04
Wnk2	WNK lysine deficient protein kinase 2	NM_001290311	1.92E-07	2.36
Wscd1	WSC domain containing 1	NM_177618	5.55E-06	1.85
Zcchc12	zinc finger, CCHC domain containing 12	NM_028325	2.86E-08	4.51
Zcchc18	zinc finger, CCHC domain containing 18	NM_001035509	6.13E-07	2.36
Zcchc24	zinc finger, CCHC domain containing 24	NM_001101433	6.23E-07	1.93
Zdhhc2	zinc finger, DHHC domain containing 2	NM_178395	1.92E-05	2.19
Zdhhc22	zinc finger, DHHC-type containing 22	NM_001080943	2.20E-09	3.49
Zdhhc23	zinc finger, DHHC domain containing 23	NM_001007460	0.0005871	1.87
Zfp365	zinc finger protein 365	NM_178679	1.64E-08	2.68
Zfp385a	zinc finger protein 385A	NM_013866	7.79E-07	1.88
Zfp385b	zinc finger protein 385B	NM_178723	2.52E-05	2.06
Zfp385c	zinc finger protein 385C	NM_177790	2.49E-05	-1.87
Zfp804a	zinc finger protein 804A	NM_175513	3.02E-07	2.63
Zfp941	zinc finger protein 941 E197E1:E243	NM_001001180	2.19E-05	1.84
Zic1	zinc finger protein of the cerebellum 1	NM_009573	1.85E-08	4.26
Zic2	zinc finger protein of the cerebellum 2	NM_009574	7.17E-07	4.02
Zkscan16	zinc finger with KRAB and SCAN domains 16	NM_001099323	1.99E-07	2.86
Zmat4	zinc finger, matrin type 4	NM_001277239	9.35E-09	2.31
Zwint	ZW10 interactor	NM_001293683	2.35E-12	2.83
Zyg11b	zyg-ll family member B, cell cycle regulator	NM_001033634	2.75E-06	2.01
